# Heavy Metal Tolerance in Plants: Role of Transcriptomics, Proteomics, Metabolomics, and Ionomics

**DOI:** 10.3389/fpls.2015.01143

**Published:** 2016-02-08

**Authors:** Samiksha Singh, Parul Parihar, Rachana Singh, Vijay P. Singh, Sheo M. Prasad

**Affiliations:** ^1^Ranjan Plant Physiology and Biochemistry Laboratory, Department of Botany, University of AllahabadAllahabad, India; ^2^Department of Botany, Government Ramanuj Pratap Singhdev Post Graduate College, Sarguja UniversityBaikunthpur, India

**Keywords:** crop, heavy metal, ionomics, metabolomics, metallophytes, proteomics, transcriptomics, yield

## Abstract

Heavy metal contamination of soil and water causing toxicity/stress has become one important constraint to crop productivity and quality. This situation has further worsened by the increasing population growth and inherent food demand. It has been reported in several studies that counterbalancing toxicity due to heavy metal requires complex mechanisms at molecular, biochemical, physiological, cellular, tissue, and whole plant level, which might manifest in terms of improved crop productivity. Recent advances in various disciplines of biological sciences such as metabolomics, transcriptomics, proteomics, etc., have assisted in the characterization of metabolites, transcription factors, and stress-inducible proteins involved in heavy metal tolerance, which in turn can be utilized for generating heavy metal-tolerant crops. This review summarizes various tolerance strategies of plants under heavy metal toxicity covering the role of metabolites (metabolomics), trace elements (ionomics), transcription factors (transcriptomics), various stress-inducible proteins (proteomics) as well as the role of plant hormones. We also provide a glance of some strategies adopted by metal-accumulating plants, also known as “metallophytes.”

## Introduction

During the last few decades, increased anthropogenic activities, rapid industrialization, and modern agricultural practices have resulted in increased heavy metal contamination in the environment, which causes toxicity to the living organisms (Eapen and D'souza, 2005; Kavamura and Esposito, 2010; Miransari, 2011). Large areas of land have been contaminated with heavy metals due to the use of pesticides, fertilizers, municipal and compost wastes, and also due to heavy metal release from smelting industries and metalliferous mines (Yang et al., 2005). Although many heavy metals occur naturally in the earth's crust at various levels, the problem arises when they are released in excess into the environment due to natural and/or anthropogenic activities. The 53 elements belonging to the d-block have been categorized as “heavy metals” based on their density (>5 g/cm^3^) (Jarup, 2003). During evolution of angiosperms, only 19 elements such as C, O, H, Mg, S, N, Ca, P, and K (macronutrients) and Cu, Zn, Mn, Fe, Mo, B, Ni, Co, Cl, and B (micronutrients) were selected for basic metabolism (Ernst, 2006). In addition, Si is also considered as a beneficial element, and it has been reported to be involved in the maintenance of plant structures in some plants (Epstein, 1999). Macro and micronutrients play an important role in physiological and biochemical processes of plants such as chlorophyll biosynthesis, photosynthesis, DNA synthesis, protein modifications, redox reactions in the chloroplast and the mitochondrion, sugar metabolism, and nitrogen fixation. For example, Zn is a cofactor for more than 300 enzymes and 200 transcription factors associated with the maintenance of membrane integrity, auxin metabolism, and reproduction (Marschner, 1995; Barker and Pilbeam, 2007; Briat et al., 2007; Williams and Pittman, 2010; Prasad, 2012; Ricachenevsky et al., 2013). However, at elevated concentrations, heavy metals produce severe toxicity symptoms in plants, and therefore, their uptake and utilization are tightly controlled by the plant cells (Janicka-Russak et al., 2008; Saito et al., 2010; Singh et al., 2012; Srivastava et al., 2012; DalCorso et al., 2013a; Farias et al., 2013; Fidalgo et al., 2013). Some heavy metals, such as Cd, Cr, Pb, Al, Hg, etc., although being non-essential and without physiological function, are very toxic even at very low concentrations (Ernst et al., 2008; Janicka-Russak et al., 2008; Garzón et al., 2011; Hayat et al., 2012; Shahid et al., 2012; Chong-qing et al., 2013; Gill et al., 2013). Essential and non-essential heavy metals generally produce common toxic effects on plants, such as low biomass accumulation, chlorosis, inhibition of growth and photosynthesis, altered water balance and nutrient assimilation, and senescence, which ultimately cause plant death.

In addition to adverse impacts on plants, heavy metals pose threat to human health due to their persistence in nature. For instance, Pb is one of the most toxic heavy metals that has soil retention time of 150–5000 years and reported to maintain its concentration high for as long as 150 years (NandaKumar et al., 1995; Yang et al., 2005). Plants growing in heavy metal-contaminated sites generally accumulate higher amounts of heavy metals, and thus, contamination of food chain occurs. Contaminated food chain acts as a primary route for the entry of heavy metals into animal and human tissues, making them prone to several diseases that range from dermatitis to various types of cancers (McLaughlin et al., 1999). This problem might become even worse if sufficient measures are not taken at the right time. Therefore, research in this area is driven by the hope to decrease the entry of heavy metals in crop plants, thereby reducing the risk of contamination in animals and human beings.

**Figure 1:**
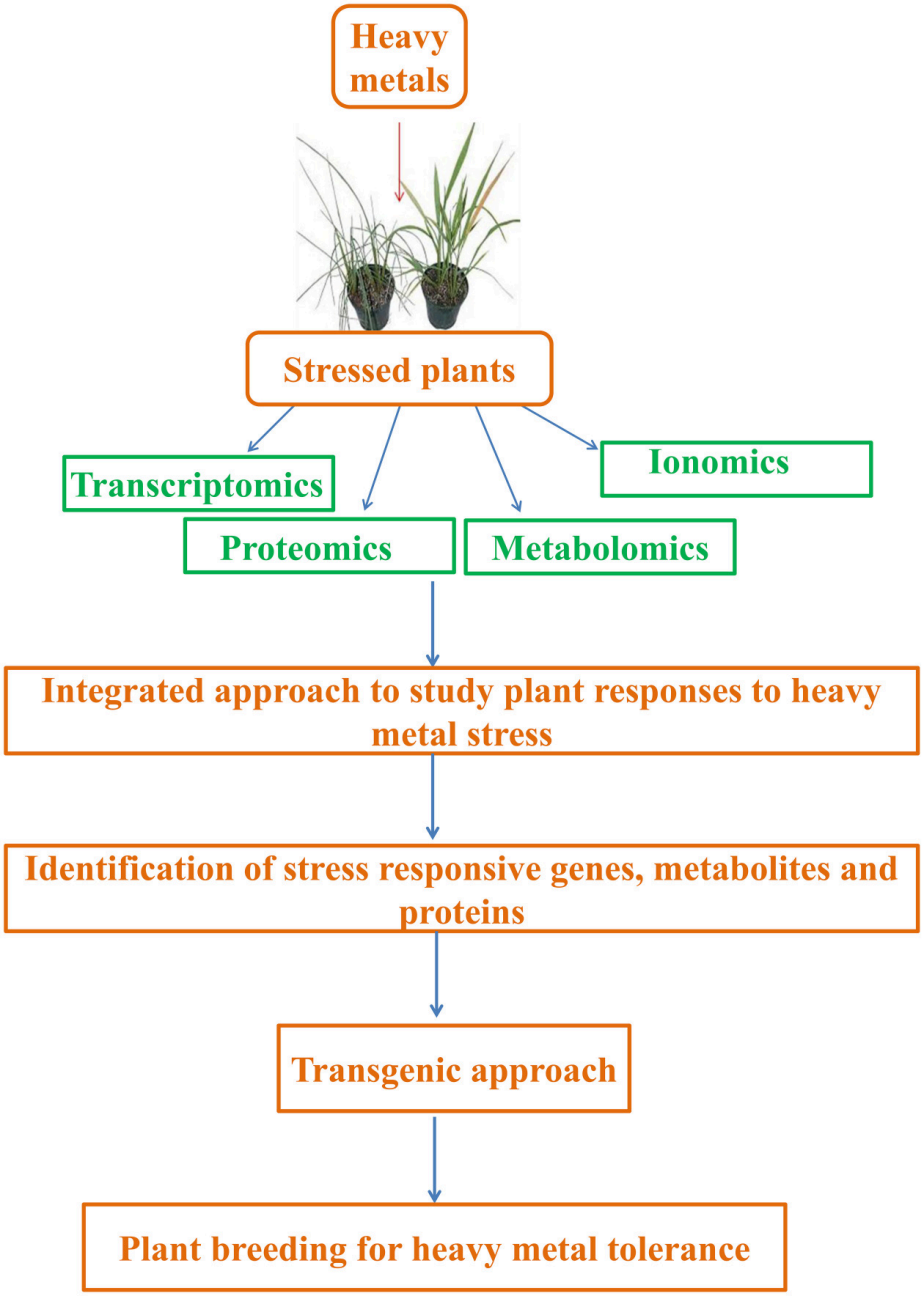
**Integrated approach to study plant responses to heavy metal stress**. Transcriptomics, proteomics, metabolomics, and ionomics are useful tools that can help us to decipher and analyze active regulatory networks controlling heavy metal stress responses and tolerance.

Abiotic stresses are estimated to be the main cause for global crop yield reduction of ca. 70%, and thus, are considered a great constraint to crop production (Acquaah, 2007; Jewell et al., 2010). This situation has worsened due to disturbed equilibrium between crop productivity and population growth. Therefore, it is especially important to understand plants' responses to such stressors, particularly heavy metals, in order to find new methods for improving crops quantitatively and qualitatively. Currently, studies are being performed to address the above mentioned problems and have majorly focused on “omic” tools that take into consideration of ionomics (trace elements), metabolomics (metabolome), transcriptomics (transcriptome), and proteomics (proteome). The data obtained will provide insights that might help in enhancing stress tolerance and be employed in breeding and engineering programs aiming at developing plants with new and desired agronomical traits (Lee et al., 2007; Atkinson and Urwin, 2012). In this context, this review is focused on several aspects, from plant responses to heavy metals (considering sensitive as well as metallophytes) to the role of ionomics, metabolomics, transcriptomics, and proteomics in the regulation of heavy metal tolerance (Figure 1).

## Plant responses to heavy metal stress

Being sessile organisms, plants cannot escape unwanted changes in the environment. Exposure to heavy metals triggers a wide range of physiological and biochemical alterations, and plants have to develop and/or adopt a series of strategies that allow them to cope with the negative consequences of heavy metal toxicity. Plants respond to external stimuli including heavy metal toxicity *via* several mechanisms. These include (i) sensing of external stress stimuli, (ii) signal transduction and transmission of a signal into the cell, and (iii) triggering appropriate measures to counterbalance the negative effects of stress stimuli by modulating the physiological, biochemical, and molecular status of the cell. At the whole plant level, it is difficult to measure sensing and changes in the signal transduction after exposing plants to heavy metal stress. However, monitoring early responses, such as oxidative stress, transcriptomic and proteomic changes, or accumulation of metabolites, might be useful to study sensing and signal transduction changes that take place after plants' exposure to stress. For instance, Tamás et al. (2010) reported that early signs of metal toxicity in barley were similar to water deficiency signs, and thus, overexpression of genes related to dehydration stress in barley was found after exposure to Cd and Hg. Similar to this, Hernandez et al. (2012) reported oxidative stress and glutathione depletion in alfalfa roots as early signs of sensing and signal transduction after exposure to heavy metals. In another study by Zhang et al. (2002), seed germination and seedling growth of wheat was found to be inhibited due to high concentration of As. Similarly, Imran et al. (2013) reported reduction in plumule and radicle length of *Helainthus annuus* L. seedlings when exposed to As. In addition, As has also been reported to decrease the photosynthetic pigment, damage chloroplast membrane, and decrease enzyme activity by reacting with the sulfhydryl group of proteins and also reported to alter nutrient balance and protein metabolism (Li et al., 2006; Singh et al., 2009; Ahsan et al., 2010).

Heavy metals exert toxicities in plants through four proposed mechanisms. These include (i) similarities with the nutrient cations, which result into a competition for absorption at root surface; for example, As and Cd compete with P and Zn, respectively, for their absorption; (ii) direct interaction of heavy metals with sulfhydryl group (-SH) of functional proteins, which disrupts their structure and function, and thus, renders them inactive; (iii) displacement of essential cations from specific binding sites that lead to a collapse of function; and (iv) generation of reactive oxygen species (ROS), which consequently damages the macromolecules (Sharma and Dietz, 2009; DalCorso et al., 2013a).

The roots of sessile plants are the first organ that encounters heavy metals, and thus, roots have been widely studied to assess the impact of a stressor. Plants growing on heavy metal-rich soils suffer from both decreased growth and yield (Keunen et al., 2011), indicating an implication of heavy metal toxicity in hampering the overall growth performance of the stressed plants (Kikui et al., 2005; Panda et al., 2009; Buendía-González et al., 2010; Gangwar et al., 2010, 2011; Gangwar and Singh, 2011; Eleftheriou et al., 2012; Hayat et al., 2012; Silva, 2012; Anjum et al., 2014). Root growth is a combination of cell division and elongation. In this context, a decrease in mitotic activity has been reported in several plant species after exposure to heavy metals, which consequently results into a suppressed root growth (Fontes and Cox, 1998; Doncheva et al., 2005; Sundaramoorthy et al., 2010; Hossain et al., 2012a,b; Thounaojam et al., 2012). A study by Liu et al. (1992) showed that Cr(VI) has greater toxic effect on cell division than Cr(III). Furthermore, Sundaramoorthy et al. (2010) have also observed that Cr(VI) caused an extension in cell cycle that leads to the inhibition in cell division, thereby reducing root growth.

Pena et al. (2012) have reported that Cd toxicity affects the cell cycle G1/S transition and progression through S phase *via* decreased expression of a cyclin-dependent kinase (CDK), suggesting that ROS might be involved in such alterations. Yuan et al. (2013) have reported that excess Cu affects both elongation and meristem zones by altering auxin distribution through PINFORMED1 (PIN1) protein, and that Cu-mediated auxin redistribution is responsible for Cu-mediated inhibition of primary root elongation. Similarly, Petö et al. (2011) have also demonstrated that excess Cu inhibits root length and alters morphology by inducing alterations in auxin levels, which antagonizes nitric oxide function. It has also been demonstrated that inhibition in root growth is accompanied by an increase in root diameter, suggesting that plant cytoskeleton might also be a target of heavy metal toxicity (Zobel et al., 2007). Therefore, these studies suggest that heavy metals might cause an inhibition in root growth that alters water balance and nutrient absorption, thereby affecting their transportation to the aboveground plant parts and thus negatively affecting shoot growth and ultimately decreasing biomass accumulation. Roots utilize several mechanisms such as synthesis and deposition of callose to reduce and/or avoid heavy metal toxicity. These mechanisms create a barrier for the entry of heavy metals and enhance plasticity of root anatomy. Apart from barricading the entry of heavy metals, roots also allow their transportation to aboveground plant parts (in the case of metallophytes or hyperaccumulator plants: plants that can grow in heavy metal-contaminated soil; Fahr et al., 2013) for sequestration into the vacuoles rendering them inactive, and thus non-reactive.

Plasma membranes serve as a highly regulated checkpoint for an entry of unwanted substances inside the cell and protect the cell from negative consequences of many stressors. It has been reported that *Arabidopsis halleri* and *Arabidopsis arenosa* were more tolerant to heavy metal stress than *Arabidopsis thaliana* due to the lowest membrane depolarization, indicating that rapid membrane voltage changes might be an excellent tool for monitoring the effects of heavy metal toxicity (Kenderešová et al., 2012). Once inside the cell, heavy metals alter metabolism that results into a reduction of growth and lower biomass accumulation (Nagajyoti et al., 2010). Heavy metal toxicity might also cause stunted stem and root length, and chlorosis in younger leaves that can extend to the older leaves after prolonged exposure (Israr et al., 2006; Guo et al., 2008a,b; Warne et al., 2008; Gangwar and Singh, 2011; Gangwar et al., 2011; Srivastava et al., 2012). At the cellular and molecular levels, heavy metal toxicity affects plants in many ways. For instance, it alters the key physiological and biochemical processes such as seed germination, pigment synthesis, photosynthesis, gas exchanges, respiration, inactivation and denaturation of enzymes, blocks functional groups of metabolically important molecules, hormonal balance, nutrient assimilation, protein synthesis, and DNA replication (Nagajyoti et al., 2010; Yadav, 2010; Keunen et al., 2011; He et al., 2012; Hossain et al., 2012a,b; Silva, 2012; Wani et al., 2012; Singh et al., 2013). Under Cd stress, severe deleterious effects on various photosynthetic indices such as photosynthetic rate (Pn) and intracellular CO_2_ concentration (Ci) have been reported in tomato seedlings (Dong et al., 2005). Maleva et al. (2012) have observed that Mn, Cu, Cd, Zn, and Ni caused a significant decline in the levels of chlorophyll contents, accompanied by a decrease in the photochemical efficiency of photosystem II (PS II) in *Elodea densa*. Similarly, Li et al. (2012) have also reported that Cu, Zn, Pb, and Cd depressed chlorophyll and carotenoids levels and the quantum yield of PS II in *Thalassia hemprichii*, indicating that heavy metals have negative consequences on photosynthesis. Apart from affecting light reactions, heavy metals decrease CO_2_ assimilation by either diminishing RUBP carboxylase activity or by reacting with the thiol group of RUBISCO. For instance, Zn has been reported to inhibit RUBISCO activity in *Phaseolus vulgaris* by replacing Zn^+2^ for Mg^+2^, as both are bivalent cations (Monnet et al., 2001). In another study on *Erythrina variegate* by Muthuchelian et al. (2001), decreased RUBISCO activity was observed under Cd stress, and this decrease in RUBISCO activity might be due to the formation of mercaptide by Cd with thiol group of RUBISCO (Siborova, 1988). These researchers also reported decreased CO_2_ fixation, which is possibly due to a decrease in ATP and reductant pool (Husaini and Rai, 1991), as Cd ions decrease the proton source for reduction reactions (Ferretti et al., 1993). Similarly, Cu, a well-known inhibitor of carboxylase and oxygenase activities of RUBISCO (Lidon and Henriques, 1991), was found to decrease RUBISCO activity in *Chenopodium rubrum* (Schafer et al., 1992) by interacting with the essential cysteine residue of the enzyme (Siborova, 1988). Such reduction in pigments, photosynthetic rate, quantum yield of PS II, gas exchange, stomatal conductance, and CO_2_ assimilation might be linked to the ultrastructural changes (changes at cellular and tissue levels) induced by heavy metal stress. The effects arising due to changes in ultrastructures of membranes have been reported in several studies (Azzarello et al., 2012; Basile et al., 2012; Esposito et al., 2012; He et al., 2012; Sánchez-Pardo et al., 2012; Ali et al., 2013a,b). Moreover, heavy metals have been reported to affect another key physiological process, i.e., nitrogen metabolism, which is involved in plant function, from metabolism to allocation of resources, thereby regulating plant growth and development. Heavy metals have been found to enhance protease activity (Chaffei et al., 2003), and thus, reducing the activity of enzymes involved in nitrate (Nitrate reductase; NR and Nitrite reductase; NiR) and ammonia (Glutamine synthetase; GS, Glutamine oxoglutarate aminotransferase; GOGAT and Glutamate dehydrogenase; GDH) assimilation. The heavy metal Cd has been reported to affect nitrogen metabolism by inhibiting nitrate uptake and transportation, nitrate reductase, and GS activity (Hernández et al., 1997; Lea and Miflin, 2004), thereby affecting primary N assimilation processes.

Heavy metal-mediated alteration in hormonal balance correlates with their toxicities in plants (Petö et al., 2011; Wilkinson et al., 2012). For instance, in *Brassica juncea*, As causes toxicity by changing the levels of the auxins:indole-3-acetic acid (IAA), indole-3- butyric acid (IBA), and naphthalene acetic acid (NAA) and altering the expression of about 69 microRNAs (Srivastava et al., 2013). However, exogenous supply of IAA improves the growth of *B. juncea* under As stress, suggesting an implication of the regulation of the hormone level in the management of As stress.

### Metallophytes under heavy metal stress

**Figure 2:**
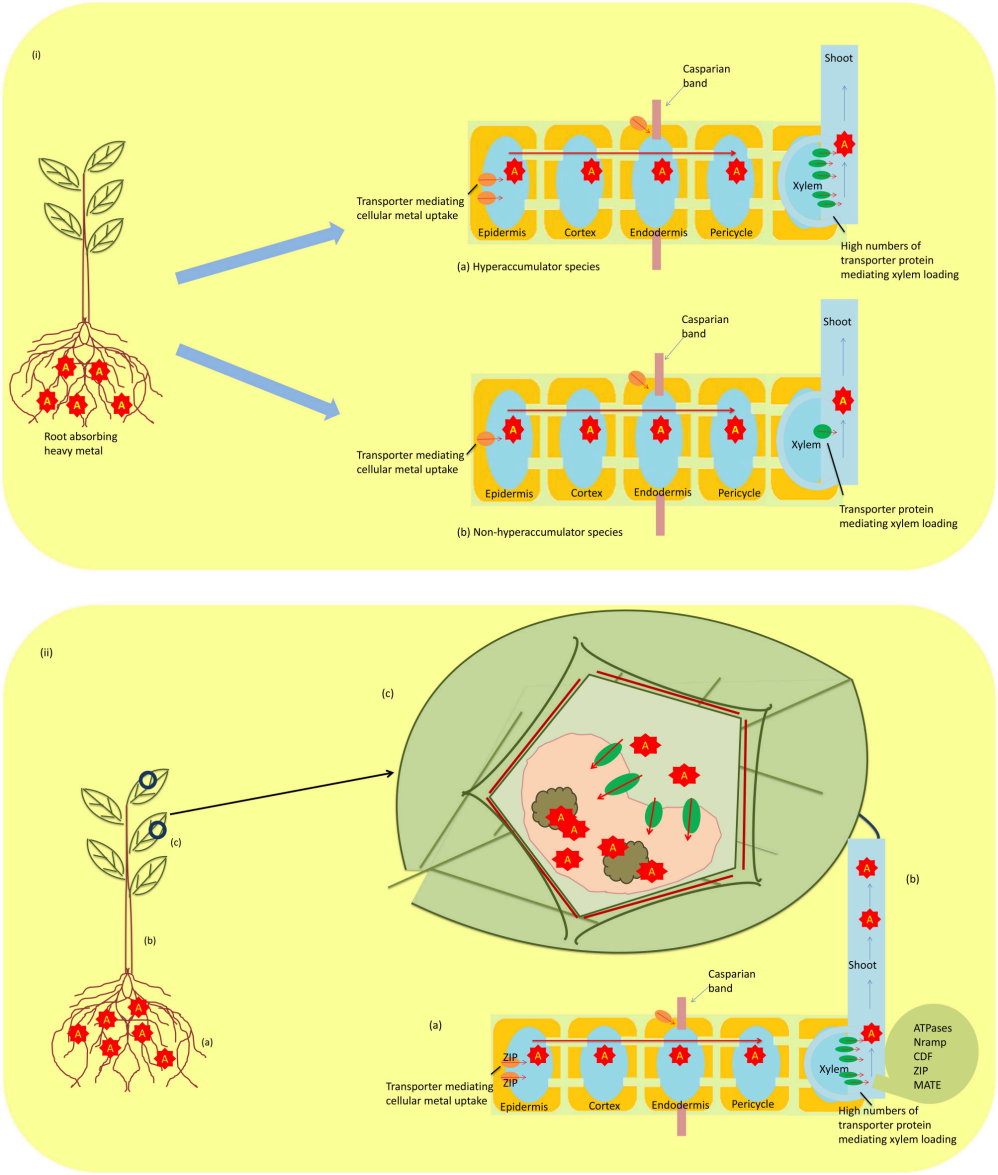
**(i)** A schematic diagram showing differential expression of constitutive gene in regulating transporters in hyperaccumulating **(a)** and non-hyperaccumulating **(b)** species (A; heavy metal). **(ii)** Mechanism of metal tolerance in hyperaccumulators **(a)** metal uptake by roots; ZIP (zinc-regulated transporter iron-regulated transporter proteins) **(b)** translocation of metal from root to shoot; ATPases (or CPx-type, P1B-type), Nramp (natural resistance-associated macrophage protein), CDF (cation diffusion facilitator family proteins), zinc–iron permease (ZIP) family proteins, MATE (Multidrug and Toxin Efflux) family, and **(c)** sequestration of metal in vacuole. A, metal; B, organic acid.

Metallophytes, also known as hyperaccumulators, have the ability to uptake large amounts of heavy metals from the soil, and this property makes them unique to be utilized in technologies such as biogeochemical and biogeobotanical prospection and phytoremediation. The absorbed heavy metals from the soil by these hyperaccumulators are not retained in the roots but are translocated to the shoots and accumulated in the aboveground organs at concentrations 100–1000-fold higher than the observed in non-hyperaccumulating species (Figures 2ia,b). However, this high concentration does not pose any toxic effect on plants (Rascio, 1997; Reeves, 2006; Prasad et al., 2010). With significant advances in our understanding of the mechanisms adopted by hyperaccumulators, there has been implication of three hallmarks that distinguish them from non-hyperaccumultors. These are (i) greater capability of heavy metal uptake, (ii) root-to-shoot translocation of heavy metal, and (iii) detoxification and sequestration of heavy metal (Figures 2iia–c). Studies on *Thlaspi caerulescens* and *A. halleri*, model plants for studying heavy metal tolerance strategies, have been done (Milner and Kochian, 2008; Singh et al., 2009; Frérot et al., 2010; Krämer, 2010). The studies have revealed that hyperaccumulation is not due to the presence of a novel gene, but it arises only from differential expression of genes that are common to hyperaccumulators and non-hyperaccumulators (Verbruggen et al., 2009). Hyperaccumulation of heavy metal includes three complex phenomena discussed below:

#### Heavy metal uptake

Hyperaccumulators have an extraordinary ability to absorb heavy metals from the soil under varying concentration of heavy metals (Ma et al., 2001; Yang et al., 2002). Although heavy metals are taken up by hyperaccumulators, their uptake is affected by several factors such as pH, water content, organic substances, etc. Moreover, heavy metal uptake requires a suitable transporting system to enter the plant (Figure 2i). Several researchers have reported that pH affects (i) proton secretion by roots that further acidify rhizosphere, thus enhancing metal dissolution, and (ii) the growth of metal-accumulating plant species (Bernal et al., 1994; Peng et al., 2005; Kuriakose and Prasad, 2008). Apart from pH, organic substances released from the roots affect growth in hyperaccumulating plants. Krishnamurti et al. (1997) have reported that organic acids released influence Cd solubility by forming Cd complexes. Therefore, pH and organic substances released from the rhizosphere of a hyperaccumulator mobilize heavy metal and enhance absorption (Krishnamurti et al., 1997; Peng et al., 2005). High uptake of heavy metal has also been associated with enhanced root proliferation (Whiting et al., 2000). Furthermore, constitutive overexpression of genes also attributes to enhanced heavy metal uptake. To pinpoint the genes involved in overexpression, several comparative studies have been performed in hyperaccumulating *Arabidopsis halleri* and *Thlapsi caerulescens* with that of congener non-hyperaccumulating species. Studies on *T. caerulescens* and *A. halleri* have revealed that increased Zn uptake is due to overexpression of genes belonging to the ZIP (Zinc-regulated transporter Iron-regulated transporter proteins) family encoding plasma membrane located transporters (Assunção et al., 2001): ZTN1 and ZTN2 in *T. caerulescens* and ZIP6 and ZIP9 in *A. halleri*. The decreased uptake of Cd under increasing Zn concentration was noticed in both genera, and it clearly demonstrated that expression of ZIP genes is Zn regulated (Assunção et al., 2010) and Cd influx is mainly due to Zn transporters having strong preference for Zn over Cd (Weber et al., 2006). Evidence exists that As being a chemical analog of phosphate enters the plant cell *via* phosphate transporters (Meharg and Hartley-Whitaker, 2002; Kanoun-Boulé et al., 2009). Similarly, a study on the As hyperaccumulator *Pteris vittata* and non-hyperaccumulator *Pteris tremula* has shown that plasma membranes of root cells of *P. vittata* had high density of phosphate/arsenate transporters than *P. tremula* (Caille et al., 2005), possibly due to constitutive gene overexpression. In addition, a study on the Se hyperaccumulators *Astragalus bisulcatus* (Fabaceae) and *Stanleya pinnata* (Brassicaceae) revealed that there was a higher Se/S ratio in the shoots of these species in comparison to the non-hyperaccumulator sister species, and this observation also supported the fact that an enhanced Se uptake was through sulfate transporters (Galeas et al., 2007).

#### Root-to-shoot translocation of heavy metals

Unlike non-hyperaccumulator plants, hyperaccumulators do not retain the heavy metal absorbed from roots but translocate them into shoots *via* xylem and several classes of proteins are involved in this translocation. The proteins involved are heavy metal-transporting ATPases (or CPx-type, P1B-type), natural resistance-associated macrophage proteins (Nramp), cation diffusion facilitator (CDF) family proteins, zinc–iron permease (ZIP) family proteins, and MATE (Multidrug And Toxin Efflux) protein family. The CPx-type ATPases are involved in transporting toxic metals like Cu, Zn, Cd, and Pb using ATP across cell membranes (Williams et al., 2000). The P1B-type ATPases also have the similar role of transporting heavy metal, but they also regulate metal homeostasis as well as tolerance (Axelsen and Palmgren, 1998). These heavy metal ATPases (HMAs) overexpressed in roots and shoots of hyperaccumulators suggest their upregulation in hyperaccumulators in comparison to non-hyperaccumulators (Papoyan and Kochian, 2004). Nramp is another class of protein family that has been found to be involved in transporting heavy metal ions, and genes coding for these proteins are termed as *Nramp* genes. Studies on rice revealed three homologs of this protein, namely OsNramp1, OsNramp2, and OsNramp3, and these proteins expressed in different tissues of rice transport distinct but related ions (Belouchi et al., 1997). Another class of proteins (CDF) have been found to be involved in transporting Zn, Co, and Cd, and regulate effluxing of cation out of the cytoplasmic compartment, and therefore, they are termed as “cation efflux transporters” (Mäser et al., 2001). A related Zn transporter (ZNT1) from *T. caerulescens* was reported by Pence et al. (2000), which belongs to a superfamily known as ZIP gene family, and was found to be expressed at high levels in roots and shoots. Another Zn transporter ZAT1 was also reported in *Arabidopsis* that was highly expressed in root tissues (van der Zaal et al., 1999). The transporter protein MATE is also involved in heavy metal translocation; FDR3, a protein of this family, was found to be expressed in roots of *T. caerulescens* and *A. halleri*, and the gene encoding this protein FDR3 plays a role in translocation of heavy metal (Talke et al., 2006; van de Mortel et al., 2006; Krämer et al., 2007). Therefore, the abovementioned studies provide strong evidence that multiple transporter proteins are involved in the translocation of heavy metal.

#### Detoxification/sequestration of heavy metal

After translocating, hyperaccumulators sequestrate and then detoxify the heavy metal, a process that allows them to survive under metal-contaminated areas without suffering from any toxic effect (Figure 2ii). The process of detoxification/sequestration occurs in the vacuole of plants (Vögeli-Lange and Wagner, 1990; Kanoun-Boulé et al., 2009; Singh et al., 2011a) and several transporter families are involved in this process, namelyABC, CDF, HMA, and NRAMP transporters. The ABC transporters are involved in transporting heavy metal into the vacuole and mainly two subfamilies (MRP and PDR) are active. The HMT1, first vacuolar ABC transporter reported in *Schizosaccharomyces pombe*, localized in the tonoplast aids in transporting PC–Cd (phytochelatins–cadmium) complexes formed in the cytosol (Ortiz et al., 1992, 1995; Kuriakose and Prasad, 2008). Later on, a functional homolog of HMT1 has been reported in *Caenorhabditis elegans* and Drosophila (Vatamaniuk et al., 2005; Sooksa-Nguan et al., 2009); however, no such homolog was studied in plants. Studies in *A. thaliana* have revealed two transporters AtMRP1 and AtMRP2 in transporting PC–Cd complexes into the vacuole (Lu et al., 1997, 1998), and these transporters confer the metal tolerance. The CDF transporter family, also named “metal tolerance protein (MTP),” is also involved in transporting metal cations such as Zn^2+^, Cd^2+^, Co^2+^, Ni^2+^, or Mn^2+^ from the cytosol to the vacuole (Krämer et al., 2007; Montanini et al., 2007). They have been categorized into two of four distinct groups of which groups I and III are the most important (Blaudez et al., 2003). Comparative studies in *A. halleri* and *T. caerulescens* with those of non-hyperaccumulators have shown higher expression of MTP1 (group III), MTP8 (group I), and MTP11 (group I) (Becher et al., 2004; Talke et al., 2006; van de Mortel et al., 2006). Similarly, AhMTP1 protein also showed a constitutive higher expression in leaves of *A. halleri* under exogenous supply of Zn (Dräger et al., 2004). The MTP11 and MTP8 were found to be close homologs of ShMTP8 (formerly ShMTP1) and confirmed Mn tolerance in *A. thaliana* (Delhaize et al., 2003), thus suggesting a role of these proteins in metal tolerance. Likewise, other transporter proteins such as HMA and NRAMP are also involved in transporting the metal from the cytosol to the vacuole. However, HMAs are thought to be involved in detoxification mechanisms due to their overexpression, as reported in *A. thaliana* (Morel et al., 2009).

Apart from the role of transporter proteins, organic acids are also involved in detoxification mechanisms, as they help in entrapping the metal ion and chelating them. For instance, citrate binds with Ni in leaves of *Thlaspi goesingense*, enabling formation of metal–organic acid complex for chelation (Krämer et al., 2000). Similarly, malate binds with Zn in *A. halleri* and Cd in *T. caerulescens* (Salt et al., 1999; Sarret et al., 2002). The role of amino acids in hyperaccumulator has been found to be important due to the formation of stable complexes with bivalent cations (Callahan et al., 2006), thus helping largely in sequestrating metal cations. For example, histidine (His) is involved in Ni hyperaccumulation, and a high concentration of His has been reported in the roots of Ni hyperaccumulators (Assuncão et al., 2003). The mechanism of heavy metal detoxification in hyperaccumulators also relies on the overexpression of genes related with antioxidant activity such as reduced glutathione (GSH), cysteine and *O*-acetylserine (Anjum et al., 2014). Studies have revealed that upstream signaling of salicylic acid results in increased serine acetyltransferase (SAT) activity and higher GSH level (Freeman et al., 2005a). Similarly, overexpression of *NgSAT* in *Noccaea goesingense* resulted in enhanced levels of GSH that resulted in Ni, Co, Zn, and to a small extent Cd tolerance (Freeman et al., 2004; Freeman and Salt, 2007).

## Heavy metal and reactive oxygen species (ROS) production

Another negative consequence of heavy metal accumulation is the generation of ROS. In plants, ROS accumulation depends upon the balance between ROS production and ROS scavenging (Mittler et al., 2004), which in turn also depends on growth conditions such as temperature, light intensity, presence of heavy metal, etc. For instance, the presence of excess heavy metals results into a limitation of CO_2_ fixation in the chloroplasts, which coupled with an over reduction of the photosynthetic electron transport chain serves as a major site of ROS production (Mittler et al., 2004). Over reduction of the electron transport chains in the mitochondria is also a major site of ROS generation (Davidson and Schiestl, 2001; Keunen et al., 2011). Møller et al. (2007) reported that 1–5% of O_2_ consumed by the isolated mitochondria converts into ROS. ROS hydrogen peroxide (H_2_O_2_) is produced in the peroxisomes after glycolate is oxidized to glyoxylic acid during photorespiration (Mittler et al., 2004). Therefore, ROS such as singlet oxygen (^1^O_2_), superoxide anion (${\text{O}}_{2}^{∙-}$), H_2_O_2_, and hydroxyl radicals (^∙^OH) are produced in these organelles because of spin inversion and one-two and three-electron transfer reactions to O_2_, respectively, during functioning of the electron transport chains (Sharma and Dietz, 2009). The redox active heavy metals such as Cu, Cd Fe, and Zn can induce ROS formation directly by participating in Haber–Weiss and Fenton reactions or indirectly by inhibiting the functioning of enzymes in the cellular antioxidant defense network (Schützendübel and Polle, 2002; Halliwell, 2006; Keunen et al., 2011).

ROS are unstable, highly reactive, and thus, promptly react with other macromolecules to generate more free radicals because unpaired electrons tend to pair and give rise to two stable electron bonds (Foyer and Halliwell, 1976). Being extremely reactive in nature, ROS can interact with macromolecules such as DNA, pigments, proteins, lipids, and other essential cellular molecules depending on the properties like chemical reactivity, redox potential, half-life, and mobility within the cellular system, ultimately leading to a series of destructive processes collectively termed as “oxidative stress” (Mittler, 2002; Sharma and Dietz, 2009; Hossain et al., 2012a,b). Among ROS, ^∙^OH is the most reactive, highly damaging, and short-lived (1 ns), and can oxidize macromolecules within a diffusion distance. Therefore, ROS might induce reversible as well as irreversible modifications in lipids, proteins, and nucleic acids; however, most of these ROS effects are damaging and irreversible.

**Figure 3:**
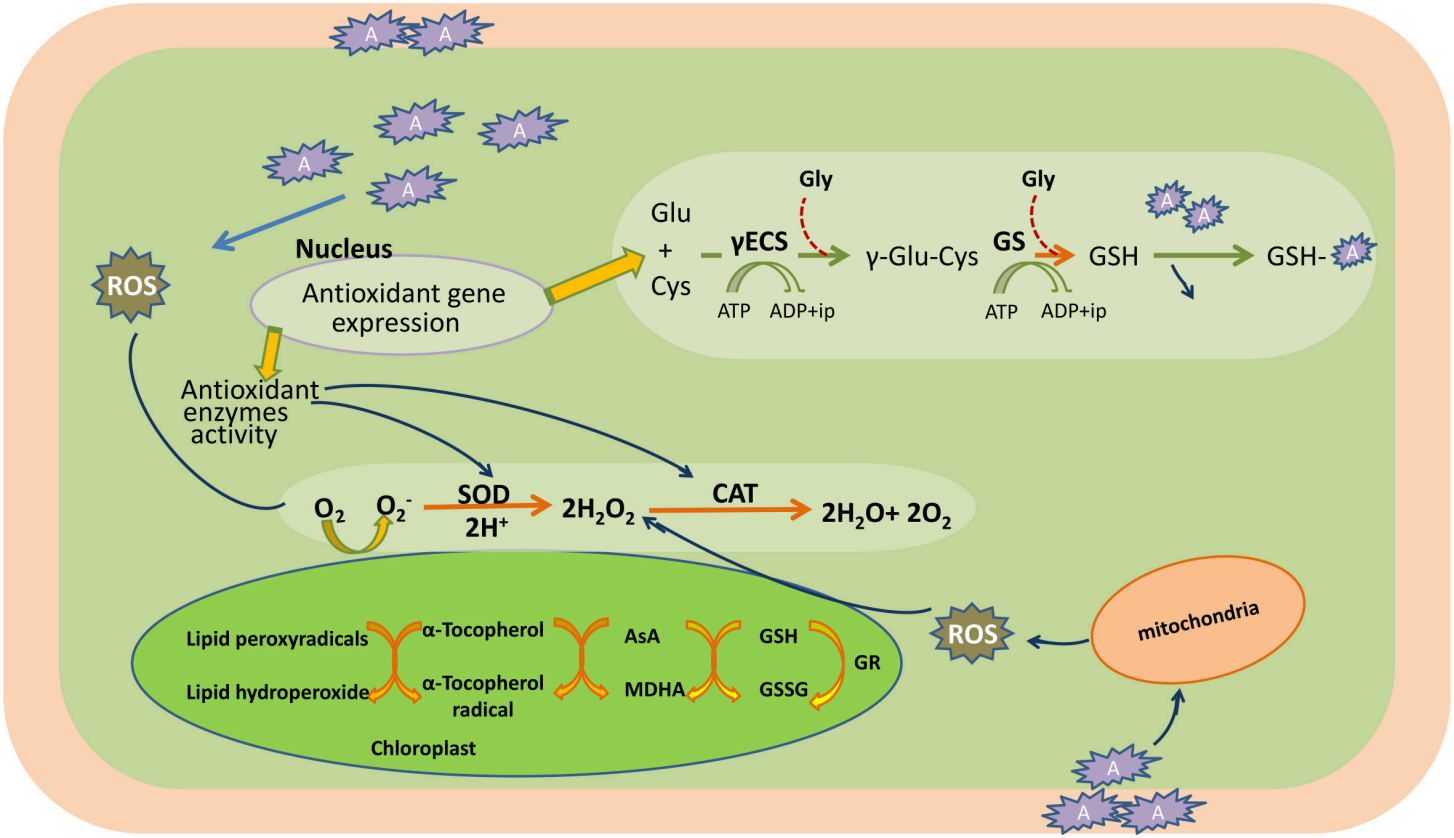
**Heavy metal induced-oxidative stress, tolerance, and detoxification mechanisms in the plant cell**. AsA, ascorbic acid; CAT, catalase; Cys, cysteine; c-ECS, c-glutamylcysteinesynthetase; Glu, glutamine; Gly, glycine; GR, glutathione reductase; GS, glutathione synthetase; GSH, glutathione (reduced); GSSG, oxidized glutathione; H_2_O_2_, hydrogen peroxide; MDHA, monodehydroascorbate; O_2_, oxygen molecule; ${\text{O}}_{2}^{-}$, superoxide radicals; ROS, reactive oxygen species; SOD, superoxide dismutase; A, heavy metal.

Conversely, ROS also act as signaling molecules involved in the regulation of many key physiological processes such as root hair growth, stomatal movement, cell growth, and cell differentiation when finely tuned and regulated by an antioxidative defense system (Foreman et al., 2003; Kwak et al., 2006; Tsukagoshi et al., 2010). It has been shown in several studies that ROS generated by NADPH oxidases during stress are channeled by the plant to serve as a stress signal to activate acclimation and defense mechanisms, which in turn counteract oxidative stress (Mittler et al., 2004; Davletova et al., 2005; Miller et al., 2008, 2010). Therefore, the fate of ROS (i.e., whether it will act as signaling molecule or damaging one) in the cellular system depends upon the output of many complex processes that involve in antioxidative system, signaling cascades, redox alterations, etc. When the generation of ROS exceeds that of the scavenging potential of antioxidants, oxidative stress occurs (Figure 3).

Plants possess a specific mechanism to keep the routinely formed ROS at physiological limit, preventing them from exceeding toxic threshold levels, thereby playing an important role in the acclimation process against an imposed stress (Mittler et al., 2004). This mechanism is known as the plant antioxidant defense system that regulates ROS levels in the cellular system at a particular time (Figure 3). An antioxidant system comprises two types of components: enzymatic and non-enzymatic. The enzymatic antioxidants include superoxide dismutase (SOD; EC 1.15.1.1), catalase (CAT; EC 1.11.1.6), ascorbate peroxidase (APX; EC 1.11.1.11), glutathione reductase (GR; EC 1.6.4.2), monodehydroascorbate reductase (MDHAR; EC 1.6.5.4), dehydroascorbate reductase (DHAR; EC 1.8.5.1), glutathione peroxidase (GPX; EC 1.11.1.9), and glutathione-S-transferase (GST; EC 2.5.1.18), whereas non-enzymatic antioxidants include water-soluble compounds such as ascorbate, glutathione, proline, and α-tocopherol (Apel and Hirt, 2004; Sharma and Dietz, 2009; Hossain et al., 2011, 2012a,b). Although ascorbate and glutathione both function as cofactors of enzymatic antioxidants, both can also directly quench ROS and regulate the gene expression associated with biotic and abiotic stress responses (Hossain et al., 2012a,b). The importance of antioxidants is based on the facts that their increased and/or decreased levels are generally related to an enhanced or declined stress tolerance of stressed plants. Since the evolution of O_2_, antioxidants play an important role in sustaining ROS concentration at an appropriate level that can promote plant development and reinforce resistance to stressors by modulating the expression of a set of genes and redox signaling pathways (Neill et al., 2002). Keeping into consideration the importance of antioxidants in managing ROS levels and oxidative stress, the responses of transgenic plants and/or organisms overexpressing antioxidant and/or its biosynthetic pathway gene(s) against heavy metal stress are listed in Table 1.

**Table 1:** **Summary of transgenic plants over-expressing gene(s) of enzymatic and non-enzymatic antioxidants and their performance against heavy metal stress**.

**Antioxidant and/or its biosynthetic pathway gene(s)**	**Source**	**Target transgenic**	**Response of transgenic plants and/or organisms**	**References**
*CAT3*	*Brassica juncea*	*Nicotiana tabacum*	Cd stress tolerance, better seedling growth, and longer roots	Gichner et al., 2004
*CAT*	*Brassica juncea*	*Nicotiana tabacum*	Zn and Cd stress tolerance, 2.0-fold higher CAT activity than wild type, lower H_2_O_2_ level, and cell death	Guan et al., 2009
*CAT1* and *CAT2*	*Brassica oleracea*	*Arabidopsis*	Low level of H_2_O_2_ and enhanced stress tolerance	Chiang et al., 2013
*Cu/ZnSOD* and/or *CAT*	*Zea mays*	*Brassica campestris*	Less reduction in photosynthetic activity than wild type under SO_2_ stress	Tseng et al., 2007
*MnSOD*	*Triticum aestivum*	*Brassica napus*	SOD activity was 1.5–2.5-fold greater than wild type and enhanced Al tolerance	Basu et al., 2001
*Cu/ZnSOD and APX*	–	*Festuca arundinacea*	Increased tolerance against Cu, Cd, and As due to depressed oxidative stress	Lee et al., 2007
*cytGR/cpGR*	*Bacterial*	*Brassica juncea*	*cpGR* transgenic showed lower Cd accumulation and 50 times higher GR activity than wild type plants	Pilon-Smits et al., 2000
*GR*	*Brassica rapa*	*Escherichia coli*	Increased tolerance against H_2_O_2_ induced by Cd, Zn, and Al due to an enhanced GR activity	Kim et al., 2009
*DHAR/GR/GST*	*Escherichia coli*	*Nicotiana tabacum*	Overexpression enhanced metal tolerance due to maintained redox couples such as ascorbate and glutathione	Le Martret et al., 2011
*DHAR*	*Oryza sativa*	*Escherichia coli*	DHAR-overexpressing *E. coli* strain was more tolerant to oxidant and metal-mediated stress conditions than the control *E. coli* strain	Shin et al., 2008
*MDHAR/DHAR*	*Arabidopsis*	*Nicotiana tabacum*	DHAR but not MDHAR enhanced Al tolerance by maintaining ascorbate level	Yin et al., 2010
*GST*	*Trichoderma virens*	*Nicotiana tabacum*	Enhanced Cd tolerance simultaneously no Cd accumulation, increased activity of SOD, CAT, GST, APX, and GPX than wild type	Dixit et al., 2011
*Sulfite oxidase (SO)*	*Zea mays*	*Nicotiana tabacum*	Increased tolerance against S due to enhanced CAT-mediated H_2_O_2_ scavenging	Xia et al., 2012
*TcPCS1*	*Thlaspi caerulescens*	*Saccharomyces cerevisiae* and *Nicotiana tabacum*	Increased tolerance to Cd due to the decreased lipid peroxidation and enhanced activities of SOD, POD, and CAT	Liu et al., 2011
Serine acetyltransferase	*Thlaspi goesingense*	*Escherichia coli*	Imparts Ni and Co tolerance due to involvement of glutathione	Freeman et al., 2005a
*gshII*	*Escherichia coli*	*Brassica juncea*	Transgenic plants had higher level of glutathione, phytochelatin, and thiols and thus showed enhanced Cd tolerance	Liang Zhu et al., 1999
*AsPCS1/GSH1*	*Allium sativum/Saccharomyces cerevisiae*	*Arabidopsis*	Elevated production of phytochelatin and glutathione that imparts Cd and As tolerance	Guo et al., 2008a
*APS1*	*Arabidopsis*	*Brassica juncea*	Increased Se tolerance due to its rapid reduction	Pilon-Smits et al., 1999
*MTH1745*	*Methanothermobacter thermoautotrophicum*	*Oryza sativa*	Increased Hg tolerance, higher photosynthesis, SOD and POD activity, and lower superoxide radicals, H_2_O_2_, and lipid peroxidation than wild type	Chen et al., 2012b
*PCs*	*Arabidopsis*	*Nicotiana tabacum*	Enhanced Cd tolerance and hampers root-to-shoot Cd transport	Pomponi et al., 2006
*PCS1*	*Arabidopsis*	*Arabidopsis*	Enhanced As tolerance but increased Cd hypersensitivity	Li et al., 2004
*PCS*	*Anabaena* sp. *PCC 7120*	*Escherichia coli*	Enhanced tolerance against multiple stresses such as Cd and Cu by increasing phytochelatin production	Chaurasia et al., 2008
*MT1*	*Mus musculus*	*Nicotiana tabacum*	Enhanced Hg accumulation and tolerance	Ruiz et al., 2011
*MT1*	*Paxillus involutus*	*Hebeloma cylindrosporum*	Increased Cu and Cd tolerance	Bellion et al., 2007
Δ*1-pyrroline-5-carboxylate synthetase*	*Vigna aconitifolia*	*Chlamydomonas reinhardtii*	Transgenic grows rapidly in toxic Cd concentration (100 μM), and bind four-fold more Cd than wild-type cells. Proline likely acts as an antioxidant in Cd-stressed cells and thus increases Cd tolerance	Siripornadulsil et al., 2002
*Alkyl hydroperoxide reductase*	*Anabaena* sp. *PCC 7120*	*Escherichia coli*	Enhanced tolerance against Cu and Cd by enhancing scavenging of H_2_O_2_ and reactive sulfur species	Mishra et al., 2009b

### Plant antioxidant defense system

The term “antioxidant” refers to a class of compounds that protect cells from damage caused by exposure to certain highly reactive species like ROS. The network and coordination of antioxidants are solely responsible for removing, neutralizing, and scavenging ROS. SOD is an enzyme involved in dismutating superoxide radicals generated by oxidation of molecular oxygen into H_2_O_2_ and O_2_ in all the cellular compartments (Fridovich, 1989).

H_2_O_2_ produced by the action of SOD is quite dangerous as it can diffuse through the membrane very easily and damage other cellular components, and thus, metabolites (ascorbate and glutathione) and enzymes (monodehydroascorbate reductase; MDHAR, dehydroascorbate reductase; DHAR and glutathione reductase; GR) are implicated in scavenging of H_2_O_2_(Foyer et al., 1997). Three types of SODs have been reported in plants on the basis of the metal containing (1) the chloroplastic or cytosolic Cu–Zn SOD; the cytosolic Cu–Zn SOD is referred to as Cu–Zn SOD I, whereas the chloroplastic one is referred to as Cu–Zn SOD II; (2) Mitochondrial Mn SOD, and (3) the chloroplastic Fe SOD. APX is regarded as a housekeeping protein in the cytosol and chloroplast, and is involved in scavenging of H_2_O_2_. The substrate for this enzyme is ascorbate and the product, which is a radical, is reduced to dehydroascorbate by an enzyme MDHAR in the presence of an electron donor NADPH (Asada, 1992, 1996). CAT is an important oxidoreductase enzyme that catalyzes decomposition of H_2_O_2_ into H_2_O and O_2_, and it is found in most plants and is localized in the peroxisome. CAT is a key enzyme involved in detoxifying peroxides generated during photorespiration (Morita et al., 1994; Lin and Kao, 2000). Although APX and CAT serves the same function of detoxifying, different affinities (on the basis of Km values) of APX and CAT depict the role of APX in modulating H_2_O_2_ for signaling and CAT in detoxifying excess H_2_O_2_ during stress (Mittler, 2002).

The above mentioned enzymatic components play a relevant role in mitigating heavy metal stress. Several studies have revealed that treatment of heavy metal enhances ROS formation, and thus, substantial increase in the activities of SOD, CAT, and APX was observed (Bharwana et al., 2013; Bashri and Prasad, 2015). A study by Wang et al. (2004) revealed a considerable increase in the activities of POD, APX, and SOD under Cu stress in *B. juncea* seedlings. Similarly, Bharwana et al. (2013) showed that under Pb treatment, there was an appreciable rise in SOD, guaiacol peroxidase, APX and CAT activities, and their activities were further enhanced with the rising concentration of Pb from 50 to 100 μM. Similar to this, Singh et al. (2013) reported increased activity of SOD and CAT under As exposure (5 and 50 μM). These results suggest that cooperative action of antioxidants is required for a detoxification mechanism under heavy metal stress.

## “OMICS” tools

### Metabolomics

Metabolomics refers to the identification and quantification of all low-molecular weight metabolites required by the organisms during developmental stages (Arbona et al., 2013), and some metabolites have been reported to be involved under heavy metal stress tolerance strategies. In the following section, we discuss the role of metabolomics under heavy metal stress.

#### Amino acids and amines

Amino acids and their derivatives have been reported to chelate metal ions, thus conferring metal tolerance to plants. Amino acids, particularly proline and histidine, have been found to chelate metal ions in cells as well as in the xylem sap (Rai, 2002; Sharma and Dietz, 2006). Proline has been reported to accumulate under heavy metal stress (Talanova et al., 2000; Yusuf et al., 2012a). A study on microalgae has demonstrated an increased level of proline under Cd stress (Siripornadulsil et al., 2002). The mechanism of action of increased levels of proline is not sequestration, but it reduces the formation of free radicals and also maintains reducing environment by enhancing the level of GSH (Siripornadulsil et al., 2002). Histidine, another important amino acid, has been found to play an important role under heavy metal stress. Krämer et al. (1996) reported increased histidine levels in the xylem sap of *Alyssum lesbiacum* (Ni hyperaccumulator) under Ni stress. Similarly, Kerkeb and Krämer (2003) reported simultaneous uptake of Ni and Histidine in *B. juncea*. Changes in the histidine content have functional significance in metal stress tolerance (Sharma and Dietz, 2006). NA (aminocarboxylate), an amino acid derivative synthesized by condensation process of three S-adenosyl-L-methionine, has been also reported to chelate metal ions. They have been found to be involved in the movement of mineral nutrients (Stephan and Scholz, 1993). The physiological role of NA has been confirmed by studying the tomato mutant lacking NA synthase, an enzyme involved in catalyzing formation of NA, which showed accumulation of Fe and Cu (Scholz et al., 1985; Herbik et al., 1996). Apart from its chelating action, NA has been reported to be precursor of phytosiderophore mugineic acid involved in binding metals such as Zn, Cu, and Fe (Treeby et al., 1989).

#### Organic acids

Organic acids such as malate, citrate, and oxalate have been reported to transport metals through xylem and are involved in sequestrating ions in vacuole (Rauser, 1999). Citrate, synthesized from citrate synthase, has been shown to have high affinity for Fe, Ni, and Cd, but it is majorly involved in chelating Fe (Cataldo et al., 1988). Malate has been reported to chelate Zn and is mainly involved in chelating cytosolic ions (Mathys, 1977).

#### Glutathione and α-tocopherol

Glutathione (GSH) is a water-soluble tripeptide thiol having low molecular weight (c-Glu-Cys-Gly) and plays a role in the cellular defense against the toxic actions of heavy metals (Meister and Anderson, 1983). Glutathione reductase (GR) readily converts an oxidized glutathione (GSSG) form to reduced form of GSH. GR contains a conserved disulfide bridge that breaks off under metal stress (Creissen et al., 1992; Lee et al., 1998) and plays an important role in defense by reducing GSSG, thus allowing a high GSH/GSSG ratio to be maintained. Studies on *Luffa* seedlings showed an increasing trend in GR activity with an increasing concentration of As (Singh et al., 2015). GR-catalyzed reduction of glutathione disulfide (GSSG) to glutathione (GSH) is NADPH dependent, and to maintain the proper ratio of GSH/GSSG, GSH biosynthesis must be initiated with rapid reduction in GSSG by GR (Kumar et al., 2012).

Alpha-tocopherol is the most active form of vitamin E and is synthesized in the plastids of higher plants. It is found to be involved in scavenging ROS and lipid peroxides (Munne-Bosch, 2005) by quenching ^1^O_2_ in the chloroplast and thus, prevents cell membrane from damage under stress. Several studies have reported changes in the levels of α-tocopherol under heavy metal stress (Collin et al., 2008; Yusuf et al., 2010; Kumar et al., 2012; Lushchak and Semchuk, 2012). A study by Collin et al. (2008) reported an increased concentration of α-tocopherol in *Arabidopsis* under Cd treatment, and the authors suggested that there is an upregulation of genes related to its biosynthesis (Figure 3).

#### Phenols

Synthesis of phenolic compounds under heavy metal stress is due to their high tendency to chelate metals, which is due to the presence of hydroxyl and carboxyl groups that bind to metal ions particularly iron and copper (Jun et al., 2003). Winkel-Shirley (2002) reported induction of phenolic compounds in maize under aluminum exposure. Similarly, Diáz et al. (2001) reported accumulation of phenols in leaves of *P. vulgaris* when exposed to Cu stress. This increase in phenolics is correlated with increased activity of enzymes involved in biosynthesis of phenols under heavy metal stress. Phenols have been reported to be directly involved in chelating Fe ions and thus, suppressing Fenton's reaction, which is the important source of ROS production. Stimulation of CHS (Chalcone synthase) and PAL (phenylalanine ammonia-lyase) activities has been reported in several plants exposed to Cu, Cd, Al, Pb, and Ni (Babu et al., 2003; Sobkowiak and Deckert, 2006; Kováčik and Klejdus, 2008; Kováčik et al., 2009; Pawlak-Sprada et al., 2011). Lavid et al. (2001) reported that tea plants rich in tannin are tolerant to Mn and prevent from Mn toxicity by directly chelating the Mn.

### Ionome and ionomics

Ionome includes the role of mineral nutrients, namely nitrogen (N), phosphorus (P), potassium (K), calcium (Ca), sulfur (S) and magnesium (Mg) and trace metals namely iron (Fe), copper (Cu), manganese (Mn), molybdenum (Mo), cobalt (Co), and zinc (Zn) in alleviating heavy metal toxicity. Although all the mineral nutrients and trace elements are essential for growth and development processes of plants, concentration greater than the required level becomes toxic to the plants. Apart from posing toxicity at higher concentration, nutrients under safe limit play important role in alleviating toxicity induced by heavy metals.

Nitrogen is the most essential nutrient as it is the major constituent of proteins, nucleic acids, vitamins, and hormones. It has the potentiality of alleviating heavy metal toxicity, as it enhances the photosynthetic capacity by increasing chlorophyll synthesis, often synthesizes N-containing metabolites like proline, GSH, etc. and by enhancing the activity of antioxidant enzymes (Sharma and Dietz, 2006; Lin et al., 2011). In a study by Pankovic et al. (2000), it has been shown that supplementing 7.5 mM (optimal level) of N to sunflower reduced the inhibitory effect of Cd on photosynthesis by enhancing Rubisco activity and by increasing protein content. In another study by Zhu et al. (2011), it has been shown that supplementing N fertilizer in the form of 16 mM (NH_4_)_2_SO_4_, alleviated Cd-induced toxicity in *Sedum*. The alleviating potential not only depends on the supplemented level of N but also on the source of N. For instance, when N was applied in the form of ${\text{NH}}_{4}^{+}$-N, it reduced the Cd concentration in leaves of rice plants that was found to be below 100 mg kg^−1^ (Jalloh et al., 2009), but when supplemented as ${\text{NO}}_{3}^{-}$N, it increased the Cd concentration, which suggests antagonistic behavior of ${\text{NH}}_{4}^{+}$- while synergistic of ${\text{NO}}_{3}^{-}$ toward Cd. Another mineral nutrient, phosphorus (P) is the major constituent of cell membrane and nucleic acid, and majorly required for phosphorylation reaction. It has also been reported in alleviating metal-induced toxicity either by diluting the metal or by decreasing the mobility of the metal by forming metal–phosphate complex (Sarwar et al., 2010). In addition, P can also increase GSH content and prevent membrane damage, thereby conferring tolerance to plants (Wang et al., 2009).

Potassium (K) ion is required by the plant to maintain anion–cation balance in cells and plays important regulatory role in protein synthesis and enzyme activation. By improving nutritional status of K, condition of oxidative stress in plants can be minimized (Shen et al., 2000). Supplementation of K at 60 mg kg^−1^ alleviated the toxicity induced by Cd at 25 mg kg^−1^ by increasing the content of AsA and GSH. Similar to nitrogen, K source may also play an important role in alleviating toxicity. A study by Zhao et al. (2004) clearly demonstrated that application of KNO_3_, K_2_SO_4_, and KCl at the rate of 55, 110, and 166 mg.K.kg^−1^, respectively, to the soil has differential effect on Cd (concentration 15 mg Kg^−1^) accumulation. When KCl and K_2_SO_4_ were applied in increasing concentration from 0 to 55 mg kg^−1^, there was 60–90% increase in Cd accumulation in shoots, whereas similar increasing concentrations of KNO_3_ increased the Cd content very marginally, suggesting its protective action against Cd stress.

Sulfur (S), another mineral nutrient, serves as an important constituent of several coenzymes, vitamins, and ferredoxin. Wangeline et al. (2004) reported that Cd toxicity could be alleviated by the upregulation of S-assimilation pathway, thus suggesting toward alleviating role of S under heavy metal toxicity. Studies on *Triticum aestivum* (Khan et al., 2007), *B. juncea* (Wangeline et al., 2004), and *Arabidopsis* (Howarth et al., 2003) have shown increased ATP-sulfurylase (ATPS) and serine acetyl transferase (SAT) activities under Cd stress, and thereby conferring tolerance to these plants. As ATPS activity helps in maintaining GSH level required for regulating Ascorbate (AsA)–GSH cycle (Khan et al., 2009), it has been reported that S at 40 mg.S.Kg^−1^ enhanced the AsA–GSH cycle, thereby reducing Cd-induced toxicity in mustard (Anjum et al., 2008). Thus, indicating toward the possibility that S supplementation to soil system might enhance the formation of S-containing defense compounds such as GSH and phytochelatins. Study by Astolfi et al. (2004) has shown that Cd (100 μM) exposure enhanced the ATPS, *O*-acetyl serine (OAS) thiol lyase activity, which is related to the production of phytochelatins that play the most effective detoxifying mechanism in plants (Zhang et al., 2010). Apart from enhancing the formation of phytochelatins, S also regulates ethylene signaling and thereby helping under heavy metal stress (Masood et al., 2012). Calcium (Ca) is majorly involved in activating the enzymes and also plays an important role in regulating metabolic activities. Due to chemical similarity as well as due to same channels and intracellular Ca-binding sites (Lauer Júnior et al., 2008) of Ca^+2^ and Cd^+2^, Cd present in external medium, replaces the Ca, and thereby affects the growth of plant. However, Ca has been shown to decrease the heavy metal-induced toxicity (Suzuki, 2005; Farzadfar et al., 2013). It has been reported that 30 mM Ca reduced the Cd content from 46.7 to 17.4 μg in *Arabidopsis* seedlings (Suzuki, 2005). Similar to this, Zhenyan et al. (2005) reported enhanced Cd (concentration 0.5 mM) tolerance in *Lactuca sativa* when supplied with 4 mM CaCl_2_, which was due to enhanced expression of phytochelatin synthase. Ca reduces heavy metal-induced toxicity by reducing their uptake, influencing physiological processes, or activating expressions of other defense compounds.

Magnesium (Mg), an important constituent for chlorophyll biosynthesis, plays an essential role under heavy metal toxicity. Abul Kashem and Kawai (2007) reported that Cd (0.25 μM) -induced toxicity in Japanese mustard spinach was alleviated by Mg at 10 mM, and Cd accumulation was reduced by 40%. Mg-induced alleviation is not due to inhibition in uptake but due to enhanced antioxidant enzymes (Chou et al., 2011). Moreover, Mg-induced alleviation has been also correlated with expression of some genes *OsIRT*1, *OsZIP*1, and *OsZIP*3 of rice.

Trace elements are required in lesser amount for the biological system, which include iron (Fe), copper (Cu), manganese (Mn), molybdenum (Mo), cobalt (Co), and zinc (Zn), and their high levels could be toxic. The essentialities of these trace metals are due to their active participation in the redox reactions as well as because of their roles as enzyme cofactors (Sanita di Toppi and Gabbrielli, 1999). However, apart from their roles in biological system, they have been reported to play a crucial role in alleviating metal toxicity. Several trace elements have direct as well as indirect effects on heavy metal availability and toxicity (Sarwar et al., 2010). Direct effects include lowered solubility of heavy metals in the soil (Hart et al., 2005; Shi et al., 2005; Matusik et al., 2008), competition between heavy metals and trace elements for the same membrane transporters (Baszynski et al., 1980; Qiu et al., 2005), and heavy metal sequestration in the vacuoles (Salt and Rauser, 1995; Zaccheo et al., 2006). Indirect effects include dilution of heavy metal concentration by increasing plant biomass (dilution effect) and alleviation of heavy metal stress by increasing antioxidant defense system (Hassan et al., 2005; Suzuki, 2005; Jalloh et al., 2009). Zn, being an important group of metal transporter family, has been suggested to prevent damage caused by Cd toxicity. As reported in the case of *Thalpsi violacea*, plants supplied with 2 mgL^−1^ Cd showed 48.5 mg Kg^−1^ Zn accumulation than that of control (16.8 mgKg^−1^), whereas when the plant was supplemented with 5 mgL^−1^ Cd, Zn accumulation decreased upto 12.8 mg Kg^−1^, suggesting Cd/Zn antagonism (Street et al., 2010). Furthermore, Zn also enhances the activities of antioxidant enzymes and competes with Cd to bind with the membrane protein in order to protect plant (Wu and Zhang, 2002). Other trace metal Fe, under Cd stress, showed reduced Cd uptake and translocation, thus increasing plant growth. Study by Qureshi et al. (2010) revealed that exogenous application of 40 μM Fe reduces the condition of oxidative stress by stabilizing the thylakoid complex under Cd stress. It was also reported that at Fe concentrations of 1.89 mg L^−1^ (moderate) and 16.8 mg L^−1^ (high), under low level of Cd (0.1 mM), plant height showed increment (Nada et al., 2007).

Similarly, studies have also revealed the protective roles of trace elements in ameliorating toxic effects of heavy metals by protecting photosynthetic tissue and increasing antioxidant capacity (Zornoza et al., 2010; Tkalec et al., 2014). Pa1'ove-Balang et al. (2006) have shown that Mn-mediated amelioration of Cd toxicity was associated with a decreased Cd uptake. Apart from the beneficial role played by trace metals, there are some beneficial nutrients like selenium (Se), and silicon (Si) that also play a major role under heavy metal stress. Earlier, selenium (Se) was considered as toxic element but later on Schwarz and Foltz (1957) confirmed it to be an essential one. Studies on lettuce grown under Pb and Cd toxicity supplemented with Se showed a decrease in heavy metal accumulation as well as enhanced uptake of essential nutrients (He et al., 2004). Belokobylsky et al. (2004) and Feng and Wei (2012) have found that Se level up to 5 mg L^−1^ has beneficial effects on *Spirulina platensis* and *P. vittata*, respectively. Filek et al. (2008) have shown that exogenous application of Se alleviates toxic effects of Cd by enhancing the activities of antioxidant enzymes such as SOD, CAT, GPX, and APX. Several reports have revealed that appropriate dose of Se can protect plants against damage by heavy metals such as Hg, Pb, Cd, Cr, and Sb (Khattak et al., 1991; Shanker et al., 1996; Belokobylsky et al., 2004; He et al., 2004; Feng et al., 2011). Role of Si under heavy metal stress is also well established (Singh et al., 2011b; Dragišić Maksimović et al., 2012; Tripathi et al., 2012). Study by Song et al. (2009) has shown that supplementation of Si under Cd stress decreased an uptake and root to shoot translocation of Cd as well as enhanced the activities of enzymes of the defense machinery in *B. chinensis*. Similarly, study by Bharwana et al. (2013) revealed that Si application reduces Pb uptake and enhances the activities of antioxidants *viz*., SOD, GPX, APX, and CAT.

The measurement of elemental composition and their changes as a response to some stimuli in living organisms comes under the study of ionomics. Alteration in ionome could be direct or indirect. Direct one includes the changes in nutrient level in soil or due to impairment of ion transporter, whereas indirect changes might be due to changes in cell wall structure (Salt et al., 2008). Heavy metals due to their interaction with nutrient elements affect the uptake and distribution of these elements and may result in deficiency of minerals thus affecting the growth. Sarwar et al. (2010) suggested that Cd affects the permeability of plasma membrane and thus interferes with the nutrient uptake. However, there exists both antagonistic as well as synergistic interaction between heavy metals and micronutrient uptake, which could be due to differences in plant species and nutrient concentration. Likewise, a study by Cataldo et al. (1983) reported antagonistic interaction between Cd and Fe, Zn, Cu, and Mn in soybean plants, whereas Nan et al. (2002) reported synergistic interaction between Cd and Zn in wheat and corn. In a study by Yang et al. (1998), decreased accumulation of Fe, Mn, and Cu in ryegrass, maize, cabbage, and white clover was observed after Cd exposure, whereas there was increased P accumulation. Similarly, Cui et al. (2008) reported decrease in Fe and Zn uptake in rice after Cd treatment in hydroponic system. A study by Safarzadeh et al. (2013) determined the effect of different doses (0, 45, and 90 mg kg soil) of Cd on uptake of Fe, Zn, Cu, and Mn in seven rice cultivar and reported decrease in Zn, Fe, Mn, and Cu uptake. Not only the uptake decreased but also there was decrease in the translocation of these minerals as Cu and Fe contents found to be greater in roots than in shoots that indicate toward impairment of ions transporters.

Similar to Cd, As has also been reported to influence nutrient uptake and their distribution in plants. Meharg and Hartley-Whitaker (2002) reported As-induced decrease in P uptake is due to chemical similarity between P and arsenate and due to which arsenate enters the plant via the phosphate transport systems. However, the concentration of As also plays an important role in P uptake. Burló et al. (1999) reported higher uptake of P at lower level of As in tomato plants. Similarly, Carbonell et al. (1998) reported increased P uptake in tomato plant when exposed to low level of As. As not only influences P uptake but also affects the uptake of other nutrients like N, P, K, Ca, etc. A study by Carbonell-Barrachina et al. (1997) observed increased concentration of N, P, K, Ca, and Mg in *P. vulgaris* L. plants when exposed to arsenite. Similarly, Carbonell-Barrachina et al. (1994) reported decreased uptake of K, Ca, and Mg (macronutrinets), B, Cu, Mn, and Zn (micronutrinets) in *Lycopersicum esculentum* Mill. The effect of As concentration on nutrient level of hyperaccumulator *P. vittata* L. had also been studied by Tu and Ma (2005), and the authors reported that both micro- and macronutrients were in the range of normal concentration as in non-hyperaccumulators. However, there was enhancement in P and K contents in the fronds of *P. vittata* L. at lower level of As. They reported molar ratio of P/As to be 1.0 in fronds of *P. vittata* L., which is the threshold value for normal growth of plants.

Heavy metal ions such as Cu^+2^, Zn^+2^, Mn^+2^, and Fe^+2^ are essential for plant metabolism but when they are present in excess amount become highly toxic. For instance, Zn and Mn when present in excess impairs growth and compete with Fe. Excess Fe in the plant system participates in the fenton reaction, thereby creating a condition of oxidative stress (Williams and Pittman, 2010; Shanmugam et al., 2011). In order to avoid toxicity induced by mineral elements and trace elements, these are chelated by low molecular weight compounds and sequestrated in vacuoles or excluded to extracellular spaces by transporters situated in the tonoplast or plasma membrane, which plays central role in maintaining metal homeostasis under safe limit. These transporters belong to (1) P_1B_-ATPase or CPx-type ATPase, (2) Cation Diffusion Facilitator (CDF) also known as Metal Tolerance Proteins (MTPs), (3) Natural Resistance-Associated Macrophage Proteins (NRAMPs), and (4) ZRT–IRT-like Protein (ZIP) transporters.

#### P_1B_-ATPases (also known as heavy metal ATPases: HMAs)

P_1B_-ATPases (also known as Heavy Metal ATPases: *HMAs*) are found in a wide range of organisms ranging from prokaryotes to eukaryotes including yeasts, insect, mammals, and plants. Being energized by ATP hydrolysis, they translocate heavy metals (Zn, Co, Cu, Cd, and Pb) out of cytoplasm (to plasma membrane and into vacuole) and thus play important roles in their transport, compartmentalization, and detoxification (Williams et al., 2000; Grennan, 2009). HMA members (HMA2, HMA3, and HMA4) export Zn and Cd. For instance, HMA4 protein that plays a role in nutrition and transport of Zn from root to shoot also protects plants from Cd *via* its efflux (Mills et al., 2005). Hussain et al. (2004) demonstrated that though HMA2 and HMA4 are essential for Zn homeostasis in *Arabidopsis*, double mutants of HMA2 and HMA4 (*hma2* and *hma4*) exhibited increased sensitivity of plant to Cd, suggesting that they may also play a role in Cd detoxification. Similarly, loss of function in HMA2 and HMA4 has been shown to increase Cd sensitivity in *Arabidops*is under phytochelatins deficient, *cad1-3* as well as CAD1 backgrounds (Wong and Cobbett, 2009). A recent study on 349 wild varieties of *A. thaliana* with combined approach of genome-wide association mapping, linkage mapping, and transgenic complementation revealed that HMA3 is solely responsible for variation in amount of Cd accumulated. Varieties with high Cd accumulation indicate toward reduced HMA3 function (Chao et al., 2012). Similarly, Song et al. (2014) found expression of C-type ATP-binding cassette (ABC) transporter (OsABCC) family in *Oryza sativa* and reported its involvement in detoxifying and reducing As accumulation in grains. They reported higher expression of these transporters under higher level of As.

#### Cation diffusion facilitators (CDFs) or metal tolerance proteins (MTPs)

Cation Diffusion Facilitators (CDFs), also known as Metal Tolerance Proteins (MTPs) family, were first reported by Nies and Silver (1995) and found in diverse group of organisms such as bacteria, fungi, animals, and plants. Members of this family are involved in cellular heavy metals homeostasis with principal selectivity for Zn^2+^, Mn^2+^, and Fe^2+^ (Podar et al., 2012). Despite their specificities for Zn^2+^, Mn^2+^, and Fe^2+^, many CDFs may also transport other heavy metals such as Co^2+^, Ni^2+^, and Cd^2+^ (Ricachenevsky et al., 2013). CDFs transporters are involved in heavy metals efflux from the cytoplasm either to the extracellular space or into the organelles (Haney et al., 2005; Peiter et al., 2007; Ricachenevsky et al., 2013). Twelve *MTP* genes have been recognized so far in *A. thaliana* and 10 in *O. sativa* (Gustin et al., 2011). In *A. thaliana*, the first *CDF* gene was characterized as the *Zinc Transporter 1* gene (*ZAT1*) and later renamed as *METAL TOLERANCE PROTEIN 1 (AtMTP1)* (van der Zaal et al., 1999; Delhaize et al., 2003). The *AtMTP1* gene is expressed constitutively in roots as well as in shoots, and when overexpressed in *Arabidopsis*, it enhances Zn tolerance (van der Zaal et al., 1999). However, RNA interference (RNAi)-mediated silencing (Desbrosses-Fonrouge et al., 2005) or T-DNA insertion mutation (Kobae et al., 2004) of this gene increases Zn sensitivity, indicating its important role in regulation of Zn homeostasis. In *A. halleri*, a Zn hyperaccumulator plant, *AhMTP1* gene is believed to have a role in Zn hypertolerance (Shahzad et al., 2010). Unlike *AtMTP1* gene, *AtMTP3* is expressed predominantly in roots and reported to be engaged in maintenance of Zn homeostasis by excluding Zn under Zn oversupply (Arrivault et al., 2006). Another member of MTPs family, *AtMTP11*, has been reported to transport as well as provide Mn tolerance (Delhaize et al., 2007; Peiter et al., 2007). In rice, an ortholog of MTPs, *OsMTP1*, has been characterized and supposed to be located on chromosome 5 and highly expressed in mature leaves and stem (Lan et al., 2012; Yuan et al., 2012).

Menguer et al. (2013) demonstrated that *OsMTP1* gene localized on tonoplast, and when heterologously expressed in the yeast-mutant *zrc1* and *cot1*, complemented Zn hypersensitivity. Besides, its expression also alleviated Co sensitivity, rescued Fe hypersensitivity of the *ccc1* mutant, and restored growth of the Cd-hypersensitive mutant *ycf1*, indicating potential role of this gene in possible biotechnological applications, such as bio fortification and phytoremediation.

#### Natural resistance-associated macrophage proteins (NRAMPs) transporters

Nramp is a highly conserved family of integral membrane proteins that are conserved in different species and located in the plasma membrane of root apical cells (Simões et al., 2012). They are involved in proton-coupled active transport of various heavy metals(Fe^2+^, Zn^2+^, Mn^2+^, Co^2+^, Cd^2+^, Cu^2+^, Ni^2+^, and Pb^2+^) in wide range of organisms including bacteria, fungi, animals, and plants (Hall and Williams, 2003; Cailliatte et al., 2009). However, the physiological role of NRAMP was primarily related with Fe and to a lesser extent for Mn transport (Cailliatte et al., 2009). AtNRAMP1 and 6, forms the first group, and AtNRAMP2–5 constitute the second group (Mäser et al., 2001). Of these, *AtNRAMP1, 3, 4*, and *6* have been shown to encode functional plant heavy metal transporters (Krämer et al., 2007; Cailliatte et al., 2009). Yeast mutants defective in heavy metals uptake have been utilized to investigate transport specificities of plant Nramps. Study revealed that *AtNRAMP1* can complement the *fet3fet4* yeast mutant that is defective in both low and high-affinity Fe transporters, whereas overexpression of *AtNRAMP1* in *Arabidopsis* increases plant resistance to toxic Fe concentrations (Curie et al., 2000). Furthermore, AtNRAMP3 and AtNRAMP4 have been shown to mediate the remobilization of Fe from the vacuolar store and are essential for seed germination under low Fe conditions (Thomine et al., 2003; Lanquar et al., 2005), indicating a role of Nramps in Fe homeostasis.

Since the roles of NRAMPs family proteins were previously related with Fe uptake and transport in biological systems, however, increasing numbers of studies indicated that members of this family have wide range of specificities for pumping cations inside and/or outside the cell. Cailliatte et al. (2010) demonstrated that NRAMP1, localized on plasma membrane, restores the capacity of the *iron-regulated transporter1* (a ZIP family metal transporter) mutant to take up Fe and Co, indicating that NRAMP1 has a broad selectivity for heavy metals *in-vivo*. An *AtNRAMP4* homolog, *TjNRAMP4*, was cloned from the Ni hyperaccumulator *Thlaspi japonicum*, and its expression increased Ni^2+^ sensitivity of wild-type yeast due to elevated Ni accumulation, indicating that this protein might transport Ni into the cytoplasm (Mizuno et al., 2005). Besides regulating uptake and distribution of essential heavy metals, Nramps have also been found to be involved in the transport of non-essential heavy metals. In *Arabidopsis, AtNRAMP3* disruption increases Cd^2+^ resistance, whereas overexpression of this gene confers increased Cd^2+^ sensitivity, indicating that it plays a role in Cd^2+^ transport and sensitivity in plant (Thomine et al., 2000; Mäser et al., 2001).

A *O. sativa* Nrat1 (*OsNrat1*) gene, a Nramp aluminum transporter and localized at all cells of root tips, when expressed in yeast transports only Al^3+^ but not the Mn, Fe, and Cd, indicating that this transporter gene specifically transports only Al (Xia et al., 2011). Furthermore, in knockout of Nrat1, Al sensitivity increased, whereas in wild type, its expression is up-regulated by Al in root that is believed to be required for a prior step of final Al detoxification through sequestration of Al into vacuoles. Study of Cailliatte et al. (2009) demonstrated that *Arabidopsis* transgenic plants overexpressing *AtNRAMP6* gene were hypersensitive to Cd, although plant Cd content remained unchanged, thereby indicating that modification in expression pattern of *AtNRAMP6* affects distribution and availability of Cd within the cell. However, Sano et al. (2012) have shown that *Nicotiana tabacum* NRAMP1 gene (*NtNRAMP1*), a plasma membrane transporter, when overexpressed in tobacco BY-2 cells increases resistance of the cells to both Fe and Cd, and suggested that *NtNRAMP1* moderates Fe-uptake and prevents toxicity resulting from excess Fe or Cd application. Tiwari et al. (2014) also demonstrated that *OsNRAPM1*, localized on plasma membrane of endodermis and pericycle cell, when expressed in *Arabidopsis* provides tolerance against As and Cd with their enhanced accumulation in root and shoot, and proposed that modification in this gene may be helpful in reducing the risk of food chain contamination by these toxic heavy metals. These studies clearly indicate that NRAMP genes are able to encode multi-specific heavy metals transporters. In recent years, a new Nramp5 belonging to rice (*Os* Nramp5) has been characterized, which is responsible for accumulation of Mn in rice and has been reported to encode proteins localized on plasma membrane, thus suggesting that Nramp5 is a major transporter responsible for transport of Mn and Cd (Sasaki et al., 2012). To gain deep insights into the roles of *NRAMP* genes transporter in heavy metals uptake and homeostasis in plants, a more systematic characterization of the different members of the NRAMP family is further required.

#### ZRT, IRT-like proteins (ZIP) transporters

Members of the ZIP family named on the first member identified ZRT IRT- like Protein in Arabidopsis, expressed in roots of iron deficient plants and found to be capable of transporting various heavy metals such as Fe, Zn, Mn, Cd and Ni within cellular systems (Mäser et al., 2001). The key feature of the ZIP family is that these proteins can transport heavy metals from the extracellular space or from organelles lumen into the cytoplasm. In *Arabidopsis*, 15 genes *viz. ZIP1-12, IRT1, IRT2*, and *IRT3* of the ZIP family are reported (Milner et al., 2013). Among these members, AtIRT1, AtIRT2, and AtIRT3 transporters are well characterized, with AtIRT1 being the most studied (Eide et al., 1996; Lin et al., 2009; Vert et al., 2009) for their involvement in regulation of Zn and Fe homeostasis in plants. Rest of the ZIP family members has been studied for their membrane localization and heavy metals they transport into or outside of a specific organelle (Milner et al., 2013). In a model legume *Medicago truncatula*, six ZIP family transporters MtZIP1, MtZIP3, MtZIP4, MtZIP5, MtZIP6, and MtZIP7 have been tested for their ability to complement yeast heavy metals uptake mutants, and each family member was able to rescue the growth of Zn, Mn, and Fe uptake mutants, indicating their function in heavy metals transport (López-Millán et al., 2004).

Apart from the abovementioned transporters, recently another transporter arsenate reductase (ACR) has been characterized in yeast *Saccharomyces cerevisiae*, a model system for As resistance. It was reported that a 4.2-kb region conferred arsenite (AsIII) resistance in *S. cerevisiae*; they found three ACR genes, namely ACR1, ACR2, and ACR3 (Bobrowicz et al., 1997). These authors also reported that ACR1 regulates ACR2 and ACR3 by transcriptional factor and any loss in ACR1 function yeast conferred arsenite and arsenate hypersensitivities (Bobrowicz et al., 1997; Ghosh et al., 1999). Later on, Landrieu et al. (2004a,b) reported that ACR2 represents arsenate reductase that showed homology to yeast ASCR2 (ScACR2). Similarly, Ellis et al. (2006) reported other transporter PvACR2 from *P. vittata* and OsACR2.1 and OsACR2.3 from *O. sativa* (Duan et al., 2007). Earlier, ACR2 (called as CDC25) was thought to be involved in As metabolism in *A. thaliana*. Recent studies on *A. thaliana* have revealed the involvement of new arsenate reductase (ACR), namely HAC1 (Chao et al., 2014) or ATQ1 (Sánchez-Bermejo et al., 2014). Chao et al. (2014) reported that loss of function of HAC1 in *A. thaliana* resulted in decreased As accumulation in roots, and thus, there was diminished As efflux to external medium. Another transporter, OsABCC1 localized in phloem cells of *O. sativa*, has been reported to be involved in sequestration of As to vacuole (Song et al., 2014). However, in anaerobic paddy fields, As (mainly Arsenite) uptake is regulated by transporters of Si, namely Lsi1 (low silicon 1; influx transporter) and Lsi2 (low silicon 2; efflux transporters) (Ma et al., 2008). Apart from these transporters, there are some other transporters as well that transfer arsenate and arsenite. Likewise, a transporter from *P. vittata*, PvACR3 has been reported to compartmentalize As into the vacuoles and loss in its function results in As hypersensitivity (Indriolo et al., 2010).

### Transcriptomics

Investigations on the basic mechanisms of heavy metal tolerance and adaptation are the area of great scientific interest and an intensive research. Various stressors induce an expression of a set of genes in plants (Nakashima et al., 2009).

At molecular level, the regulation of gene expression is very important for the biological processes, which determines the fate of plant development as well as tolerance to heavy metal stress. Stressors trigger large number of genes and several proteins in order to link the signaling pathways that confer stress tolerance (Umezawa et al., 2006; Valliyodan and Nguyen, 2006; Manavalan et al., 2009; Tran et al., 2010). These genes are classified into two groups: the regulatory genes and the functional genes (Tran et al., 2010). The genes of regulatory group encode various transcription factors (TFs), which can regulate various stress-responsive genes cooperatively and/or separately and thus, constitute a gene network. However, the genes of functional group encode metabolic compounds such as amines, alcohols, and sugars, which play a crucial role in heavy metal stress tolerance. The TFs, which are reported to be master regulators, control an expression of gene clusters and usually members of multigene families. Studies reveal that a single TF can control the expression of many target genes *via* specific binding of the TF to the cis-acting element in the promoters of its target genes (Wray et al., 2003; Nakashima et al., 2009). Most of the TFs contain a DNA-binding domain that interacts with cis-regulatory elements in the promoters of its target genes and *via* a protein–protein interaction domain that helps in oligomerization of TFs with other regulators (Wray et al., 2003; Shiu et al., 2005). This type of transcriptional regulatory system is referred as “regulon” (Nakashima et al., 2009). Various TFs families such as AREB/ABF, MYB, AP2/EREBP, WRKY, bHLH, bZIP, MYC, HSF, DREB1/CBF, NAC, HB, ARID, EMF1, CCAAT-HAP2, CCAAT-DR1, CCAAT-HAP3, CCAAT-HAP5, C2H2, C3H, C2C2-Dof, C2C2-YABBY, C2C2-CO-like, C2C2-Gata, E2F-DP, ABI3VP1, ARF, AtSR, CPP, E2F-DP, SBP, MADS, TUB, etc. are known to influence stress response in plants (Singh et al., 2002; Shiu et al., 2005; Shameer et al., 2009). LeDuc et al. (2006), in a transcriptome analysis on plants, reported that plants treated with heavy metals could induce transcription factors that regulate corresponding transcriptional processes.

Liang et al. (2013) reported first FER regulatory gene involved in Fe uptake in tomato, and the functional analog of FER is FER-like Deficiency Induced Transcripition Factor (FIT) that has been conferred to play an important role under Fe deficiency in *Arabidopsis* (Yuan et al., 2005). In addition to this, there are several other subgroups of bHLH family *viz*., AtbHLH38, AtbHLH39, AtbHLH100, and AtbHLH101 that have been shown to be upregulated under Fe deficiency in roots and leaves of *Arabidopsis* (Wang et al., 2007; Yuan et al., 2008). Later, several researchers proposed that AtbHLH38 or AtbHLH39 interacts with FIT and forms heterodimers and directly activates transcription factors for ferric chelate reductase and ferrous transporters, which are the two major genes regulating Fe uptake under deficient condition (Varotto et al., 2002; Vert et al., 2002; Yuan et al., 2008). In *Arabidopsis*, IRT1 has been reported to be the most essential ferrous transporter. Beside transporting Fe, it can also transport Zn, Mn, Co, Ni, and Cd, and thus, these metals get accumulated under Fe deficiency (Vert et al., 2002; Schaaf et al., 2006). A recent study by Wu et al. (2012) in *Arabidopsis* revealed that expression of FIT with AtbHLH38 or AtbHLH39 further activates expression of several other transporters *viz*., HMA3, (MTP3), Iron Regulated Transporter2 (IRT2) that play regulatory role in maintaining Fe content under Cd exposure.

Transcriptome analysis in *A. thaliana* and *B. juncea* exposed to Cd stress has revealed the induction of basic region leucine zipper (bZIP) and zinc finger transcription factors (Ramos et al., 2007). ERF1 and ERF5, two transcription factors belonging to AP2/ERF superfamily (characterized by AP2/ERF domain; Nakano et al., 2006), have been reported to be induced when *A. thaliana* was exposed to Cd (Herbette et al., 2006). Similar induction of TFs has been reported in *A. halleri* under Cd stress (Weber et al., 2006). Differential expression of ERF factors under Cd indicates toward their responses to various levels of Cd stress. A study by Nakashima and Yamaguchi-Shinozaki (2006) reported down-regulation of dehydration-responsive element-binding protein (DREB) transcription factor (involved in cold and osmotic stress responsive genes) in roots of *A. thaliana* under heavy metal treatment and suggested it could be acclimation response and DREB might have helped in normalizing osmotic potential, so that flow of heavy metal-contaminated water could be reduced, thus helping plants to avoid toxic effects of heavy metal. Therefore, acquiring a deep knowledge of the interrelated mechanisms, which regulate the expression of these genes, is a crucial issue in plant biology and necessary to generate genetically improved crop plants for extreme environments like heavy metal stress (Umezawa et al., 2006; Valliyodan and Nguyen, 2006; Nakashima et al., 2009). Summary of an involvement of TFs in conferring heavy metal and other abiotic stresses tolerance is given in Table 2.

**Table 2:** **Summary of transcription factors (TFs) whose overexpression in plants confers heavy metal stress tolerance**.

**Name of TF**	**Family of TF**	**Studied plant**	**Plant response**	**References**
WRKY6	WRKY	*Arabidopsis thaliana*	Plant exhibits dual WRKY-dependent signaling mechanism that modulates As^v^ uptake and transposon expression and provides a coordinated strategy for As^v^ tolerance and transposon gene silencing	Castrillo et al., 2013
WRKY22, WRKY25, and WRKY29	WRKY	*Arabidopsis thaliana*	TFs induced by Cu and Cd involve in stress response *via* MAPK and oxylipin signaling	Opdenakker et al., 2012
WRKY45	WRKY	*Arabidopsis* spp.	Involved in Zn and Fe stress response and homeostasis	van de Mortel et al., 2006
ART1	C2H2	*Oryza sativa*	Constitutively expressed in roots and regulates genes related to Al tolerance and thus increases Al tolerance	Yamaji et al., 2009
ASR5	–	*Oryza sativa*	Overexpression enhanced Al tolerance. Authors suggested that this protein is localized in nucleus and acts as a transcription factor to regulate the expression of different genes that collectively protect rice cells from Al-induced stress	Arenhart et al., 2013
ZIP39	bZIP	*Oryza sativa*	Overexpression regulates endoplasmic reticulum (ER) stress-responsive genes and thus regulates ER stress response	Takahashi et al., 2012
HsfA4a	HSF	*Oryza sativa*	Expression of this TF increases Cd tolerance by inducing up-regulation of MT gene expression	Shim et al., 2009
Hsfs	HSF	*Arabidopsis* spp.	TF up-regulated by Cd stress and plays a role in Cd stress tolerance	Herbette et al., 2006; Weber et al., 2006
CaPF1	AP2/EREBP	*Pinus Virginiana Mill*.	Overexpression of TF enhanced production on of APX, GR, and SOD which confer tolerance against oxidative stress induced by Cd, Cu, and Zn	Tang et al., 2005
OXS2	C2-H2 ZF	*Arabidopsis thaliana*	Enhanced Cd tolerance	Blanvillain et al., 2011
ACEl	–	*Saccharomyces cerevisiae*	TF binds metal-regulatory elements (MREs) upstream promoter of target gene for induction of MT which plays a role in Cu homeostasis	Fürst et al., 1988
ACE1	–	*Arabidopsis thaliana*	Overexpression protects plant against Cu stress by inducing activity of SOD and POD, and suppressing inhibition in chlorophyll biosynthesis	Xu et al., 2009
ACE1	–	*Saccharomyces cerevisiae*	TF binds MREs upstream promoter of target gene for induction of MT which plays a role in Cu homeostasis	Dixon et al., 1996
ACP1	AP2/EREBP	Physcomitrella patens	Expression of this gene enhances metal responding genes which confer tolerance against Cd and Cu	Cho et al., 2007
OSISAP1	Zinc-finger protein	*Nicotiana tabacum*	Overexpression enhances tolerance against various abiotic stresses including heavy metal like Cu, Cd, Mn, Ca, Zn, and Li	Mukhopadhyay et al., 2004
STOP1	C2-H2 ZF	*Arabidopsis thaliana*	Expression protects plants from Al toxicity by proton pump regulation	Iuchi et al., 2007
bHLH38 and bHLH39	bHLH	*Arabidopsis thaliana*	Overexpression enhanced Cd tolerance by increased Cd sequestration in roots and also improved Fe homeostasis in shoots	Wu et al., 2012
bHLH100	bHLH	*Arabidopsis* spp.	Involved in Zn and Fe stress response and homeostasis	van de Mortel et al., 2006
–	MYB, bHLH, bZIP	*Sedum alfredii*	These TFs families were up-regulated by Cd and involved in Cd hyperaccumulation and tolerance	Gao et al., 2013
PYE	bHLH	*Arabidopsis thaliana*	Expression is implicated in regulating plant growth response against Fe deficiency	Long et al., 2010

#### Mitogen-activated protein kinase MAPK cascade

MAPK cascade are activated in response by plants when exposed to heavy metal stresses. This cascade has its significance in activation of signal transduction pathway used in hormone synthesis (Jonak et al., 2002). This cascade involves three kinases *viz*., MAPK kinase kinase (MAPKKK), the MAPK kinase (MAPKK), and the MAPK, which are activated by phosphorylation process. The finally formed phosphorylated MAPK cascade phosphorylates substrates in cells including transcription factors in nucleus. Therefore, MAPK regulates the transduction of information downstream. Jonak et al. (2004) have shown four isoforms of MAPK that are activated under Cu or Cd stress in *Medicago sativa*. All these pathways finally lead to regulation of transcription factors that in turn activate genes for activation of metal transporters, biosynthesis of chelating compounds, and other defensing compounds.

### Proteomics

Proteomics is a well-established technique in the post-genomic era (Liu et al., 2013). Proteomics deals with the study of large-scale expression of proteins in an organism encoded by its genome (Anderson and Anderson, 1998). Proteomics not only serves as a powerful tool for describing complete protein changes in any organisms but it can also be used to compare variation in protein profiles at organ, tissue, cell and organelle levels under various stress conditions including heavy metal stress (Ahsan et al., 2009). Although genomic analysis has enhanced our understanding regarding plants' response to heavy metal toxicity, transcriptomic changes in the genome are not always reflected at protein level (Gygi et al., 1999; Hossain and Komatsu, 2013). For instance, putative Zn and Mg transporter protein MHX was more abundant in *Arabidopsis* even though its corresponding transcript level was not different (Elbaz et al., 2006). This suggests that transcription of any gene is not a guaranty that gene would be translated into a functional protein. This may occur due to the potential impact of post-transcriptional and translational modifications, protein folding, stability and localization, protein–protein interactions, which are considered important determinants of a protein function (Dalcorso et al., 2013b). Therefore, depth analyses of proteomics offer a new platform for identifying target proteins, which take part in heavy metal detoxification, and in studying complex biological processes and interactions among the possible pathways that involve a network of proteins (Ahsan et al., 2009).

Furthermore, it is known that proteins directly take part in plant stress responses, and plant adaptations to heavy metal stress are always accompanied with deep proteomic changes. Therefore, technique of proteomics can be exploited for deciphering the possible relationships between proteins abundance and plant stress adaptation. It can contribute to better understanding of physiological mechanisms under heavy metal stress such as perception of stress and further signaling cascade that leads to changes in the expression of huge numbers of genes at transcriptional level and in metabolite profile, which could be used for an acquisition of an enhanced plant tolerance under heavy metal toxicity (Kosová et al., 2011). Studies have revealed that an abundance of defense proteins was increased for scavenging of ROS, and molecular chaperones play a role in re-establishing the conformation of a functional protein that contributes in helping heavy metal stressed plants to maintain the redox homeostasis (Zhao et al., 2011; Sharmin et al., 2012; Wang et al., 2012). Under heavy metal stress, modulations of various metabolic pathways occur such as photosynthesis, respiration, nitrogen metabolism, sulfur metabolism, etc. particularly in photosynthesis and mitochondrial respiration that help stressed plants to produce more reducing power such as NADPH, NADH, and FADH_2_ and assimilatory power ATP to compensate high energy demand of heavy metal-challenged plants (Hossain and Komatsu, 2013). For example, an increased abundance of RUBISCO large sub unit (LSU)-binding proteins, oxygen-evolving enhancer protein 1 and 2, NAD(P)H-dependent oxido-reductase, and photosystem I and II-related proteins is an adaptive feature to withstand heavy metal stress (Semane et al., 2010). The cellular mechanism of stress sensing and further transduction of signals into the cell appear to be the first reactions in the plant cell against heavy metal. Furthermore, an intracellular communication of stress signals plays a fundamental role in signal transduction pathways under stress, which ultimately activate defense-related genes and thus signaling cascades (Hossain et al., 2012c). Therefore, to decipher an underlying molecular mechanism of alterations in the protein signature of a plant cell in order to withstand stress, a deep study on the cellular as well as organelle proteomics would be of great importance in developing heavy metal-tolerant crops. Alterations in protein profile under heavy metal stress, which could be utilized for developing heavy metal-tolerant plants, are given in Table 3.

**Table 3:** **Summary of heavy metal-induced changes in protein expressions and their potential uses in developing heavy metal tolerant plants**.

**Metal**	**Technique(s) used**	**Plant species**	**Alterations in protein(s) expression profile**	**Plant response**	**References**
Cd	2DE, MALDI-TOF-MS, LC–ESI-QTOF-MS	*Arabidopsis thaliana*	~1100 Spots reported, 41 spots showed significant changes including phytochelatins, glutathione-S-transferases, ATP sulfurylase, glycine hydroxymethyl transferase, trehalose-6-phosphate phosphatase	Alterations in these proteins in plant roots help to withstand Cd stress *via* modulating S assimilation	Roth et al., 2006
	2DE, MALDI-TOF/TOF-MS	*Phytolacca americana*	32 Proteins are differentially expressed, 14 enhanced, and 11 reduced under Cd treatment. Major changes were in photosynthetic pathway, S and GSH metabolism, transcription, translation and chaperones, 2 cys-peroxiadse and oxido-reductases proteins	These alterations play a key role in enhancing Cd hypertolerance in plant	Zhao et al., 2011
	2DE, MALDI-TOF/TOF-MS	*Arabis paniculata*	18 Proteins differentially expressed upon Cd treatment which were mainly related with photosynthetic pathway and antioxidant defense system such as ribulose-5-phosphate 3-epimerase (RPE), RuBisCO activase, Protein thylakoid formation 1 (THF1), Mn-SOD, APX, GST	Plant adopted alterations mainly in antioxidative/xenobiotic defense and hence exhibited increased Cd tolerance	Zheng et al., 2011
	2-D DIGE, MALDI-TOF/TOF	*Populus* sp.	A number of changes in the expression of proteins with various functions were identified; in particular a decreased abundance of oxidative stress regulating proteins, whereas pathogenesis-related proteins showed a drastic increase in abundance. Furthermore, a large number of proteins involved in carbon metabolism showed a decrease in abundance, while proteins involved in remobilizing carbon from other energy sources were up-regulated	Due to deep proteomic changes, plant experienced lesser negative impact of Cd on physiological parameters and hence plant showed Cd tolerance	Kieffer et al., 2008
	2DE, MALDI-TOF-MS	*Oryza sativa*	36 Proteins either up-and/or down-regulated by Cd treatment. Most of the proteins were related to oxidative stress and antioxidative system	Antioxidative system related proteins play a role in Cd tolerance	Lee et al., 2010
	2DE	*Thlaspi caerulescens*	48 Tentatively spots identified which represent core metabolic functions, e.g., photosynthesis, nitrogen assimilation, carbohydrate metabolism as well as putative signaling and regulatory functions	The possible roles of some of the proteins were related with metal accumulation and tolerance	Tuomainen et al., 2006
As	2DE, MALDI-TOF-MS	*Oryza sativa*	23 Proteins up-regulated related with defense proteins like S-adenosylmethionine synthetase (SAMS), GSTs, cysteine synthase (CS), GST-tau, and tyrosine-specific protein phosphatase proteins (TSPP), and an omega domain containing GST	SAMS, CS, GSTs, and GR presumably work synchronously and GSH plays a key role in protecting rice roots against As stress	Ahsan et al., 2008
	IPG, 2-DE, MALDI-TOFMS, ESI-MS/MS	*Oryza sativa*	12 Proteins differentially expressed related with energy production and metabolism. RuBisCO large subunit and chloroplast 29 kDa ribonucleoproteins were decreased	Reduction in photosynthetic machinery proteins was related with As toxicity	Ahsan et al., 2010
	2DE, MALDI-TOF-MS, LC-MS/MS	*Chlamydomonas reinhardtii*	15 Proteins overexpressed like oxygen-evolving enhancer protein, rubisco small subunit 1, chaperones, Fe-SOD, Mn-SOD, and heat shock like proteins	Organism exhibited time course acclimation against As stress by modulating protein signatures	Walliwalagedara et al., 2012
Hg	2-DE, MALDI-TOF-TOF-MS	*Suaeda salsa*	43 Proteins with significant changes reported. They include proteins related to metabolic processes, photosynthesis, stress response, protein fate, energy metabolism, signaling pathways, and immunosuppression	Alterations in these proteins was linked with Hg toxicity	Liu et al., 2013
	2DE, ESI-MS/MS	*Oryza sativa*	25 Proteins differentially expressed by Hg involved in cellular functions including the redox and hormone homeostasis, chaperone activity, metabolism, and transcription regulation	Plant exhibited Hg toxicity due to alterations in these proteins	Chen et al., 2012a
	2DE	*Oryza sativa*	33 Proteins were highly reproducible. Most of the proteins showed homology to RuBisCO protein, and some to defense/stress-related proteins, like the pathogenesis related class 5 protein (OsPR5), the probenazole-inducible protein (referred to as the OsPR10), SOD, and the oxygen evolving protein	Severe fragmentation of ribulose-1,5-bisphosphate carboxylase/oxygenase and induction of stress-related proteins causes Hg toxicity	Hajduch et al., 2001
Cr	2DE, MALDI-TOF, MALDI-TOF-TOF	*Miscanthus sinensis*	36 Proteins differentially expressed. The identified proteins included: heavy metal-inducible proteins such as carbohydrate and nitrogen metabolism, molecular chaperone proteins, and novel proteins such as inositol monophosphatase, nitrate reductase, adenine phosphoribosyl transferase, formate dehydrogenase, and a putative dihydrolipoamide dehydrogenase	*Miscanthus* plant experienced Cr toxicity due to altered vacuole Cr sequestration, nitrogen metabolism, and lipid peroxidation in roots	Sharmin et al., 2012
	2DE, MALDI-TOF-MS-MS	*Zea mays*	58 Proteins identified related with photosynthesis and chloroplast organization, the redox homeostasis and defense response, RNA processing, protein synthesis and folding, DNA damage response, mitochondrial oxidative phosphorylation, and miscellaneous with unknown function	Plant exhibited Cr toxicity due to the deep changes in proteomics	Wang et al., 2013
Cu	2DE	*Oryza sativa*	Changes RuBisCO, defense/stress-related proteins, like the pathogenesis related class 5 protein (OsPR5), the probenazole-inducible protein (referred to as the OsPR10), and SOD	Alterations in these protein resulting in Cu stress	Hajduch et al., 2001
	SDS-PAGE and 2DE	*Oryza sativa*	13 Proteins identified such as metallothionein-like protein, membrane-associated protein-like protein, putative wall-associated protein kinase, pathogenesis-related proteins, and the putative small GTP-binding protein Rab2 which were up regulated by Cu stress. Three proteins, a putative small cytochrome P450 (CYP90D2), a putative thioredoxin and a putative GTPase, were down regulated by Cu stress	Plant experienced Cu toxicity due to a decline in thioredoxin and CYP90D2 and thus engineering of this protein may enhance Cu tolerance	Zhang et al., 2009a,b
	2DE-MS	*Populus* sp.	450 Proteins were reproducibly separated, including metabolic processes proteins such as photosynthesis, S assimilation, sugar metabolism, chaperones, and defense related proteins such as GST, DHAR, APX	Plant adjusts its metabolism against Cu stress by changing protein expression. These proteomic temporal features should be taken into account for the future development of metal tolerant plants	Lingua et al., 2012
	IPG, 2-DE, MALDI-TOF-MS	*Ectocarpus siliculosus*	Up-regulation of photosynthesis (PSII Mn-stabilizing protein of OEC33), glycolysis, and pentose phosphate metabolism; higher accumulation of HSP70 and vBPO	Cu stress leads to up-regulation of certain proteins such as HSP70 and vBPO for proper protein folding and ROS detoxification, respectively	Ritter et al., 2010
	IPG, 2-DE, LC-MS/MS	*Cannabis sativa*	Induced aldo/keto reductase, PCs expression, suppression/no change in ROS scavenging enzymes	Cu induced aldo/keto reductase acts as a Cu chaperone reduce Cu ions to Cu(I), promote PCs-mediated vacuolar transport in order to reduce Cu toxicity	Bona et al., 2007
	SDS-PAGE and 2DE	*Oryza sativa*	25 Protein spots were differentially expressed in Cu-treated samples. Among them, 18 protein spots were up-regulated, and 7 protein spots were down-regulated. Antioxidants proteins such as glyoxalase I, peroxiredoxin, aldose reductase, and DnaK-type molecular chaperone up-regulated. Moreover, down-regulation of key metabolic enzymes like alpha-amylase or enolase revealed also observed	Plant showed physiological alterations under Cu stress due to the change in metabolic pathway related proteins	Ahsan et al., 2007
Zn	iTRAQ	*Arabidopsis thaliana*	521 Proteins identified. Among them, several were membrane proteins. IRT1, an iron and zinc transporter, and FRO2, a ferric-chelate reductase, increased greatly in response to excess Zn	Plant exhibits Zn stress in which V-ATPase activity might play a central role	Fukao et al., 2011
	2DE-MS	*Populus* sp.	450 Proteins were reproducibly separated, including metabolic processes proteins such as photosynthesis, S assimilation, sugar metabolism, chaperones, and defense related proteins such as GST, DHAR, APX	Plant adjusts its metabolism against Zn stress by changing protein expression	Lingua et al., 2012
Ni	2DE, MALDI-TOF-MS	*Brassica juncea*	61 Proteins differentially expressed. The majority of proteins were found to be involved in S metabolism and protection against oxidative stress. The induced expression of photosynthesis and ATP generation-related proteins were also observed	An increased expression of defense proteins and those related with energy metabolism suggesting the Ni tolerance in plant is an energy-demanding process	Wang et al., 2012
	2-DE, LC-MS/MS	*Alyssum lesbiacum*	12 Proteins differentially expressed. They include proteins of S metabolism, antioxidants, heat shock	Modulation in S metabolic and defense related proteins enhanced Ni tolerance of plant	Ingle et al., 2005
Mn	IPG, 2-DE, Nano-LC-MS/MS, ESI MS/MS	*Vigna unguiculata*	8 Differentially expressed proteins indentified involved in CO_2_ fixation, stabilization of the Mn cluster of the photosystem II, pathogenesis-response reactions, and protein degradation	Coordinated interplay of apoplastic and symplastic reactions help plant to withstand Mn toxicity	Führs et al., 2008
	2DIEF/SDS-PAGE, 2D Blue native BN/SDS-PAGE	*Hordeum vulgare*	A range of proteins differentially expressed in response to Mn. A putative inorganic pyrophosphatase, a probenazole-inducible protein (PBZ1), a protein belonging to a universal stress protein (Usp) family, a chloroplast translational elongation factor (Tu) and the 50S ribosomal protein L11	In young leaves toxicity resulted due to Mn-induced Mg and Fe deficiencies	Führs et al., 2010

Apart from inducing synthesis of amino acids (proline and histidine), amines, organic acids, and plant antioxidant α-tocopherol and glutathione, some nitrogen containing metabolites like some peptides (phytochelatins, metallothioneins, and ferritins) have been reported to play an important role under heavy metal stress. In the following section, we will discuss about the roles of peptides in heavy metal tolerance.

## Peptides

Phytochelatins (PCs) have been the best-characterized chelators in plant systems. PCs belong to a family of metal-binding protein having general structure (c-Glu-Cys)*n*Gly (*n* = 2–11) (Cobbett and Goldsbrough, 2002). These are synthesized by the transpeptidation of the γ-Glu-Cys moiety of GSH, and the transpeptidation reactions are carried out by enzyme named phytochelatin synthase (PCS). It has been reported that PCS were activated under heavy metal exposure (Rauser, 1995; Cobbett, 2000), and similar PC synthase activity has been observed in several other crops (Klapheck et al., 1995; Chen et al., 1997; Mishra et al., 2009a). Loeffler et al. (1989) confirmed that metals induce PC synthesis, in *in-vivo* as well as in *in-vitro* cultures, and were continuously synthesized until activated metal ions chelated. Haag-Kerwer et al. (1999) reported induction of PCs in *B. juncea* after the accumulation of Cd in the cells, and thus plays important role in detoxifying heavy metals (Hirata et al., 2005). Due to the presence of thiol group, they have the capability of chelating metals and forming complexes (Cobbett, 2000), which are then sequestrated in the vacuole. From the preceding discussion, it is clear that chelation by PCs is not a simple process but involves a complex molecular mechanism, where firstly, the PCS gets activated by metal ion and biosynthesis of PCs takes place; secondly, formation of complexes and sequestration in vacuole; thirdly, more complexation with the sulfides or organic acids in the vacuole, and finally detoxified. Besides detoxifying heavy metals, PCs also play a major role in metal ion homeostasis and thus regulating the metal ion availability in plant cells (Guo et al., 2008b).

Like PCs, metallothioneins (MTs) are synthesized and activated under heavy metal toxicity. They belong to a family of low molecular weight protein having cysteine-rich metal binding peptide. Due to the presence of mercaptides, they have the ability of binding metal ions. Metal-binding activities of MTs have been expressed in *Escherichia coli* in presence of Cd, Zn, and Cu (Tommey et al., 1991). In addition, Zhou and Goldsbrough (1994) reported restoration in Cu tolerance ability of MT-deficient yeast strains, when provided with the *Arabidopsis* MTs. Similar to this, Zhigang et al. (2006) conferred increased tolerance of *A. thaliana* to Cd and Cu, when ectopically substituted with *B. juncea* MT. Moreover, comparative study of mutant and wild-type *A. thaliana* has clearly revealed that MT mutant was hypersensitive to Cd and accumulated much lower amount of Cd than wild type, thus conferring role of MTs in both heavy metal tolerance as well as accumulation (Zimeri et al., 2005). In terms of transcript amount, expression of MT genes varies during different developmental stages of plant as well as under varying environmental condition (Rauser, 1999). Beside, chelating metal ions MTs can also catalyze antioxidant protection mechanism as well as plasma membrane repair (Hamer, 1986).

Ferritins are other multimeric proteins that could accumulate iron atom (Harrison and Arosio, 1996). However, animal ferritins have been reported to store other metals like Cu, Zn, Cd, etc., whereas plants ferritin could store only Fe. These are synthesized in plants when there is excess Fe in the surroundings and thus represents first-line defense against Fe-induced oxidative stress (Ravet et al., 2009). These are not only involved in storing or releasing Fe but also involved in scavenging free reactive iron (Ravet et al., 2009).

## Plant growth hormones

In spite of five classical plant hormones, i.e., gibberellins (GAs), cytokinins (CKs), auxins, abscisic acid (ABA), and ethylene, jasmonate (JA), brassinosteroids (BR), and salicylic acid (SA) are also well known for regulating many physiological processes and heavy metal stress tolerance (Freeman et al., 2005b; Gangwar et al., 2010; Gangwar and Singh, 2011; Peleg and Blumwald, 2011; Choudhary et al., 2012a,b; Vriet et al., 2012). Furthermore, it is also expected that some more growth hormones are yet to be discovered in future. In laboratory as well as filed studies, two strategies have been used for plant hormone-mediated increase in stress tolerance as well as crop yield. These strategies include exogenous application of plant hormones and genetic manipulation of their endogenous contents. Both approaches have given promising results for increasing crop yield and enhancing stress tolerance in a variety of crop species (Vriet et al., 2012). Although SA and GAs both are cost effective and can easily be availed for their exogenous application in crop fields under stress conditions, high cost of synthetic BRs and the variability of the results have discouraged the use of exogenous BRs in agriculture and horticulture (Khripach et al., 2000; Gomes, 2011). In this context, modulation of endogenous BRs levels by genetic engineering has emerged an efficient strategy for enhancing crop yield under normal as well as adverse growth conditions (Divi and Krishna, 2009). Herein, we have summarized recent advances made in enhancing heavy metal tolerance as well as achieving high yield with desired agronomic traits by using salicylic acid (SA), brassinosteroids (BRs), and gibberellins (GA).

### Salicylic acid (SA)

In recent years, SA has gained much scientific attention due to its function as an endogenous signaling molecule conveying local and systemic plant–pathogen defense responses. Besides this, it has been reported that SA also plays a role in plant response against abiotic stresses such as heavy metal toxicities, chilling, drought, osmotic stress, and heat. In this sense, SA appears to be an “effective therapeutic agent” for plants as in the case of mammals (Rivas-San Vicente and Plasencia, 2011). Salicylic acid is a phenolic compound biosynthesized in all the plant kingdoms through the phenylpropanoid pathway (Métraux, 2002).

Being well characterized and studied role of SA in pathogen resistance, an exogenous application of SA could also provide protection against several types of abiotic stresses such as heavy metals, high or low temperature, salinity, radiation, etc. (Horváth et al., 2007; Hayat et al., 2010). Since under stress condition, reduced plant growth could result from an altered hormonal status, and thus, an exogenous application of plant hormones like SA has been an attractive approach to attenuate heavy metal stress. Studies carried out so far demonstrated that SA treatment to plants evoke acclimatization effect, which causes an enhanced tolerance toward heavy metal stress primarily due to the adjustment of metabolic processes such as enhanced antioxidative capacity. In one of the first works, it was demonstrated that SA may induce protective effects against Cu toxicity in tobacco and cucumber (Strobel and Kuc, 1995). Later, an increasing numbers of studies have demonstrated SA-mediated amelioration of toxicities produced by various heavy metals. Zhou et al. (2009) reported that 0.2 mM of SA ameliorates Hg toxicity in alfalfa by increasing activity of APX, POD, and NADPH oxidase, and amounts of ascorbate, glutathione, and proline, and decreased lipid peroxidation, and an increase in NADPH oxidase activity. It indicates a role of ROS signaling in such an amelioration process.

In maize plant, Cd declined the growth by inhibiting chlorophyll synthesis, ribulose 1,5-bisphosphate carboxylase and phosphoenolpyruvate carboxylase, and enhancing oxidative damage such as lipid peroxidation and electrolyte leakage, whereas SA pretreatment of seeds reversed these toxic effects (Krantev et al., 2008). In cucumber, an exogenous application of SA has also been reported to enhance Mn tolerance by modulating nutrients' statuses and antioxidant defense system (Shi and Zhu, 2008). Similarly, in pea seedlings, Cd toxicity caused decline in growth due to an inhabited photosynthetic process and enhanced oxidative damage, whereas SA pretreatment alleviated damaging consequences of Cd on growth and photosynthesis (Popova et al., 2009). Moreover, Guo et al. (2009) have demonstrated that SA pretreatment alleviated Cd toxicity in rice by enhancing antioxidant components such as SOD, CAT, POD, glutathione, and non-protein thiols, which in turn depressed oxidative damage induced by Cd. Conversely, Metwally et al. (2003) reported that SA down-regulates activities of antioxidant enzymes such as CAT and APX under Cd stress and concluded that SA alleviates Cd toxicity not at the level of antioxidant defense system but by affecting other mechanisms of Cd detoxification. Contrary to this, SA at higher concentration may also cause tissue damage and cell death by inducing oxidative stress (Horváth et al., 2007). For instance, SA has been shown to potentiate generation of ROS in photosynthetic tissue under abiotic stresses and thus causes tissue damage (Borsani et al., 2001). Therefore, it can be concluded that the concentration of SA appears to be important in regulating stress responses. The SA-mediated alterations in genes that are involved in mediating stress tolerance are listed in Table 4.

**Table 4:** **Summary of plant hormone-mediated alterations in genes and their relation with an increased heavy metal stress tolerance**.

**Plant hormone**	**Alteration in gene(s)**	**Studied plant**	**Response**	**References**
Salicylic acid	Heam oxygenase-1 (*HO-1*)	*Medicago sativa*	Alleviation of Cd-triggered oxidative stress by re-establishing redox homeostasis	Cui et al., 2012b
	Serine acetyltransferase	*Thlaspi* spp.	Elevated level of glutathione and increased Ni tolerance	Freeman et al., 2005b
	Citrate synthase	*Cassia tora*	Enhanced Al tolerance through an efflux of citrate	Yang et al., 2003
	*SR3*	*Phaseolus vulgaris*	This gene up-regulated by SA and provides resistance against Hg, Cd, As, and Cu	Zhang et al., 2006
	*gsh1, gsh2, or gr1* and *gst*	*Arabidopsis thaliana*	SA did not influence expression of these genes except *gst* and thus did not affect Cu and Cd tolerance	Xiang and Oliver, 1998
	*MT1* and *MT2*	*Arabidopsis thaliana*	SA did not alter expression of these genes hence did not impart Cu tolerance	Murphy and Taiz, 1995
Brassinosteroids	Antioxidant defense related genes	*Raphanus sativus*	Increased resistance against Cr toxicity due to diminished production of ROS and an enhanced defense system	Choudhary et al., 2012b
	*Fe-SOD, CAT1, APX, GST1, GR, POD, GSH1, PAL, PPO, SKDH*, and *CAD*	*Solanum lycopersicum*	Alleviates Cd-induced inhibition on photosynthesis by up-regulating defense system and decreasing oxidative stress	Ahammed et al., 2013
	Genes encoding polyamines, IAA and ABA metabolic genes, and Cu homeostasis	*Raphanus sativus*	Lower ion leakage due to a maintenance of Cu homeostasis and hence an enhanced Cu tolerance	Choudhary et al., 2012a
	*HSP83, HAT2, GH3.9, SAL2, NIA1, GAS4, SAUR36, DWARF1, DWARF4*, and *BR6OX*	*Arabidopsis thaliana*	BR-exhibited synergistic effect with Cd and increased Cd sensitivity of plants	Villiers et al., 2012
	NADPH oxidase and *RBOH, MAPK1*, and *MAPK3*	*Cucumis sativus*	BR-mediated production of H_2_O_2_*via* NADPH oxidase increased stress tolerance in cooperation with kinases	Xia et al., 2009
	Induced NO production that up-regulates ABA biosynthetic gene *vp14*	*Zea mays*	BR-induced NO production that up-regulates ABA biosynthesis gene *vp14* and thus confers stress tolerance	Zhang et al., 2011
	Set of stress marker genes	*Brassica napus*	Increased tolerance against abiotic stresses such as drought and cold	Kagale et al., 2007
	*UBC32*, a stress-induced functional ubiquitin conjugation enzyme (E2)	*Arabidopsis thaliana*	Protects plants from abiotic stress through endoplasmic reticulum (ER)-associated protein degradation (ERAD) component and UBC32 plays a crucial role in such protection	Cui et al., 2012a
Gibberellic acid	*IRT1*	*Arabidopsis thaliana*	GA-suppressed up-regulation of *IRT1* and enhanced accumulation of NO that enhanced Cd tolerance	Zhu et al., 2012
	*CAX2*	*Nicotiana tabacum*	GA did not influence expression of this gene and did not alter Mn and Cd tolerance	Hirschi et al., 2000
	GA-biosynthesis and redox genes	*Glycine max*	Increased Cu tolerance due to decreased oxidative damage and enhanced antioxidant defense system	Khan and Lee, 2013
	adenosine 5′-phosphosulfate reductase (APR)	*Arabidopsis thaliana*	GA plays a role in abiotic stress tolerance *via* regulating S assimilation pathway	Koprivova et al., 2008

**Figure 4:**
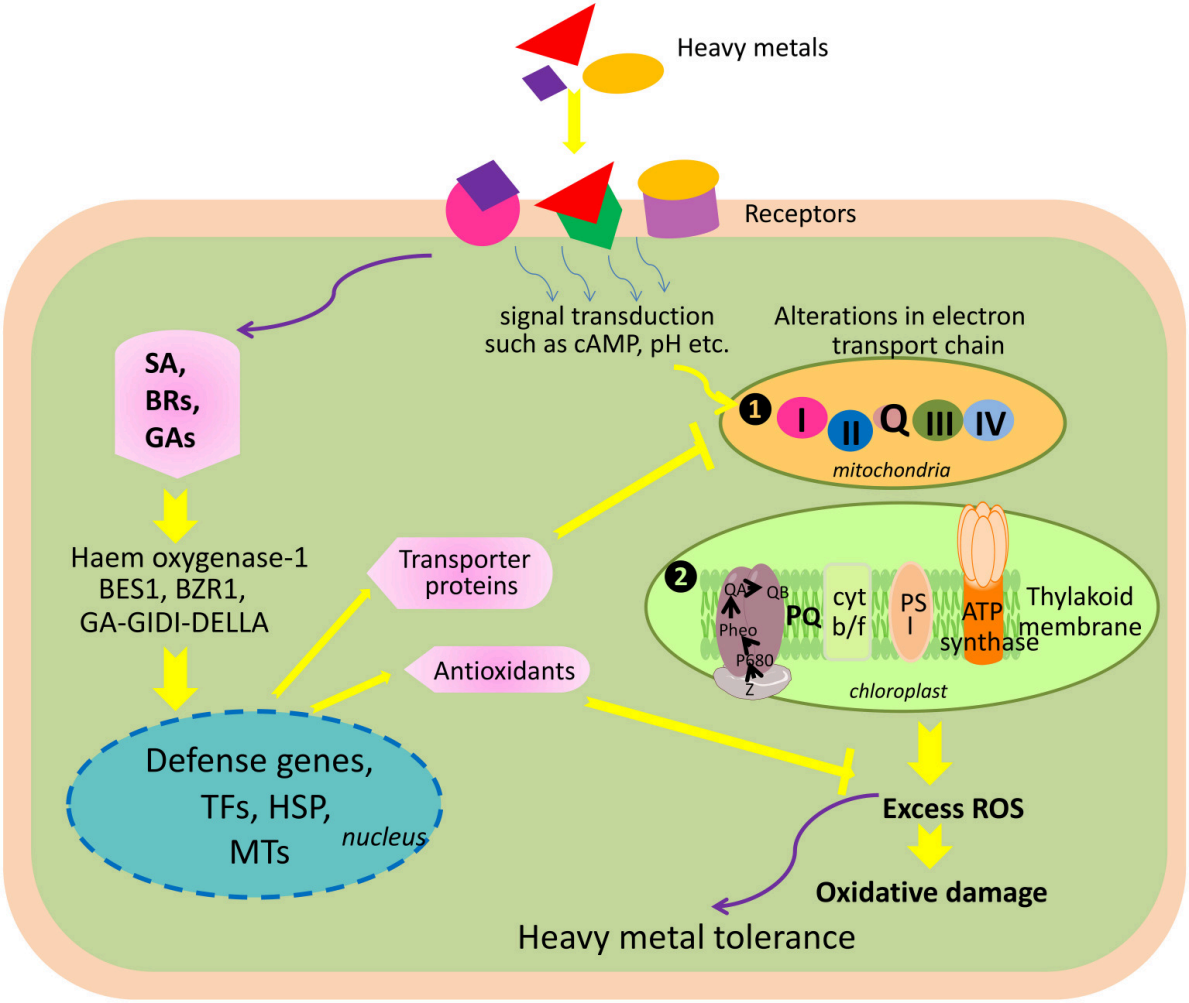
**Schematic representation of plant hormone-mediated alleviation of heavy metal toxicity in plants**. Heavy metals' signals are perceived by receptors, and receptors transduce signals *via* cAMP, pH, etc. causing alterations in electron transport systems of the cell, which results into an excess production of reactive oxygen species (ROS). ROS cause damage to macromolecules and thus create oxidative stress inside the cell. On the other hand, in the presence of plant hormones, signals received by them initiate a cascade of signal transduction involving haem oxygenase, two transcription factors induced by brassinosteroids (BES1 and BZR1) and a gibberellic acid-mediated GA-GID1-DELLA signaling pathway. These factors, in turn, initiate expression of the nuclear genes encoding defense proteins, transcription factors (TFs), heat shock proteins (HSP), and metal transporter proteins (MTs). MTs protect electron transport chains against heavy metals by regulating their uptake. Defense proteins protect plant against ROS under heavy metal stress (Numbers 1 and 2 designated to chloroplast and mitochondria show the sources of ROS in cell).

It is known that SA also involves in the regulation of oxidative stress caused by various stress factors (Yang et al., 2004). An enhanced level of SA under heavy metal stress suggests a connection between the extent of plant tolerance to heavy metal, which is mediated by the SA signal and the redox balance (Metwally et al., 2003; Sharma and Dietz, 2009). In the SA signaling under heavy metal stress, several signaling molecules such as nitric oxide (NO), H_2_O_2_, Ca^+2^, etc. and their interactions have been reported (Rodríguez-Serrano et al., 2009; Xu et al., 2014). Moreover, Cui et al. (2012b) have reported a cross-talk of haem oxygenase-1 and SA in alleviation of Cd stress in *M. sativa*. In spite of considerable progress in the understanding of SA signaling, molecular events, which are involved in the SA signaling in order to alleviate heavy metal stress, are still poorly known (Figure 4).

### Brassinosteroids (BRs)

Brassinosteroids are group of hormones having ability of regulating ion uptake in plant cells and very effectively reducing the heavy metal accumulation in plants. BRs can also impart plant stress tolerance against variety of biotic and abiotic stresses such as heavy metal, salinity, drought, low and high temperatures, and pathogen attack (Bajguz and Hayat, 2009; Hao et al., 2013). An increasing numbers of studies have shown that an exogenous application of BRs is widely used in order to improve crop yield as well as stress tolerance in various plant species (Divi and Krishna, 2009; Peleg and Blumwald, 2011; Li et al., 2013). Cadmium, a heavy metal, very toxic even when present in trace amount, have been found to retard chlorophyll biosynthesis, activity of several enzymes, and inhibit light and dark reactions of photosynthesis by limiting the energy/reducing sources (Vassilev and Yordanov, 1997). However, it has been reported that Cd-induced toxicity can be lowered with BR. For instance, Janeckzo et al. (2005) reported that Cd-induced inhibition in pigments content, cotyledon growth could be minimized with exogenous epibrassinolide (EPL: another BR). Hayat et al. (2007) have verified the role of HBL under Cd stress in *B. juncea*. In *Vigna radiata* L. Wilczek, Al stress caused a reduction in length, fresh and dry mass of root and shoot; activity of carbonic anhydrase; water use efficiency; relative water content; chlorophyll content; and the rate of photosynthesis, whereas addition of BR reversed these toxic effects and protected the plants *via* elevated level of proline in an association with an antioxidant defense system which at least in part was responsible for the amelioration of Al stress (Ali et al., 2008). In *B. juncea*, BR alleviates Cd toxicity through enhanced level of antioxidants (Hayat et al., 2007). The Cr, a known toxic metal, reduced the growth performance of *Raphanus sativus* L., whereas BR protects plants from adverse consequences of Cr toxicity by regulating antioxidant defense system (Sharma et al., 2011). Micronutrients such as Cu and Ni are essential for growth and development, but in excess, they cause severe toxic effects. Cu, which has increasingly attained interest due to its use in fungicides, fertilizers, and pesticides, is also highly toxic to plants, but when seeds of *B. juncea* primed with epibrassinolide (a form of BR) were exposed to Cu stress, improvement in shoot emergence and biomass accumulation, along with reduced Cu uptake and accumulation, was noticed (Sharma and Bhardwaj, 2007). Similar protective responses of exogenous BR on *B. juncea* and *V. radiata* under Cu and Ni toxicities have been reported (Alam et al., 2007; Sharma et al., 2008; Fariduddin et al., 2009; Yusuf et al., 2012b). Besides higher plants, BR has also been found to be effective in alleviating heavy metals such as Cu, Pb, and Cd toxicities in algae, *Chlorella vulgaris*, through the regulation of antioxidant defense system (Bajguz, 2010). The BR-mediated alterations in the gene expressions and their roles in stress tolerance are listed in Table 2.

### Gibberellic acid (GA)

The gibberellins (GAs) are a large family of tetracyclic diterpenoid plant growth hormone associated with the plant growth and developmental processes (Matsuoka, 2003). To alleviate deleterious effects of stress, different types of plant hormones have been used that might complement decreased and/or imbalanced hormone level during exposure of stress. Of these, GA has been a focus of plant scientists (Hisamatsu et al., 2000; Iqbal et al., 2011; Zhu et al., 2012).

Several studies revealed that GA alleviates various abiotic stresses including heavy metal toxicity. In *A. thaliana*, GA (5 μM) is reported to ameliorate Cd toxicity by reducing Cd uptake and lipid peroxidation (Zhu et al., 2012). Furthermore, authors demonstrated that GA reduces NO level which in turn down-regulates expression of *IRT1* gene, a Fe transporter (might be involved in Cd absorption) as indicated by no effect of GA in reduction of Cd uptake in an IRT1 knockout mutant *irt1*. It is reported that an exogenous addition of GA reprograms the growth of soybean under stress conditions by enhancing the levels of daidzein and genistein contents, suggesting protective role of GA in mitigating adverse consequences of stressors (Hamayun et al., 2010). In wheat seedlings, Ni (50 mM) has been shown to decline growth, chlorophyll content, and carbonic anhydrase activity by enhancing oxidative stress, whereas an addition of GA ameliorates Ni-induced toxic effects (Siddiqui et al., 2011). Gangwar et al. (2011) have also reported that an exogenous addition of GA ameliorates toxic effects of Cr (50–250 μM) on growth and ammonium assimilation of pea seedlings by regulating oxidative stress and an antioxidant system. In *B. napus* L., GA (50 μM) has been shown to alleviate Cd (10–400 μM)-induced negative impact on seed germination and growth by regulating oxidative stress and damage (Meng et al., 2009). It has been observed that Pb and Zn affect seed germination in *Cicer arietinum* cv. Aziziye-94 by altering hormonal balance, and an exogenous application of GA reverses the toxic effect of heavy metals (Atici et al., 2005). Furthermore, Sharaf et al. (2009) have also reported that GA mitigates detrimental effects of Cd and Pb on broad bean and lupin plants by regulating activities of proteases, CAT, and POD. These studies clearly indicate that GA plays an important role in protecting plant metabolism against various stresses; however, this may occur *via* various routes suggesting complex GA signaling during plant acclimation against stresses. The GA-mediated alterations in genes and their relation with stress tolerance are summarized in Table 4.

## Conclusion and future prospects

Around 3.1 billion people from developing countries live in rural areas, and out of this population, ~2.5 billion people depend on agricultural practices for their livelihood, which contributes 30% to economic growth because of the gross domestic products obtained from agriculture (FAO, 2012). It is expected that world population will be about 10 billion by the middle of the twenty-first century, and we will witness serious food shortages (Smith et al., 2010; Naika et al., 2013). Furthermore, the situation will likely to be severe due to increased anthropogenic activities that have resulted into unwanted changes in the environment such as soil, air, and water pollution with various factors including heavy metal. These situations (pollution and population) are posing a continuously increasing burden on global crop productivity, and hence, there are demands for crop varieties that should be adaptive and resistant to various stresses.

In contrast to biotic stress, which is under the control of monogenic trait, abiotic stress tolerance is a genetically complex process that involves many components of signaling pathways, multigenic in nature, and thus, comparatively more difficult to control and engineer (Vinocur and Altman, 2005). Therefore, plant-engineering strategies for heavy metal tolerance depend on the expression of gene(s) whose product(s) are involved either in signaling and regulatory pathways or in the synthesis of functional and structural proteins and metabolites that confer heavy metal stress tolerance. Recently, several efforts are being made to improve heavy metal stress tolerance capacity through genetic engineering with several achievements; however, the genetically complex mechanisms of heavy metal stress tolerance and transfer of technology to field conditions make it difficult. Advances in various functional tools, resources, and “omics” have helped in the molecular characterization of the genes, metabolites, and proteins involved in heavy metal stress tolerance. Furthermore, genetic engineering of heavy metal-responsive genes (particularly TFs), metabolites, and proteins has shown surprising results but its full potential remains to be exploited. The design of future experiments that use a multidisciplinary approach with well-integrated “omics,” i.e., transcritomics, metabolomics, proteomics, etc. ultimately required to significantly improve heavy metal tolerance as well as tolerance to other abiotic stresses in economically important crop plants.

### Conflict of interest statement

The authors declare that the research was conducted in the absence of any commercial or financial relationships that could be construed as a potential conflict of interest.

## References

[B1] Abul Kashem M. D. A. Kawai S. (2007). Alleviation of cadmium phytotoxicity by magnesium in Japanese mustard spinach. Soil Sci. Plant Nutr. 53, 246–251. 10.1111/j.1747-0765.2007.00129.x

[B2] Acquaah G. (2007). Principles of Plant Genetics and Breeding. Oxford, UK: Blackwell.

[B3] Ahammed G. J. Choudhary S. P. Chen S. Xia X. Shi K. Zhou Y.. (2013). Role of brassinosteroids in alleviation of phenanthrene-cadmium co-contamination-induced photosynthetic inhibition and oxidative stress in tomato. J. Exp. Bot. 64, 199–213. 10.1093/jxb/ers323 23201830 PMC3528031

[B4] Ahsan N. Lee D. G. Alam I. Kim P. J. Lee J. J. Ahn Y. O.. (2008). Comparative proteomic study of arsenic-induced differentially expressed proteins in rice roots reveals glutathione plays a central role during As stress. Proteomics 8, 3561–3576. 10.1002/pmic.200701189 18752204

[B5] Ahsan N. Lee D. G. Kim K. H. Alam I. Lee S. H. Lee K. W.. (2010). Analysis of arsenic stress-induced differentially expressed proteins in rice leaves by two-dimensional gel electrophoresis coupled with mass spectrometry. Chemosphere 78, 224–231. 10.1016/j.chemosphere.2009.11.004 19948354

[B6] Ahsan N. Lee D. G. Lee S. H. Kang K. Y. Lee J. J. Kim P. J.. (2007). Excess copper induced physiological and proteomic changes in germinating rice seeds. Chemosphere 67, 1182–1193. 10.1016/j.chemosphere.2006.10.075 17182080

[B7] Ahsan N. Renaut J. Komatsu S. (2009). Recent developments in the application of proteomics to the analysis of plant responses to heavy metal. Proteomics 9, 2602–2621. 10.1002/pmic.200800935 19405030

[B8] Alam M. M. Hayat S. Ali B. Ahmad A. (2007). Effect of 28-homobrassinolide treatment on nickel toxicity in *Brassica juncea*. Photosynthetica 45, 139–142. 10.1007/s11099-007-0022-4

[B9] Ali B. Hasan S. A. Hayat S. Hayat Q. Yadav S. Fariduddin Q. (2008). A role for brassinosteroids in the amelioration of aluminium stress through antioxidant system in mung bean (*Vigna radiata* L. Wilczek). Environ. Exp. Bot. 62, 153–159. 10.1016/j.envexpbot.2007.07.014

[B10] Ali B. Qian P. Jin R. Ali S. Khan M. Aziz R. (2013a). Physiological and ultra-structural changes in *Brassica napus* seedlings induced by cadmium stress. Biol. Plant. 58, 131–138. 10.1007/s10535-013-0358-5

[B11] Ali B. Wang B. Ali S. Ghani M. A. Hayat M. T. Yang C. (2013b). 5-Aminolevulinic acid ameliorates the growth, photosynthetic gas exchange capacity, and ultrastructural changes under cadmium stress in *Brassica napus* L. J. Plant Growth Regul. 32, 604–614. 10.1007/s00344-013-9328-6

[B12] Anderson N. L. Anderson N. G. (1998). Proteome and proteomics: new technologies, new concepts, and new words. Electrophoresis 19, 1853–1861. 10.1002/elps.1150191103 9740045

[B13] Anjum N. A. Gill S. S. Gill R. Hasanuzzaman M. Duarte A. C. Pereira E.. (2014). Metal/metalloid stress tolerance in plants: role of ascorbate, its redox couple, and associated enzymes. Protoplasma 251, 1265–1283. 10.1007/s00709-014-0636-x 24682425

[B14] Anjum N. A. Umar S. Ahmad A. Iqbal M. Khan N. A. (2008). Sulphur protects Mustard (*Brassica campestris* L.) from cadmium toxicity by improving leaf ascorbate and glutathione. Plant Growth Regul. 54, 271–279. 10.1007/s10725-007-9251-6

[B15] Apel K. Hirt H. (2004). Reactive oxygen species: metabolism, oxidative stress, and signal transduction. Annu. Rev. Plant Biol. 55, 373–399. 10.1146/annurev.arplant.55.031903.141701 15377225

[B16] Arbona V. Manzi M. Ollas C. D. Gonez-Cadenao A. (2013). Metabolites as a tool to investigate abiotic stress tolerance in plants. Int. J. Mol. Sci. 14, 4885–4911. 10.3390/ijms14034885 23455464 PMC3634444

[B17] Arenhart R. A. De Lima J. C. Pedron M. Carvalho F. E. L. Da Silveira J. A. G. Rosa S. B.. (2013). Involvement of ASR genes in aluminium tolerance mechanisms in rice. Plant Cell Environ. 36, 52–67. 10.1111/j.1365-3040.2012.02553.x 22676236

[B18] Arrivault S. Senger T. Krämer U. (2006). The Arabidopsis metal tolerance protein AtMTP3 maintains metal homeostasis by mediating Zn exclusion from the shoot under Fe deficiency and Zn oversupply. Plant J. 46, 861–879. 10.1111/j.1365-313X.2006.02746.x 16709200

[B19] Asada K. (1992). Ascorbate peroxidase: a hydrogen peroxide scavenging enzyme in plants. Plant Physiol. 85, 235–241. 10.1111/j.1399-3054.1992.tb04728.x

[B20] Asada K. (1996). Radical production and scavenging in chloroplasts, in Photosynthesis and the Environment, ed Baker N. (Dordrecht: Kluwer; Atlantic Canada Society for Microbial Ecology; Halifax), 123–150.

[B21] Assuncão A. G. L. Schat H. Aarts M. G. M. (2003). *Thlaspi caerulescens*, an attractive model species to study heavy metal hyperaccumulation in plants. New Phytol. 159, 351–360. 10.1046/j.1469-8137.2003.00820.x 33873356

[B22] Assunção A. G. Da Costa Martins P. De Folter S. Vooijs R. Schat H. Aarts M. G. M. (2001). Elevated expression of metal transporter genes in three accessions of the metal hyperaccumulator *Thlaspi caerulescens*. Plant Cell Environ. 24, 217–226. 10.1111/j.1365-3040.2001.00666.x

[B23] Assunção A. G. Herrero E. Lin Y. F. Huettel B. Talukdar S. Smaczniak C.. (2010). *Arabidopsis thaliana* transcription factors bZIP19 and bZIP23 regulate the adaptation to zinc deficiency. Proc. Natl. Acad. Sci. U.S.A. 107, 10296–10301. 10.1073/pnas.1004788107 20479230 PMC2890486

[B24] Astolfi S. Zuchi S. Passera C. (2004). Role of sulphur availability on cadmium-induced changes of nitrogen and sulphur metabolism in maize (*Zea mays* L.) Leaves. J. Plant Physiol. 161, 795–802. 10.1016/j.jplph.2003.11.005 15310068

[B25] Atici Ö. Ağar G. Battal P. (2005). Changes in phytohormone contents in chickpea seeds germinating under lead or zinc stress. Biol. Plant. 49, 215–222. 10.1007/s10535-005-5222-926817646

[B26] Atkinson N. J. Urwin P. E. (2012). The interaction of plant biotic and abiotic stresses: from genes to the field. J. Exp. Bot. 63, 3523–3544. 10.1093/jxb/ers100 22467407

[B27] Axelsen K. B. Palmgren M. G. (1998). Evolution of substrate specificities in the P-type ATPase superfamily. J. Mol. Evol. 46, 84–101. 10.1007/PL00006286 9419228

[B28] Azzarello E. Pandolfi C. Giordano C. Rossi M. Mugnai S. Mancuso S. (2012). Ultramorphological and physiological modifications induced by high zinc levels in *Paulownia tomentosa*. Environ. Exp. Bot. 81, 11–17. 10.1016/j.envexpbot.2012.02.008

[B29] Babu T. S. Akhtar T. A. Lampi M. A. Tripuranthakam S. Dixon D. G. Greenberg B. M. (2003). Similar stress responses are elicited by copper and ultraviolet radiation in the aquatic plant Lemnagibba: implication of reactive oxygen species as common signals. Plant Cell Phys. 44, 1320–1329. 10.1093/pcp/pcg160 14701927

[B30] Bajguz A. (2010). An enhancing effect of exogenous brassinolide on the growth and antioxidant activity in *Chlorella vulgaris* cultures under heavy metal stress. Environ. Exp. Bot. 68, 175–179. 10.1016/j.envexpbot.2009.11.003

[B31] Bajguz A. Hayat S. (2009). Effects of brassinosteroids on the plant responses to environmental stresses. Plant Physiol. Biochem. 47, 1–8. 10.1016/j.plaphy.2008.10.002 19010688

[B32] Barker A. V. Pilbeam D. J. (2007). Hand Book of Plant Nutrition. Boca Raton, FL: Taylor and Francis.

[B33] Bashri G. Prasad S. M. (2015). Indole acetic acid modulates changes in growth, chlorophyll a fluorescence and antioxidant potential of *Trigonella foenum-graecum* L. grown under cadmium stress. Acta Physiol. Plant. 37:1745 10.1007/s11738-014-1745-z

[B34] Basile A. Sorbo S. Pisani T. Paoli L. Munzi S. Loppi S. (2012). Bioacumulation and ultrastructural effects of Cd, Cu, Pb and Zn in the moss *Scorpiurum circinatum* (Brid.) Fleisch & Loeske. Environ. Pollut. 166, 208–211. 10.1016/j.envpol.2012.03.018 22516710

[B35] Basu U. Good A. G. Taylor G. J. (2001). Transgenic *Brassica napus* plants overexpressing aluminium-induced mitochondrial manganese superoxide dismutase cDNA are resistant to aluminium. Plant Cell Environ. 24, 1278–1269. 10.1046/j.0016-8025.2001.00783.x

[B36] Baszynski T. Wajda L. Krol M. Wolinska D. Krupa Z. Tukendorf A. (1980). Photosynthetic activities of cadmium-treated tomato plants. Physiol. Plant. 48, 365–370. 10.1111/j.1399-3054.1980.tb03269.x

[B37] Becher M. Talke I. N. Krall L. Krämer U. (2004). Cross-species microarray transcript profiling reveals high constitutive expression of metal homeostasis genes in shoots of the zinc hyperaccumulator *Arabidopsis halleri*. Plant J. 37, 251–268. 10.1046/j.1365-313X.2003.01959.x 14690509

[B38] Bellion M. Courbot M. Jacob C. Guinet F. Blaudez D. Chalot M. (2007). Metal induction of a *Paxillus involutus* metallothionein and its heterologous expression in *Hebeloma cylindrosporum*. New Phytol. 174, 151–158. 10.1111/j.1469-8137.2007.01973.x 17335505

[B39] Belokobylsky A. I. Ginturi E. I. Kuchava N. E. Kirkesali E. I. Mosulishvili L. M. Frontasyeva M. V. (2004). Accumulation of selenium and chromium in the growth dynamics of *Spirulina platensis*. J. Radioanal. Nucl. Chem. 259, 65–68. 10.1023/B:JRNC.0000015807.53132.c0

[B40] Belouchi A. Kwan T. Gros P. (1997). Cloning and characterization of the OsNramp family from *Oryza sativa*, a new family of membrane proteins possibly implicated in the transport of metal ions. Plant Mol. Biol. 33, 1085–1092. 10.1023/A:1005723304911 9154989

[B41] Bernal M. P. McGrath S. P. Miller A. J. Baker A. J. M. (1994). Comparison of the chemical changes in the rhizosphere of the nickel hyperaccumulator *Alyssum murale* with the non-accumulator *Raphanus sativus*. Plant Soil 64, 251–259. 10.1007/BF00010077

[B42] Bharwana S. A. Ali S. Farooq M. A. Iqbal N. Abbas F. Ahmad M. S. A. (2013). Alleviation of lead toxicity by silicon is related to elevated photosynthesis, antioxidant enzymes suppressed lead uptake and oxidative stress in cotton. J. Bioremed. Biodeg. 4, 4 10.4172/2155-6199.1000187 23911213

[B43] Blanvillain R. Wei S. Wei P. Kim J. H. Ow D. W. (2011). Stress tolerance to stress escape in plants: role of the OXS2 zinc-finger transcription factor family. EMBO J. 30, 3812–3822. 10.1038/emboj.2011.270 21829164 PMC3173794

[B44] Blaudez D. Kohler A. Martin F. Sanders D. Chalot M. (2003). Poplar metal tolerance protein 1 (MTP1) confers zinc tolerance and is an oligomeric vacuolar zinc transporter with an essential leucine zipper motif. Plant Cell 15, 2911–2928. 10.1105/tpc.017541 14630973 PMC282827

[B45] Bobrowicz P. Wysocki R. Owsianik G. Goffeau A. Ulaszewski S. (1997). Isolation of three contiguous genes, ACR1, ACR2 and ACR3, involved in resistance to arsenic compounds in the yeast *Saccharomyces cerevisiae*. Yeast 13, 819–828. 9234670 10.1002/(SICI)1097-0061(199707)13:9<819::AID-YEA142>3.0.CO;2-Y

[B46] Bona E. Marsano F. Cavaletto M. Berta G. (2007). Proteomic characterization of copper stress response in *Cannabis sativa* roots. Proteomics 7, 1121–1130. 10.1002/pmic.200600712 17352425

[B47] Borsani O. Valpuesta V. Botella M. A. (2001). Evidence for a role of salicylic acid in the oxidativedamage generated by NaCl and osmotic stress in *Arabidopsis* seedlings. Plant Physiol. 126, 1024–1030. 10.1104/pp.126.3.1024 11457953 PMC116459

[B48] Briat J. F. Curie C. Gaymard F. (2007). Iron utilization and metabolism in plants. Curr. Opin. Plant Biol. 10, 276–282. 10.1016/j.pbi.2007.04.003 17434791

[B49] Buendía-González L. Orozco-Villafuerte J. Cruz-Sosa F. Barrera-Díaz C. E. Vernon-Carter E. J. (2010). *Prosopis laevigata* a potential chromium (VI) and cadmium (II) hyperaccumulator desert plant. Biores. Technol. 101, 5862–5867. 10.1016/j.biortech.2010.03.027 20347590

[B50] Burló F. Guijarro I. Carbonell-Barrachina A. A. Vlaero D. Martínez-Sánchez F. (1999). Arsenic species: effects on and accumulation by tomato plants. J. Agric. Food Chem. 47, 1247–1253. 10.1021/jf9806560 10552445

[B51] Caille N. Zhao F. J. McGrath S. P. (2005). Comparison of root absorption, translocation and tolerance of arsenic in the hyperaccumulator *Pteris vittata* and the nonhyperaccumulator *Pteris tremula*. New Phytol. 165, 755–761. 10.1111/j.1469-8137.2004.01239.x 15720686

[B52] Cailliatte R. Lapeyre B. Briat J. F. Mari S. Curie C. (2009). The NRAMP6 metal transporter contributes to cadmium toxicity. Biochem. J. 422, 217–228. 10.1042/BJ20090655 19545236

[B53] Cailliatte R. Schikora A. Briat J. F. Mari S. Curie C. (2010). High-affinity manganese uptake by the metal transporter NRAMP1 is essential for Arabidopsis growth in low manganese conditions. Plant Cell 22, 904–917. 10.1105/tpc.109.073023 20228245 PMC2861449

[B54] Callahan D. L. Baker A. J. M. Kolev S. D. Wedd A. G. (2006). Metal ion ligands in hyperaccumulating plants. J. Biol. Inorg. Chem. 11, 2–12. 10.1007/s00775-005-0056-7 16328457

[B55] Carbonell A. A. Aarabi M. A. DeLaune R. D. Gambrell R. P. Patrick W. H. Jr. (1998). Arsenic in wetland vegetation: availability, phytotoxicity, uptake and effects on plant growth and nutrition. Sci. Total Environ. 217, 189–199. 10.1016/S0048-9697(98)00195-8

[B56] Carbonell-Barrachina A. A. Burló -Carbonell F. Mataix-Beneyto J. (1997). Effect of sodium arsenite and sodium chloride on bean plant nutrition (macronutrients). J. Plant Nutr. 20, 1617–1633. 10.1080/01904169709365361

[B57] Carbonell-Barrachina A. Burló -Carbonell F. Mataix-Beneyto J. (1994). Effect of arsenite on the concentration of micronutrients in tomato plants grown in hydroponic culture. J. Plant Nutr. 17, 1887–1903. 10.1080/01904169409364853

[B58] Castrillo G. Sánchez-Bermejo E. de Lorenzo L. Crevillén P. Fraile-Escanciano A. Tc M.. (2013). WRKY6 Transcription factor restricts arsenate uptake and transposon activation in *Arabidopsis*. Plant Cell 25, 2944–2957. 10.1105/tpc.113.114009 23922208 PMC3784590

[B59] Cataldo D. A. Garland T. R. Wildung R. E. (1983). Cadmium uptake kinetics in intact soybean plants. Plant Physiol. 73, 844–848. 10.1104/pp.73.3.844 16663310 PMC1066558

[B60] Cataldo D. A. McFadden K. M. Garland T. R. Wildung R. E. (1988). Organic constituents and complexation of nickel(II), iron(III), cadmium(II), and plutonium(IV) in soybean xylem exudates. Plant Physiol. 86, 734–739. 10.1104/pp.86.3.734 16665978 PMC1054560

[B61] Chaffei C. Gouia H. Ghorbel M. H. (2003). Nitrogen metabolism in tomato plants under cadmium stress. J. Plant Nutr. 26, 1617–1634. 10.1081/PLN-120022372

[B62] Chao D. Y. Chen Y. Chen J. Shi S. Chen Z. Wang C.. (2014). Genome-wide association mapping identifies a new arsenate reductase enzyme critical for limiting arsenic accumulation in plants. PLoS Biol. 12:e1002009. 10.1371/journal.pbio.1002009 25464340 PMC4251824

[B63] Chao D.-Y. Silva A. Baxter I. Huang Y. S. Nordborg M. Danku J.. (2012). Genome-wide association studies identify heavy metal ATPase3 as the primary determinant of natural variation in leaf cadmium in *Arabidopsis thaliana*. PLoS Biol. 8:e1002923. 10.1371/journal.pgen.1002923 22969436 PMC3435251

[B64] Chaurasia N. Mishra Y. Rai L. C. (2008). Cloning expression and analysis of phytochelatin synthase (pcs) gene from *Anabaena* sp. PCC 7120 offering multiple stress tolerance in *Escherichia coli*. Biochem. Biophys. Res. Commun. 376, 225–230. 10.1016/j.bbrc.2008.08.129 18775414

[B65] Chen J. Zhou J. Goldsbrough P. B. (1997). Characterization of phytochelatin synthase from tomato. Physiol. Plant. 101, 165–172. 10.1111/j.1399-3054.1997.tb01833.x

[B66] Chen Y. A. Chi W. C. Huang T. L. Lin C. Y. Quynh Nguyeh T. T. Hsuing Y. C.. (2012a). Mercury-induced biochemical and proteomic changes in rice roots. Plant Physiol. Biochem. 55, 23–32. 10.1016/j.plaphy.2012.03.008 22522577

[B67] Chen Z. Pan Y. Wang S. Ding Y. Yang W. Zhu C. (2012b). Overexpression of a protein disulfide isomerase-like protein from *Methanothermobacter thermoautotrophicum* enhances mercury tolerance in transgenic rice. Plant Sci. 197, 10–20. 10.1016/j.plantsci.2012.08.005 23116667

[B68] Chiang C. M. Chen S. P. Chen L. F. O. Chiang M. C. Chien H. L. Lin K. H. (2013). Expression of the broccoli catalase gene (*BoCAT*) enhances heat tolerance in transgenic *Arabidopsis*. J. Plant Biochem. Biotechnol. 23, 266–277. 10.1007/s13562-013-0210-1

[B69] Cho S. H. Hoang Q. Phee J. W. Kim Y. Y. Shin H. Y. Shin J. S. (2007). Modified suppression subtractive hybridization identifies an AP2-containing protein involved in metal responses in *Physcomitrella patens*. Mol. Cells 23, 100–107. 17464218

[B70] Chong-qing W. Tao W. Ping M. Zi-chao L. Ling Y. (2013). Quantitative trait loci for mercury tolerance in rice seedlings. Rice Sci. 20, 238–242. 10.1016/S1672-6308(13)60124-9

[B71] Chou T. S. Chao Y. Y. Huang W. D. Hong C. Y. Kao C. H. (2011). Effect of magnesium deficiency on antioxidant status and cadmium toxicity in rice seedlings. J. Plant Physiol. 168, 1021–1030. 10.1016/j.jplph.2010.12.004 21216027

[B72] Choudhary S. P. Kanwar M. Bhardwaj R. Yu J. Q. Tran L. S. P. (2012b). Chromium stress mitigation by polyamine-brassinosteroid application involves phytohormonal and physiological strategies in *Raphanus sativus* L. PLoS ONE 7:e33210. 10.1371/journal.pone.0033210 22479371 PMC3315560

[B73] Choudhary S. P. Oral H. V. Bhardwaj R. Yu J. Q. Tran L. S. P. (2012a). Interaction of brassinosteroids and polyamines enhances copper stress tolerance in *Raphanus sativus*. J. Exp. Bot. 63, 5659–5675. 10.1093/jxb/ers219 22915739 PMC3444278

[B74] Cobbett C. S. (2000). Phytochelatin biosynthesis and function in heavy-metal detoxification. Curr. Opin. Plant Biol. 3, 211–216. 10.1016/S1369-5266(00)00066-2 10837262

[B75] Cobbett C. Goldsbrough P. (2002). Phytochelatins and metallothioneins: roles in heavy metal detoxification and homeostasis. Annu. Rev. Plant Biol. 53, 159–182. 10.1146/annurev.arplant.53.100301.135154 12221971

[B76] Collin V. C. Eymery F. Genty B. Rey P. Havaux M. (2008). Vitamin E is essential for the tolerance of *Arabidopsis thaliana* to metal induced oxidative stress. Plant Cell Environ. 31, 244–257. 10.1111/j.1365-3040.2007.01755.x 17996014

[B77] Creissen G. Edwards E. A. Enard C. Wellburn A. Mullineaux P. (1992). Molecular characterisation of glutathione reductase cDNAs from pea (*Pisum sativum* L.). Plant J. 2, 129–131. 1303792

[B78] Cui Y. Zhang X. Zhu Y. (2008). Does copper reduce cadmium uptake by different rice genotypes? J. Environ. Sci. 20, 332–338. 10.1016/S1001-0742(08)60052-2 18595401

[B79] Cui F. Liu L. Zhao Q. Zhang Z. Li Q. Lin B.. (2012a). *Arabidopsis* ubiquitin conjugase UBC32 is an ERAD component that functions in brassinosteroid-mediated salt stress tolerance. Plant Cell 24, 233–244. 10.1105/tpc.111.093062 22214659 PMC3289556

[B80] Cui W. Li L. Gao Z. Wu H. Xie Y. Shen W. (2012b). Haem oxygenase-1 is involved in salicylic acid-induced alleviation of oxidative stress due to cadmium stress in *Medicago sativa*. J. Exp. Bot. 63, 5521–5534. 10.1093/jxb/ers201 22915740 PMC3444266

[B81] Curie C. Alonso J. M. Le Jean M. Ecker J. R. Briat J. F. (2000). Involvement of NRAMP1 from *Arabidopsis thaliana* in iron transport. Biochem. J. 347, 749–755. 10.1042/bj3470749 10769179 PMC1221012

[B82] Dalcorso G. Fasani E. Furini A. (2013b). Recent advances in the analysis of metal hyperaccumulation and hypertolerance in plants using proteomics. Front. Plant Sci. 4:280. 10.3389/fpls.2013.00280 23898342 PMC3724048

[B83] DalCorso G. Manara A. Furini A. (2013a). An overview of heavy metal challenge in plants: from roots to shoots. Metallomics 5, 1117–1132. 10.1039/c3mt00038a 23739766

[B84] Davidson J. F. Schiestl R. H. (2001). Mitochondrial respiratory electron carriers are involved in oxidative stress during heat stress in *Saccharomyces cerevisiae*. Mol. Cell. Biol. 21, 8483–8489. 10.1128/MCB.21.24.8483-8489.2001 11713283 PMC100011

[B85] Davletova S. Rizhsky L. Liang H. Shengqiang Z. Oliver D. J. Coutu J.. (2005). Cytosolic ascorbate peroxidase 1 is a central component of the reactive oxygen gene network of *Arabidopsis*. Plant Cell 17, 268–281. 10.1105/tpc.104.026971 15608336 PMC544504

[B86] Delhaize E. Gruber B. D. Pittman J. K. White R. G. Leung H. Miao Y.. (2007). A role for the AtMTP11 gene of Arabidopsis in manganese transport and tolerance. Plant J. 51, 198–210. 10.1111/j.1365-313X.2007.03138.x 17559518

[B87] Delhaize E. Kataoka T. Hebb D. M. White R. G. Ryan P. R. (2003). Genes encoding proteins of the cation diffusion facilitator family that confer manganese tolerance. Plant Cell 15, 1131–1142. 10.1105/tpc.009134 12724539 PMC153721

[B88] Desbrosses-Fonrouge A. G. Voigt K. Schröder A. Arrivault S. Thomine S. Krämer U. (2005). *Arabidopsis thaliana* MTP1 is a Zn transporter in the vacuolar membrane which mediates Zn detoxification and drives leaf Zn accumulation. FEBS Lett. 579, 4165–4174. 10.1016/j.febslet.2005.06.046 16038907

[B89] Diáz J. Bernal A. Po Mar F. Merino F. (2001). Induction of shikimate dehydrogenase and peroxidase in pepper (*Capsicum annum* L.) seedlings in response to copper stress and its relation to lignification. Plant Sci. 161, 179 10.1016/S0168-9452(01)00410-1

[B90] Divi U. K. Krishna P. (2009). Brassinosteroid: a biotechnological target for enhancing crop yield and stress tolerance. New Biotechnol. 26, 131–136. 10.1016/j.nbt.2009.07.006 19631770

[B91] Dixit P. Mukherjee P. K. Ramachandran V. Eapen S. (2011). Glutathione transferase from *Trichoderma virens* enhances cadmium tolerance without enhancing its accumulation in transgenic *Nicotiana tabacum*. PLoS ONE 6:e16360. 10.1371/journal.pone.0016360 21283689 PMC3024989

[B92] Dixon W. J. Inouye C. Karin M. Tullius T. D. (1996). CUP2 binds in a bipartite manner to upstream activation sequence c in the promoter of the yeast copper metallothionein gene. J. Biol. Inorg. Chem. 1, 451–459. 10.1007/s007750050078

[B93] Doncheva S. Ameńos M. Poschenrieder C. Barceĺo J. (2005). Root cell patterning: a primary target for aluminium toxicity in maize. J. Exp. Bot. 56, 1213–1220. 10.1093/jxb/eri115 15737983

[B94] Dong J. Wu F. B. Zhang G. P. (2005). Effect of cadmium on growth and photosynthesis of tomato seedlings. J. Zhejiang Univ. Sci. B 6, 974–980. 10.1631/jzus.2005.B0974 16187410 PMC1390439

[B95] Dräger D. B. Desbrosses-Fonrouge A. G. Krach C. Chardonnens A. N. Meyer R. C. Saumitou-Laprade P.. (2004). Two genes encoding *Arabidopsis halleri* MTP1 metal transport proteins co-segregate with zinc tolerance and account for high MTP1 transcript levels. Plant J. 39, 425–439. 10.1111/j.1365-313X.2004.02143.x 15255871

[B96] Dragišić Maksimović J. Mojović M. Maksimović V. Römheld V. Nikolic M. (2012). Silicon ameliorates manganese toxicity in cucumber by decreasing hydroxyl radical accumulation in the leaf apoplast. J. Exp. Bot. 63, 2411–2420. 10.1093/jxb/err359 22249995

[B97] Duan G.-L. Zhou Y. Tong Y.-P. Mukhopadhyay R. Rosen B. P. Zhu Y.-G. (2007). A CDC25 homologue from rice functions as an arsenate reductase. New Phytol. 174, 311–321. 10.1111/j.1469-8137.2007.02009.x 17388894

[B98] Eapen S. D'souza S. F. (2005). Prospects of genetic engineering of plants for phytoremediation of toxic metal. Biotechnol. Adv. 23, 97–114. 10.1016/j.biotechadv.2004.10.001 15694122

[B99] Eide D. Broderius M. Fett J. Guerinot M. L. (1996). A novel iron-regulated metal transporter from plants identified by functional expression in yeast. Proc. Natl. Acad. Sci. U.S.A. 93, 5624–5628. 10.1073/pnas.93.11.5624 8643627 PMC39298

[B100] Elbaz B. Shoshani-Knaani N. David-Assael O. Mizrachy-Dagri T. Mizrahi K. Saul H.. (2006). High expression in leaves of the zinc hyperaccumulator *Arabidopsis halleri* of AhMHX, a homolog of an *Arabidopsis thaliana* vacuolar metal/protonexchanger. Plant Cell Environ. 29, 1179–1190. 10.1111/j.1365-3040.2006.01500.x 17080942

[B101] Eleftheriou E. P. Adamakis I. D. Melissa P. (2012). Effects of hexavalent chromium on microtubule organization, ER distribution and callose deposition in root tip cells of *Allium cepa* L. Protoplasma 249, 401–416. 10.1007/s00709-011-0292-3 21633932

[B102] Ellis D. R. Gumaelius L. Indriolo E. Pickering I. J. Banks J. A. Salt D. E. (2006). A novel arsenate reductase from the arsenic hyperaccumulating fern *Pteris vittata*. Plant Physiol. 141, 1544–1554. 10.1104/pp.106.084079 16766666 PMC1533930

[B103] Epstein E. (1999). Silicon. Annu. Rev. Plant Physiol. Plant Mol. Biol. 50, 641–664. 10.1146/annurev.arplant.50.1.641 15012222

[B104] Ernst W. H. O. (2006). Evolution of metal tolerance in higher plants. For. Snow Landsc. Res. 80, 251–274. Available online at: http://www.wsl.ch/dienstleistungen/publikationen/pdf/7764.pdf

[B105] Ernst W. H. O. Krauss G. J. Verkleij J. A. C. Wesenberg D. (2008). Interaction of heavy metal with the sulphur metabolism in angiosperms from an ecological point of view. Plant Cell Environ. 31, 123–143. 10.1111/j.1365-3040.2007.01746.x 17999660

[B106] Esposito S. Sorbo S. Conte B. Basile A. (2012). Effects of heavy metal on ultrastructure and HSP70s induction in the aquatic moss *Leptodictyum riparium* Hedw. Int. J. Phytoremediation 14, 443–455. 10.1080/15226514.2011.620904 22567723

[B107] Fahr M. Laplaze L. Bendaou N. Hocher V. Mzibri M. E. Bogusz D.. (2013). Effect of lead on root growth. Front. Plant Sci. 4:175. 10.3389/fpls.2013.00175 23750165 PMC3674728

[B108] FAO (2012). Statistical Yearbook Viale delle Terme di Caracalla. Rome.

[B109] Farias J. G. Antes F. L. G. Nunes P. A. A. Nunes S. T. Schaich G. Rossato L. V. (2013). Effects of excess copper in vineyard soils on the mineral nutrition of potato genotypes. Food Energy Security 2, 49–69. 10.1002/fes3.16

[B110] Fariduddin Q. Yusuf M. Hayat S. Ahmad A. (2009). Effect of 28-homobrassinolide on antioxidant capacity and photosynthesis in *Brassica juncea* plants exposed to different levels of copper. Environ. Exp. Bot. 66, 418–424. 10.1016/j.envexpbot.2009.05.001

[B111] Farzadfar S. Zarinkamar F. Modarres-Sanavy S. A. M. Hojati M. (2013). Exogenously applied calcium alleviates cadmium toxicity in *Matricaria chamomilla* L. Plants Environ. Sci. Pollut. Res. 20, 1413–1422. 10.1007/s11356-012-1181-9 22968674

[B112] Feng R. W. Wei C. Y. (2012). Antioxidative mechanisms on selenium accumulation in *Pteris vittata* L., a potential selenium phytoremediation plant. Plant Soil Environ. 58, 105–110. Available online at: http://www.agriculturejournals.cz/publicFiles/61470.pdf

[B113] Feng R. W. Wei C. Y. Tu S. X. Tang S. R. Wu F. C. (2011). Detoxification of antimony by selenium and their interaction in paddy rice under hydroponic conditions. Microchem. J. 97, 57–61. 10.1016/j.microc.2010.06.003

[B114] Ferretti M. Ghisi R. Merlo L. Dalla Vecchia F. Passera C. (1993). Effect of cadmium on photosynthesis and enzymes of photosynthetic sulfate and nitrate assimilation pathways in maize (*Zea mays* L.). Photosynthetica 29, 49–54.

[B115] Fidalgo F. Azenha M. Silva A. F. de Sousa A. Santiago A. Ferraz P. (2013). Copper-induced stress in *Solanum nigrum* L. and antioxidant defense system responses. Food Energy Security 2, 70–80. 10.1002/fes3.20

[B116] Filek M. Keskinen R. Hartikainen H. Szarejko I. Janiak A. Miszalski Z.. (2008). The protective role of selenium in rape seedlings subjected to cadmium stress. J. Plant Physiol. 165, 833–844. 10.1016/j.jplph.2007.06.006 17913288

[B117] Fontes R. L. S. Cox F. R. (1998). Zinc toxicity in soybean grown at high iron concentration in nutrient solution. J. Plant Nutr. 21, 1723–1730. 10.1080/01904169809365517

[B118] Foreman J. Demidchik V. Bothwell J. H. Mylona P. Miedema H. Torres M. A.. (2003). Reactive oxygen species produced by NADPH oxidase regulate plant cell growth. Nature 422, 442–446. 10.1038/nature01485 12660786

[B119] Foyer C. H. Halliwell B. (1976). Presence of glutathione and glutathione reductase in chloroplasts: a proposed role in ascorbic acid metabolism. Planta 133, 21–25. 10.1007/BF00386001 24425174

[B120] Foyer C. H. Lopez-Delgado H. Dat J. F. Scott I. M. (1997). Hydrogen peroxide and glutathione-associated mechanisms of acclamatory stress tolerance and signalling. Physiol. Plant. 100, 241–254. 10.1111/j.1399-3054.1997.tb04780.x

[B121] Freeman J. L. Salt D. E. (2007). The metal tolerance profile of *Thlaspi goesingense* is mimicked in *Arabidopsis thaliana* heterologously expressing serine acetyl-transferase. BMC Plant Biol. 7:63. 10.1186/1471-2229-7-63 18045473 PMC2233625

[B122] Freeman J. L. Garcia D. Kim D. Hopf A. Salt D. E. (2005a). Constitutively elevated salicylic acid signals glutathione-mediated nickel tolerance in *Thlaspi* nickel hyperaccumulators. Plant Physiol. 137, 1082–1091. 10.1104/pp.104.055293 15734913 PMC1065408

[B123] Freeman J. L. Persans M. W. Nieman K. Albrecht C. Peer W. Pickering I. J.. (2004). Increased glutathione biosynthesis plays a role in nickel tolerance in *Thlaspi* nickel hyperaccumulators. Plant Cell 16, 2176–2191. 10.1105/tpc.104.023036 15269333 PMC519206

[B124] Freeman J. L. Persans M. W. Nieman K. Salt D. E. (2005b). Nickel and cobalt resistance engineered in *Escherichia coli* by overexpression of serine acetyltransferase from the nickel hyperaccumulator plant *Thlaspi goesingense*. Appl. Environ. Microbiol. 71, 8627–8633. 10.1128/AEM.71.12.8627-8633.2005 16332856 PMC1317400

[B125] Frérot H. Faucon M. P. Willems G. Godé C. Courseaux A. Darracq A.. (2010). Genetic architecture of zinc hyperaccumulation in *Arabidopsis halleri*: the essential role of QTLx environment interactions. New Phytol. 187, 355–367. 10.1111/j.1469-8137.2010.03295.x 20487314

[B126] Fridovich I. (1989). Superoxide dismutases: an adaptation to paramagnetic gas. J. Biol. Chem. 264, 7761–7764. 2542241

[B127] Führs H. Behrens C. Gallien S. Heintz D. Van Dorsselaer A. Braun H. P.. (2010). Physiological and proteomic characterization of manganese sensitivity and tolerance in rice (*Oryza sativa*) in comparison with barley (*Hordeum vulgare*). Ann. Bot. 105, 1129–1140. 10.1093/aob/mcq046 20237113 PMC2887067

[B128] Führs H. Hartwig M. Molina L. E. Heintz D. Van Dorsselaer A. Braun H. P.. (2008). Early manganese-toxicity response in *Vigna unguiculata* L. a proteomic and transcriptomic study. Proteomics 8, 149–159. 10.1002/pmic.200700478 18095375

[B129] Fukao Y. Ferjani A. Tomioka R. Nagasaki N. Kurata R. Nishimori Y.. (2011). iTRAQ analysis reveals mechanisms of growth defects due to excess zinc in *Arabidopsis*. Plant Physiol. 155, 1893–1907. 10.1104/pp.110.169730 21325567 PMC3091079

[B130] Fürst P. Hu S. Hackett R. Hamer D. (1988). Copper activates metallothionein gene transcription by altering the conformation of a specific DNA binding protein. Cell 55, 705–717. 10.1016/0092-8674(88)90229-2 3052856

[B131] Galeas M. L. Zhang L. H. Freeman J. L. Wegner M. Pilon-Smits E. A. H. (2007). Seasonal fluctuations of selenium and sulphur accumulation in selenium hyperaccumulators and related nonaccumulators. New Phytol. 173, 517–525. 10.1111/j.1469-8137.2006.01943.x 17244046

[B132] Gangwar S. Singh V. P. (2011). Indole acetic acid differently changes growth and nitrogen metabolism in *Pisum sativum* L. seedlings under chromium (VI) phytotoxicity: implication of oxidative stress. Sci. Hortic. 129, 321–328. 10.1016/j.scienta.2011.03.026

[B133] Gangwar S. Singh V. P. Prasad S. M. Maurya J. N. (2010). Modulation of manganese toxicity in *Pisum sativum* L. seedlings by kinetin. Sci. Hortic. 126, 467–474. 10.1016/j.scienta.2010.08.013

[B134] Gangwar S. Singh V. P. Srivastava P. K. Maurya J. N. (2011). Modification of chromium (VI) phytotoxicity by exogenous gibberellic acid application in *Pisum sativum* (L.) seedlings. Acta Physiol. Plant. 33, 1385–1397. 10.1007/s11738-010-0672-x

[B135] Gao J. Sun L. Yang X. Liu J. X. (2013). Transcriptomic analysis of cadmium stress response in the heavy metal hyperaccumulator *Sedum alfredii* Hance. PLoS ONE 8:e64643. 10.1371/journal.pone.0064643 23755133 PMC3670878

[B136] Garzón T. Gunsé B. Moreno A. R. Tomos A. D. Barceló J. Poschenrieder C. (2011). Aluminium-induced alteration of ion homeostasis in root tip vacuoles of two maize varieties differing in Al tolerance. Plant Sci. 180, 709–715. 10.1016/j.plantsci.2011.01.022 21421422

[B137] Ghosh M. Shen J. Rosen B. P. (1999). Pathways of As(III) detoxification in *Saccharomyces cerevisiae*. Proc. Natl. Acad. Sci. U.S.A. 96, 5001–5006. 10.1073/pnas.96.9.5001 10220408 PMC21806

[B138] Gichner T. Patkova Z. Szakova J. Demnerova K. (2004). Cadmium induces DNA damages in tobacco roots, but no DNA damage, somatic mutations orhomologous recombinations in tobacco leaves. Mutat. Res. Genet. Toxicol. Environ. Mut. 559, 49–57. 10.1016/j.mrgentox.2003.12.008 15066573

[B139] Gill S. S. Hasanuzzaman M. Nahar K. Macovei A. Tuteja N. (2013). Importance of nitric oxide in cadmium stress tolerance in crop plants. Plant Physiol. Biochem. 63, 254–261. 10.1016/j.plaphy.2012.12.001 23313792

[B140] Gomes M. M. A. (2011). Physiological effects related to brassinosteroid application in plants, in Brassinosteroids: A Class of Plant Hormone, eds Hayat S. Ahmad A. (Dordrecht: Springer), 193–242. 10.1007/978-94-007-0189-2_7

[B141] Grennan A. K. (2009). Identification of genes involved in metal transport in plants. Plant Physiol. 149, 1623–1624. 10.1104/pp.109.900287 19339509 PMC2663745

[B142] Guan Z. Chai T. Zhang Y. Xu J. Wei W. (2009). Enhancement of Cd tolerance in transgenic tobacco plants overexpressing a Cd-induced catalase cDNA. Chemosphere 76, 623–630. 10.1016/j.chemosphere.2009.04.047 19473687

[B143] Guo B. Liang Y. Zhu Y. (2009). Does salicylic acid regulate antioxidant defense system, cell death, cadmium uptake and partitioning to acquire cadmium tolerance in rice? J. Plant Physiol. 166, 20–31. 10.1016/j.jplph.2008.01.002 18313167

[B144] Guo J. Dai X. Xu W. Ma M. (2008a). Overexpressing GSHI and AsPCSI simultaneously increases the tolerance and accumulation of cadmium and arsenic in *Arabidopsis thaliana*. Chemosphere 72, 1020–1026. 10.1016/j.chemosphere.2008.04.018 18504054

[B145] Guo W. J. Meetam M. Goldsbrough P. (2008b). Examining the specific contributions of individual *Arabidopsis* metallothioneins to copper distribution and metal tolerance. Plant Physiol. 164, 1697–1706. 10.1104/pp.108.115782 18287486 PMC2287344

[B146] Gustin J. L. Zanis M. J. Salt D. E. (2011). Structure and evolution of the plant cation diffusion facilitator family of ion transporters. BMC Evol. Biol. 11:76. 10.1186/1471-2148-11-76 21435223 PMC3073911

[B147] Gygi S. P. Rochon Y. Franza B. R. Aebersold R. (1999). Correlation between protein and mRNA abundance in yeast. Mol. Cell. Biol. 19, 1720–1730. 10.1128/MCB.19.3.1720 10022859 PMC83965

[B148] Haag-Kerwer A. Schäfer H. J. Heiss S. Walter C. Rausch T. (1999). Cadmium exposure in *Brassica juncea* causes a decline in transpiration rate and leaf expansion without effect on photosynthesis. J. Exp. Bot. 50, 1827–1835. 10.1093/jxb/50.341.1827

[B149] Hajduch M. Rakwal R. Agrawal G. K. Yonekura M. Pretova A. (2001). High-resolution two-dimensional electrophoresis separation of proteins from metal-stressed rice (*Oryza sativa* L.) leaves: drastic reductions/fragmentation of ribulose-1,5-bisphosphate carboxylase/oxygenase and induction of stress-related proteins. Electrophoresis 22, 2824–2831. 10.1002/1522-2683(200108)22:13<2824::AID-ELPS2824>3.0.CO;2-C 11545414

[B150] Hall J. L. Williams L. E. (2003). Transition metal transporters in plants. J. Exp. Bot. 54, 2601–2613. 10.1093/jxb/erg303 14585824

[B151] Halliwell B. (2006). Reactive species and antioxidants. Redox biology is a fundamental theme of aerobic life. Plant Physiol. 141, 312–322. 10.1104/pp.106.077073 16760481 PMC1475431

[B152] Hamayun M. Khan S. A. Khan A. L. Shin J. H. Ahmad B. Shin D. H. (2010). Exogenous gibberellic acid reprograms soybean to higher growth and salt stress tolerance. J. Agric. Food Chem. 58, 7226–7232. 10.1021/jf101221t 20509656

[B153] Hamer D. H. (1986). Metallothionein. Annu. Rev. Biochem. 55, 913–951. 10.1146/annurev.bi.55.070186.004405 3527054

[B154] Haney C. J. Grass G. Franke S. Rensing C. (2005). New developments in the understanding of the cation diffusion facilitator family. J. Ind. Microbiol. Biotechnol. 32, 215–226. 10.1007/s10295-005-0224-3 15889311

[B155] Hao J. Yin Y. Fei S. Z. (2013). Brassinosteroid signaling network: implications on yield and stress tolerance. Plant Cell Rep. 32, 1017–1030. 10.1007/s00299-013-1438-x 23568410

[B156] Harrison P. M. Arosio P. (1996). The ferritins: molecular properties, iron storage function and cellular regulation. Biochim. Biophys. Acta 275, 161–203. 10.1016/0005-2728(96)00022-9 8695634

[B157] Hart J. J. Welch R. M. Norvell W. A. Clarke J. M. Kochian L. V. (2005). Zinc effects on cadmium accumulation and partitioning in near isogenic lines of durum wheat that differ in grain cadmium concentration. New Phystol. 167, 391–401. 10.1111/j.1469-8137.2005.01416.x 15998393

[B158] Hassan M. J. Zhang G. Wu F. Wei K. Chen Z. (2005). Zinc alleviates growth inhibition and oxidative stress caused by cadmium in rice. J. Plant Nutr. Soil Sci. 168, 255–261. 10.1002/jpln.200420403

[B159] Hayat Q. Hayat S. Irfan M. Ahmad A. (2010). Effect of exogenous salicylic acid under changing environment: a review. Environ. Exp. Bot. 68, 14–25. 10.1016/j.envexpbot.2009.08.005

[B160] Hayat S. Ali B. Aiman Hasan S. Ahmad A. (2007). Brassinosteroid enhanced the level of antioxidants under cadmium stress in *Brassica juncea*. Environ. Exp. Bot. 60, 33–41. 10.1016/j.envexpbot.2006.06.002

[B161] Hayat S. Khalique G. Irfan M. Wani A. S. Tripathi B. N. Ahmad A. (2012). Physiological changes induced by chromium stress in plants: an overview. Protoplasma 249, 599–611. 10.1007/s00709-011-0331-0 22002742

[B162] He H. Zhan J. He L. Gu M. (2012). Nitric oxide signaling in aluminum stress in plants. Protoplasma 249, 483–492. 10.1007/s00709-011-0310-5 21850424

[B163] He P. P. Lv X. Z. Wang G. Y. (2004). Effects of Se and Zn supplementation on the antagonism against Pb and Cd in vegetables. Environ. Int. 30, 167–172. 10.1016/S0160-4120(03)00167-3 14749105

[B164] Herbette S. Taconnat L. Hugouvieux V. Piette L. Magniette M. L. Cuine S.. (2006). Genome-wide transcriptome profiling of the early cadmium response of *Arabidopsis* roots and shoots. Biochimie 88, 1751–1765. 10.1016/j.biochi.2006.04.018 16797112

[B165] Herbik A. Giritch A. Horstmann C. Becker R. Balzer H. J. Bäumlein H.. (1996). Iron and copper nutrition-dependent changes in protein expression in a tomato wild type and the nicotianamine-free mutant chloronerva. Plant Physiol. 111, 533–540. 10.1104/pp.111.2.533 8787027 PMC157864

[B166] Hernández L. E. Garate A. Carpena-Ruiz R. (1997). Effects of cadmium on the uptake, distribution and assimilation of nitrate in *Pisum sativum*. Plant Soil. 189, 97–106.

[B167] Hernandez L. E. Villasante C. O. Montero-Palmero M. B. Escobar C. Carpena R. O. (2012). Heavy metal perception in a microscale environment: a model system using high doses of pollutants, in Metal Toxicity in Plants: Perception, Signaling and Remediation, eds Gupta D. K. Sandalio L. M. (Berlin; Heidelberg: Springer-Verlag), 23–37. 10.1007/978-3-642-22081-4_2

[B168] Hirata K. Tsuji N. Miyamoto K. (2005). Biosynthetic regulation of phytochelatins, heavy metal-binding peptides. J. Biosci. Bioeng. 100, 593–599. 10.1263/jbb.100.593 16473766

[B169] Hirschi K. D. Korenkov V. D. Wilganowski N. L. Wagner G. J. (2000). Expression of *Arabidopsis* CAX2 in tobacco. Altered metal accumulation and increased manganese tolerance. Plant Physiol. 124, 125–133. 10.1104/pp.124.1.125 10982428 PMC59128

[B170] Hisamatsu T. Koshioka M. Kubota S. Fujime Y. King R. W. Mander L. N. (2000). The role of gibberellin in the control of growth and flowering in *Matthiola incana*. Physiol. Plant. 109, 97–105. 10.1034/j.1399-3054.2000.100114.x

[B171] Horváth E. Szalai G. Janda T. (2007). Induction of abiotic stress tolerance by salicylic acid signaling. J. Plant Growth Regul. 26, 290–300. 10.1007/s00344-007-9017-4

[B172] Hossain M. A. Hasanuzzaman M. Fujita M. (2011). Coordinate induction of antioxidant defense and glyoxalase system by exogenous proline and glycinebetaineis correlated with salt tolerance in mung bean. Front. Agric. China 5, 1–14. 10.1007/s11703-010-1070-2

[B173] Hossain M. A. Hossain M. D. Rohman M. M. da Silva J. A. T. Fujita M. (2012b). Onion major compounds (flavonoids, organosulfurs) and highly expressed glutathione-related enzymes: possible physiological interaction, gene cloning and abiotic stress response, in Onion Consumption and Health, eds Aguirre C. B. Jaramillo L. M. (New York, NY: Nova Science Publishers), 49–90.

[B174] Hossain M. A. Piyatida P. da Silva J. A. T. Fujita M. (2012a). Molecular mechanism of heavy metal toxicity and tolerance in plants: central role of glutathione in detoxification of reactive oxygen species and methylglyoxal and in heavy metal chelation. J. Bot. 2012:872875 10.1155/2012/872875

[B175] Hossain Z. Komatsu S. (2013). Contribution of proteomic studies towards understanding plant heavy metal tress response. Front. Plant Sci. 3:310. 10.3389/fpls.2012.00310 23355841 PMC3555118

[B176] Hossain Z. Nouri M. Z. Komatsu S. (2012c). Plant cell organelle proteomics in response to abiotic stress. J. Proteome Res. 11, 37–48. 10.1021/pr200863r 22029473

[B177] Howarth J. R. Domínguez-Solís J. R. Gutíerrez-Alcalá G. Wray J. L. Romero L. C. Gotor C. (2003). The serine acetyltransferase gene family in *Arabidopsis thaliana* and the regulation of its expression by cadmium. Plant Mol. Biol. 51, 589–598. 10.1023/A:1022349623951 12650624

[B178] Husaini Y. Rai L. C. (1991). Studies on nitrogen and phosphorus metabolism and the photosynthetic electron transport system of *Nostoc linckia* under cadmium stress. J. Plant Physiol. 138, 429–435. 10.1016/S0176-1617(11)80518-3

[B179] Hussain D. Haydon M. J. Wang Y. Wong E. Sherson S. M. Young J.. (2004). P-Type ATPase heavy metal transporters with roles in essential zinc homeostasis in Arabidopsis. Plant Cell 16, 1327–1339. 10.1105/tpc.020487 15100400 PMC423219

[B180] Imran M. A. Ch M. N. Khan R. M. Ali Z. Mahmood T. (2013). Toxicity of arsenic (As) on seed germination of sunflower (*Helianthus annuus L*.). Int. J. Phys. Sci. 8, 840–847. 10.5897/IJPS2013.3894

[B181] Indriolo E. Na G. Ellis D. Salt D. E. Banks J. A. (2010). A vacuolar arsenite transporter necessary for arsenic tolerance in the arsenic hyperaccumulating fern *Pteris vittata* is missing in flowering plants. Plant Cell 22, 2045–2057. 10.1105/tpc.109.069773 20530755 PMC2910956

[B182] Ingle R. A. Smith J. A. Sweetlove L. J. (2005). Responses to nickel in the proteome of the hyperaccumulator plant *Alyssum lesbiacum*. Biometal 18, 627–641. 10.1007/s10534-005-2999-0 16388402

[B183] Iqbal N. Nazar R. Khan M. I. R. Masood A. Khan N. A. (2011). Role of gibberellins in regulation of source-sink relations under optimal and limiting environmental conditions. Curr. Sci. 100, 998–1007. Available online at: http://www.currentscience.ac.in/Volumes/100/07/0998.pdf

[B184] Israr M. Sahi S. Datta R. Sarkar D. (2006). Bioaccumulation and physiological effects of mercury in *Sesbania drummonii*. Chemosphere 65, 591–598. 10.1016/j.chemosphere.2006.02.016 16564071

[B185] Iuchi S. Koyama H. Iuchi A. Kobayashi Y. Kitabayashi S. Kobayashi Y.. (2007). Zinc finger protein STOP1 is critical for proton tolerance in *Arabidopsis* and coregulates a key gene in aluminum tolerance. Proc. Natl. Acad. Sci. U.S.A. 104, 9900–9905. 10.1073/pnas.0700117104 17535918 PMC1887543

[B186] Jalloh M. A. Chen J. Zhen F. Zhang G. (2009). Effect of different N fertilizer forms on anti-oxidant capacity and grain yield of rice growing under Cd stress. J. Hazard. Mater. 162, 1081–1085. 10.1016/j.jhazmat.2008.05.146 18603363

[B187] Janeckzo A. Koscielniak J. Pilipowicz M. Szarek-Lukaszewska G. Skoczowski A. (2005). Protection of winter rape photosystem 2 by 24-epibrassinolide under cadmium stress. Photosynthetica 43, 293–298. 10.1007/s11099-005-0048-4

[B188] Janicka-Russak M. Kabała K. Burzyński M. Kłobus G. (2008). Response of plasma membrane H^+^-ATPase to heavy metal stress in *Cucumis sativus* roots. J. Exp. Bot. 59, 3721–3728. 10.1093/jxb/ern219 18820260 PMC2561156

[B189] Jarup L. (2003). Hazards of heavy metal contamination. Br. Med. Bull. 68, 167–182. 10.1093/bmb/ldg032 14757716

[B190] Jewell M. C. Campbell B. C. Godwin I. D. (2010). Transgenic plants for abiotic stress resistance, in Transgenic Crop Plants, eds Kole C. Michler C. H. Abbott A. G. Hall T. C. (Berlin; Heidelberg: Springer-Verlag), 67–31. 10.1007/978-3-642-04812-8_2

[B191] Jonak C. Nakagami H. Hirt H. (2004). Heavy metal stress. Activation of distinct mitogen-activated protein kinase pathways by copper and cadmium. Plant Physiol. 136, 3276–3283. 10.1104/pp.104.045724 15448198 PMC523386

[B192] Jonak C. Okrész L. Bögre L. Hirt H. (2002). Complexity, cross talk and integration of plant MAP kinase signalling. Curr. Opin. Plant Biol. 5, 415–424. 10.1016/S1369-5266(02)00285-6 12183180

[B193] Jun M. Fu H. Y. Hong J. Wan X. Yang C. S. Ho C. T. (2003). Comparison ofantioxidant activities of isoflavones from kudzu root (PuerarialobataOhwi). J. Food Sci. 68, 2117–2122. 10.1111/j.1365-2621.2003.tb07029.x

[B194] Kagale S. Divi U. K. Krochko J. E. Keller W. A. Krishna P. (2007). Brassinosteroid confers tolerance in *Arabidopsis thaliana* and *Brassica napus* to a range of abiotic stresses. Planta 225, 353–364. 10.1007/s00425-006-0361-6 16906434

[B195] Kanoun-Boulé M. Vicente J. A. Nabais C. Prasad M. N. V. Freitas H. (2009). Ecophysiological tolerance of duckweeds exposed to copper. Aquat. Toxicol. 91, 1–9. 10.1016/j.aquatox.2008.09.009 19027182

[B196] Kavamura V. N. Esposito E. (2010). Biotechnological strategies applied to the decontamination of soils polluted with heavy metal. Biotechnol. Adv. 28, 61–69. 10.1016/j.biotechadv.2009.09.002 19778598

[B197] Kenderešová L. Staňová A. Pavlovkin J. Ďurišová E. Nadubinská M. Ciamporová M.. (2012). Early Zn^2+^-induced effects on membrane potential account for primary heavy metal susceptibility in tolerant and sensitive *Arabidopsis* species. Ann. Bot. 110, 445–459. 10.1093/aob/mcs111 22645116 PMC3394654

[B198] Kerkeb L. Krämer U. (2003). The role of free histidine in xylem loading of nickel in *Alyssum lesbiacum* and *Brassica juncea*. Plant Physiol. 131, 716–724. 10.1104/pp102.010686 12586895 PMC166847

[B199] Keunen E. Remans T. Bohler S. Vangronsveld J. Cuypers A. (2011). Metal-induced oxidative stress and plant mitochondria. Int. J. Mol. Sci. 12, 6894–6918. 10.3390/ijms12106894 22072926 PMC3211017

[B200] Khan A. L. Lee I. J. (2013). Endophytic *Penicillium funiculosum* LHL06 secretes gibberellin that reprograms *Glycine max* L. growth during copper stress. BMC Plant Biol. 13:86. 10.1186/1471-2229-13-86 23721090 PMC3674946

[B201] Khan N. A. Anjum N. A. Nazar R. Iqbal N. (2009). Increased activity of ATP-sulfurylase and increased contents of cysteine and glutathione reduce high cadmium- induced oxidative stress in mustard cultivar with high photosynthetic potential. Russ. J. Plant Physiol. 56, 670–677. 10.1134/S1021443709050136

[B202] Khan N. A. Samiullah Singh S. Nazar R. (2007). Activities of antioxidative enzymes, sulphur assimilation, photosynthetic activity and growth of wheat (*Triticum aestivum*) cultivars differing in yield potential under cadmium stress. J. Agron. Crop Sci. 193, 435–444. 10.1111/j.1439-037X.2007.00272.x

[B203] Khattak R. A. Page A. L. Parker D. R. Bakhtar D. (1991). Accumulation and interactions of arsenic, selenium, molybdenum and phosphorus in Alfalfa. J. Environ. Qual. 20, 165–168. 10.2134/jeq1991.00472425002000010026x

[B204] Khripach V. Zhabinskii V. De Groot A. (2000). Twenty years of brassinosteroids: steroidal plant hormones warrant better crops for the XXI century. Ann. Bot. 86, 441–447. 10.1006/anbo.2000.1227

[B205] Kieffer P. Dommes J. Hoffmann L. Hausman J. F. Renaut J. (2008). Quantitative changes in protein expression of cadmium-exposed poplar plants. Proteomics 8, 2514–2530. 10.1002/pmic.200701110 18563750

[B206] Kikui S. Sasaki T. Maekawa M. Miyao A. Hirochika H. Matsumoto H.. (2005). Physiological and genetic analyses of aluminium tolerance in rice, focusing on root growth during germination. J. Inorg. Biochem. 99, 1837–1844. 10.1016/j.jinorgbio.2005.06.031 16095709

[B207] Kim I. S. Shin S. Y. Kim Y. S. Kim H. Y. Yoon H. S. (2009). Expression of a glutathione reductase from *Brassica rapa* subsp. pekinensis enhanced cellular redox homeostasis by modulating antioxidant proteins in *Escherichia coli*. Mol. Cells 28, 479–487. 10.1007/s10059-009-0168-y 19936628

[B208] Klapheck S. Schlunz S. Bergmann L. (1995). Synthesis of phytochelatins and homo-phytochelatins in *Pisum sativum* L. Plant Physiol. 107, 515–521. 12228379 10.1104/pp.107.2.515 PMC157155

[B209] Kobae Y. Uemura T. Sato M. H. Ohnishi M. Mimura T. Nakagawa T.. (2004). Zinc transporter of *Arabidopsis thaliana* AtMTP1 is localized to vacuolar membranes and implicated in zinc homeostasis. Plant Cell Physiol. 45, 1749–1758. 10.1093/pcp/pci015 15653794

[B210] Koprivova A. North K. A. Kopriva S. (2008). Complex signaling network in regulation of adenosine 5′-phosphosulfate reductase by salt stress in *Arabidopsis* roots. Plant Physiol. 146, 1408–1420. 10.1104/pp.107.113175 18218969 PMC2259037

[B211] Kosová K. Vítámvás P. Prášil I. T. Renaut J. (2011). Plant proteome changes under abiotic stress- contribution of proteomics studies to understanding plant stress response. J. Proteomics 74, 1301–1322. 10.1016/j.jprot.2011.02.006 21329772

[B212] Kováčik J. Klejdus B. (2008). Dynamics of phenolic acids and lignin accumulation in metal-treated Matricariachamomilla roots. Plant Cell Rep. 27, 605–615. 10.1007/s00299-007-0490-9 18066553

[B213] Kováčik J. Klejdus B. Bačkor M. (2009). Phenolic metabolism of Matricariachamomilla plants exposed to nickel. J. Plant Physiol. 166, 1460–1464. 10.1016/j.jplph.2009.03.002 19380176

[B214] Krämer U. (2010). Metal hyperaccumulation in plants. Annu. Rev. Plant Biol. 61, 517–534. 10.1146/annurev-arplant-042809-112156 20192749

[B215] Krämer U. Cotter-Howells J. D. Charnock J. M. Baker A. J. M. Smith J. A. C. (1996). Free histidine as a metal chelator in plants that accumulate nickel. Nature 379, 635–638. 10.1038/379635a0

[B216] Krämer U. Pickering I. J. Prince R. C. Raskin I. Salt D. E. (2000). Subcellular localization and speciation of nickel in hyperaccumulator and non-accumulator Thlaspi species. Plant Physiol. 122, 1343–1354. 10.1104/pp.122.4.1343 10759531 PMC58970

[B217] Krämer U. Talke I. N. Hanikenne M. (2007). Transition metal transport. FEBS Lett. 581, 2263–2272. 10.1016/j.febslet.2007.04.010 17462635

[B218] Krantev A. Yordanova R. Janda T. Szalai G. Popova L. (2008). Treatment with salicylic acid decreases the effect of cadmium on photosynthesis in maize plants. J. Plant Physiol. 165, 920–931. 10.1016/j.jplph.2006.11.014 17913285

[B219] Krishnamurti G. S. R. Cieslinski G. Huang P. M. Van Rees K. C. J. (1997). Kinetics of cadmium release from soils as influenced by organic acids: implication in cadmium availability. J. Environ. Qual. 26, 271–277. 10.2134/jeq1997.00472425002600010038x

[B220] Kumar A. Prasad M. N. V. Sytar O. (2012). Lead toxicity, defense strategies and associated indicative biomarkers in Talinumtriangularegrown hydroponically. Chemosphere 89, 1056–1165. 10.1016/j.chemosphere.2012.05.070 22722003

[B221] Kuriakose S. V. Prasad M. N. V. (2008). Cadmium stress affects seed germination and seedling growth in Sorghum bicolor (L.) Moench by changing the activities of hydrolyzing enzymes. Plant Growth Regul. 54, 143–156. 10.1007/s10725-007-9237-4

[B222] Kwak J. M. Nguyen V. Schroeder J. I. (2006). The role of reactive oxygen species in hormonal responses. Plant Physiol. 141, 323–329. 10.1104/pp.106.079004 16760482 PMC1475468

[B223] Lan H. X. Wang Z. F. Wang Q. H. Wang M. M. Bao Y. M. Huang J.. (2012). Characterization of a vacuolar zinc transporter OZT1 in rice (*Oryza sativa* L.). Mol. Biol. Rep. 40, 1201–1210. 10.1007/s11033-012-2162-2 23070916

[B224] Landrieu I. da Costa M. De Veylder L. Dewitte F. Vandepoele K. Hassan S.. (2004a). A small CDC25 dual-specificity tyrosine-phosphatase isoform in *Arabidopsis thaliana*. Proc. Natl. Acad. Sci. U.S.A. 101, 13380–13385. 10.1073/pnas.0405248101 15329414 PMC516575

[B225] Landrieu I. Hassan S. Sauty M. Dewitte F. Wieruszeski J. M. Inzé D.. (2004b). Characterization of the *Arabidopsis thaliana* Arath;CDC25 dualspecificity tyrosine phosphatase. Biochem. Biophys. Res. Commun. 322, 734–739. 10.1016/j.bbrc.2004.07.182 15336525

[B226] Lanquar V. Lelièvre F. Bolte S. Hamès C. Alcon C. Neumann D.. (2005). Mobilization of vacuolar iron by AtNRAMP3 and AtNRAMP4 is essential for seed germination on low iron. EMBO J. 24, 4041–4051. 10.1038/sj.emboj.7600864 16270029 PMC1356305

[B227] Lauer Júnior C. M. Bonatto D. Mielniczki-Pereira A. A. Schuch A. Z. Dias J. F. Yoneama M. L.. (2008). The Pmr1 protein, the major yeast Ca2+-ATPase in the grefecvolgi, regulates intracellular levels of the cadmium ion. FEMS Microbiol. Lett. 285, 79–88. 10.1111/j.1574-6968.2008.01214.x 18510555

[B228] Lavid N. Schwartz A. Yar Den O. Tel-Or E. (2001). The involvement of polyphenols and peroxidase acitivities in heavy metal accumulation by epidermal glands of water lily (*Nymphaeceaea*). Planta 212, 323. 10.1007/s004250000400 11289596

[B229] Lea P. J. Miflin B. J. (2004). Glutamate synthase and the synthesis of glutamate in plants. Plant Physiol. Biochem. 41, 555–564. 10.1016/S0981-9428(03)00060-3

[B230] Le Martret B. Poage M. Shiel K. Nugent G. D. Dix P. J. (2011). Tobacco chloroplast transformants expressing genes encoding dehydroascorbate reductase, glutathione reductase, and glutathione-S-transferase, exhibit altered anti-oxidant metabolism and improved abiotic stress tolerance. Plant Biotechnol. J. 9, 661–673. 10.1111/j.1467-7652.2011.00611.x 21450042

[B231] LeDuc D. L. AbdelSamie M. Móntes-Bayon M. Wu C. P. Reisinger S. J. Terry N. (2006). Overexpressing both ATP sulfurylase and selenocysteine methyltransferase enhances selenium phytoremediation traits in Indian mustard. Environ. Poll. 144, 70–76. 10.1016/j.envpol.2006.01.008 16515825

[B232] Lee H. Jo J. Son D. (1998). Molecular cloning and characterization of the gene encoding glutathione reductase in *Brassica campestris*. Biochim. Biophys. Acta 1395, 309–314. 10.1016/S0167-4781(97)00198-X 9512665

[B233] Lee K. Bae D. W. Kim S. H. Han H. J. Liu X. Park H. C.. (2010). Comparative proteomic analysis of the short-term responses of rice roots and leaves to cadmium. J. Plant Physiol. 167, 161–168. 10.1016/j.jplph.2009.09.006 19853963

[B234] Lee S. H. Ahsan N. Lee K. W. Kim D. H. Lee D. G. Kwak S. S.. (2007). Simultaneous overexpression of both CuZn superoxide dismutase and ascorbate peroxidase in transgenic tall fescue plants confers increased tolerance to a wide range of abiotic stresses. J. Plant Physiol. 164, 1626–1638. 10.1016/j.jplph.2007.01.003 17360071

[B235] Li L. Huang X. Borthakur D. Ni H. (2012). Photosynthetic activity and antioxidative response of seagrass *Thalassia hemprichii* to trace metal stress. Acta Oceanol. Sin. 31, 98–108. 10.1007/s13131-012-0210-3

[B236] Li P. Chen L. Zhou Y. Xia X. Shi K. Chen Z.. (2013). Brassinosteroids-induced systemic stress tolerance was associated with increased transcripts of several defence-related genes in the phloem in *Cucumis sativus*. PLoS ONE 8:e66582. 10.1371/journal.pone.0066582 23840504 PMC3686678

[B237] Li W. X. Chen T. B. Huang Z. C. Lei M. Liao X. Y. (2006). Effect of arsenic on chloroplast ultrastructure and calcium distribution in arsenic hyperaccumulator *Pteris vittata* L. Chemosphere 62, 803–809. 10.1016/j.chemosphere.2005.04.055 15972226

[B238] Li Y. Dhankher O. P. Carreira L. Lee D. Chen A. Schroeder J. I.. (2004). Overexpression of phytochelatin synthase in *Arabidopsis* leads to enhanced arsenic tolerance and cadmium hypersensitivity. Plant Cell Physiol. 45, 1787–1797. 10.1093/pcp/pch202 15653797

[B239] Liang W. H. Li L. Zhang F. Liu Y. X. Li M. M. Shi H. H. (2013). Effects of abiotic stress, light, phytochromes and phytohormones on the expression of *OsAQP*, a rice aquaporin gene. Plant Growth Regul. 69, 21–27. 10.1007/s10725-012-9743-x

[B240] Liang Zhu Y. Pilon-Smits E. A. H. Jouanin L. Terry N. (1999). Overexpression of glutathione synthetase in Indian mustard enhances cadmium cccumulation and tolerance. Plant Physiol. 119, 173–180. 9880348 10.1104/pp.119.1.73 PMC32244

[B241] Lidon F. C. Henriques F. S. (1991). Limiting step on photosynthesis of rice plants treated with varying copper levels. J. Plant Physiol. 138, 115–118.

[B242] Lin C. C. Kao C. H. (2000). Effect of NaCl stress on H2O2 metabolism in rice leaves. J. Plant Growth Regul. 30, 151–155. 10.1023/A:1006345126589

[B243] Lin T. Zhu X. Zhang F. Wan X. (2011). The detoxification effect of cadmium stress in *Populus yunnanensis*. Res. J. Bot. 4, 13–19. 10.3923/brj.2011.13.19

[B244] Lin Y. F. Liang H. M. Yang S. Y. Boch A. Clemens S. Chen C. C.. (2009). Arabidopsis IRT3 is a zinc-regulated and plasma membrane localized zinc/iron transporter. New Phytol. 182, 392–404. 10.1111/j.1469-8137.2009.02766.x 19210716

[B245] Lingua G. Bona E. Todeschini V. Cattaneo C. Marsano F. Berta G.. (2012). Effects of heavy metal and arbuscular mycorrhiza on the leaf proteome of a selected Poplar clone: A time course analysis. PLoS ONE 7:e38662. 10.1371/journal.pone.0038662 22761694 PMC3383689

[B246] Liu D. Jiang W. Li M. (1992). Effects of trivalent and hexavalent chromium on root growth and cell division of *Allium cepa*. Hereditas 117, 23–29. 10.1111/j.1601-5223.1992.tb00003.x

[B247] Liu G. Y. Zhang Y. X. Chai T. Y. (2011). Phytochelatin synthase of *Thlaspi caerulescens* enhanced tolerance and accumulation of heavy metal when expressed in yeast and tobacco. Plant Cell Rep. 30, 1067–1076. 10.1007/s00299-011-1013-2 21327392

[B248] Liu X. Wu H. Ji C. Wei L. Zhao J. Yu J. (2013). An integrated proteomic and metabolomic study on the chronic effects of mercury in *Suaeda salsa* under an environmentally relevant salinity. PLoS ONE 8:e64041. 10.1371/journal.pone.0064041 23696864 PMC3655940

[B249] Loeffler S. Hochberger A. Grill E. Winnacker E.-L. Zenk M.-H. (1989). Termination of the phytochelatin synthase reaction through sequestration of heavy metals by the reaction product. FEBS Lett. 258, 42–46. 10.1016/0014-5793(89)81611-4

[B250] Long T. A. Tsukagoshi H. Busch W. Lahner B. Salt D. E. Benfey P. N. (2010). The bHLH transcription factor POPEYE regulates response to iron deficiency in *Arabidopsis* roots. Plant Cell 22, 2219–2236. 10.1105/tpc.110.074096 20675571 PMC2929094

[B251] López-Millán A. F. Ellis D. R. Grusak M. A. (2004). Identification and characterization of several new members of the ZIP family of metal ion transporters in Medicagotruncatula. Plant Mol. Biol. 54, 583–596. 10.1023/B:PLAN.0000038271.96019.aa 15316291

[B252] Lu Y. P. Li Z. S. Rea P. A. (1997). AtMRP1 gene of Arabidopsis encodes a glutathione S-conjugate pump: isolation and functional definition of a plant ATP-binding cassette transporter gene. Proc. Natl. Acad. Sci. U.S.A. 94, 8243–8248. 10.1073/pnas.94.15.8243 9223346 PMC21588

[B253] Lu Y. P. Li Z. S. Drozdowicz Y. M. Hortensteiner S. Martinoia E. Rea P. A. (1998). AtMRP2, an Arabidopsis ATP binding cassette transporter able to transport glutathione S-conjugates and chlorophyll catabolites: functional comparisons with AtMRP1. Plant Cell 10, 267–282. 9490749 10.1105/tpc.10.2.267 PMC143980

[B254] Lushchak V. I. Semchuk N. M. (2012). Tocopherol biosynthesis: chemistry, regulation and effects of environmental factors. Acta Physiol. Plant. 34, 1607–1628. 10.1007/s11738-012-0988-9

[B255] Ma J. F. Yamaji N. Mitani N. Xu X. Y. Su Y. H. McGrath S. P.. (2008). Transporters of arsenite in rice and their role in arsenic accumulation in rice grain. Proc. Natl. Acad. Sci. U.S.A. 105, 9931–9935. 10.1073/pnas.0802361105 18626020 PMC2481375

[B256] Ma L. Q. Komar K. M. Tu C. Zhang W. Cai Y. Kennelly E. D. (2001). A fern that hyperaccumulates arsenic. Nature 409, 579. 10.1038/35054664 11214308

[B257] Maleva M. G. Nekrasova G. F. Borisova G. G. Chukina N. V. Ushakova O. S. (2012). Effect of heavy metal on photosynthetic apparatus and antioxidant status of elodea. Russ. J. Plant Physiol. 59, 190–197. 10.1134/S1021443712020069

[B258] Manavalan L. P. Guttikonda S. K. Tran L.-S. P. Nguyen H. T. (2009). Physiological and molecular approaches to improve drought resistance in soybean. Plant Cell Physiol. 50, 1260–1276. 10.1093/pcp/pcp082 19546148

[B259] Marschner H. (1995). Mineral Nutrition of Higher Plants. Boston, MA: Academic Press.

[B260] Mäser P. Thomine S. Schroeder J. I. Ward J. M. Hirschi K. Sze H.. (2001). Phylogenetic relationships within cation transporter families of *Arabidopsis*. Plant Physiol. 126, 1646–1667. 10.1104/pp.126.4.1646 11500563 PMC117164

[B261] Masood A. Iqbal N. Khan N. A. (2012). Role of ethylene in alleviation of cadmium-induced photosynthetic capacity inhibition by sulfur in mustard. Plant Cell Environ. 35, 524–533. 10.1111/j.1365-3040.2011.02432.x 21950968

[B262] Mathys W. (1977). The role of malate, oxalate, and mustard oil glucosides in the evolution of zinc resistance in herbage plants. Physiol. Plant. 40, 130–136. 10.1111/j.1399-3054.1977.tb01509.x

[B263] Matsuoka M. (2003). Gibberellin signaling: how do plant cells respond to GA signals? J. Plant Growth Regul. 22, 123–125. 10.1007/s00344-003-0039-2

[B264] Matusik J. Bajda T. Manecki M. (2008). Immobilization of aqueous cadmium by addition of phosphates. J. Hazard. Mater. 152, 1332–1339. 10.1016/j.jhazmat.2007.08.010 17868991

[B265] McLaughlin M. J. Parker D. R. Clark J. M. (1999). Metal and micronutrients-food safety issues. Field Crops Res. 60, 143–163. 10.1016/S0378-4290(98)00137-3

[B266] Meharg A. A. Hartley-Whitaker J. (2002). Arsenic uptake and metabolism in arsenic resistant and non resistant plant species. New Phytol. 154, 29–43. 10.1046/j.1469-8137.2002.00363.x

[B267] Meister A. Anderson M. E. (1983). Glutathione. Annu. Rev. Biochem. 52, 711–760. 10.1146/annurev.bi.52.070183.003431 6137189

[B268] Meng H. Hua S. Shamsi I. H. Jilani G. Li Y. Jiang L. (2009). Cadmium-induced stress on the seed germination and seedling growth of *Brassica napus* L., and its alleviation through exogenous plant growth regulators. Plant Growth Regul. 58, 47–59. 10.1007/s10725-008-9351-y

[B269] Menguer P. K. Farthing E. Peaston K. A. Ricachenevsky F. K. Fett J. P. Williams L. E. (2013). Functional analysis of the rice vacuolar zinc transporter OsMTP1. J. Exp. Bot. 64, 2871–2883. 10.1093/jxb/ert136 23761487 PMC3697945

[B270] Métraux J. P. (2002). Recent breakthroughs in the study of salicylic acid biosynthesis. Trends Plant. Sci. 7, 332–334. 10.1016/S1360-1385(02)02313-0 12167322

[B271] Metwally A. Finkemeier I. Georgi M. Dietz K. J. (2003). Salicylic acid alleviates the cadmium toxicity in barley seedlings. Plant Physiol. 132, 272–281. 10.1104/pp.102.018457 12746532 PMC166972

[B272] Miller G. Shulaev V. Mittler R. (2008). Reactive oxygen signaling and abiotic stress. Physiol. Plant 133, 481–489. 10.1111/j.1399-3054.2008.01090.x 18346071

[B273] Miller G. Suzuki N. Ciftci-Yilmaz S. Mittler R. (2010). Reactive oxygen species homeostasis and signaling during drought and salinity stresses. Plant Cell Environ. 33, 453–467. 10.1111/j.1365-3040.2009.02041.x 19712065

[B274] Mills R. F. Francini A. da Rocha P. S. C. F. Baccarini P. J. Aylett M. Krijger G. C.. (2005). The plant P1B-type ATPase AtHMA4 transports Zn and Cd and plays a role in detoxification of transition metals supplied at elevated levels. FEBS Lett. 579, 783–791. 10.1016/j.febslet.2004.12.040 15670847

[B275] Milner M. J. Kochian L. V. (2008). Investigating heavy-metal hyperaccumulation using *Thlaspi caerulescens* as a model system. Ann. Bot. 102, 3–13. 10.1093/aob/mcn063 18440996 PMC2712422

[B276] Milner M. J. Seamon J. Craft E. Kochian L. V. (2013). Transport properties of members of the ZIP family in plants and their role in Zn and Mn homeostasis. J. Exp. Bot. 64, 369–381. 10.1093/jxb/ers315 23264639 PMC3528025

[B277] Miransari M. (2011). Hyperaccumulators, arbuscular mycorrhizal fungi and stress of heavy metal. Biotechnol. Adv. 29, 645–653. 10.1016/j.biotechadv.2011.04.006 21557996

[B278] Mishra S. Tripathi R. D. Srivastava S. Dwivedi S. Trivedi P. K. Dhankher O. P. (2009a). Thiol metabolism play significant role during Cd detoxification by *Ceratophyllum demersum* L. Biores. Tech. 100, 2155–2161. 10.1016/j.biortech.2008.10.041 19091554

[B279] Mishra Y. Chaurasia N. Rai L. C. (2009b). AhpC (alkyl hydroperoxide reductase) from *Anabaena* sp. PCC 7120 protects *Escherichia coli* from multiple abiotic stresses. Biochem. Biophys. Res. Commun. 381, 606–611. 10.1016/j.bbrc.2009.02.100 19248767

[B280] Mittler R. (2002). Oxidative stress, antioxidants and stress tolerance. Trends Plant Sci. 7, 405–410. 10.1016/S1360-1385(02)02312-9 12234732

[B281] Mittler R. Vanderauwera S. Gollery M. Van Breusegem F. (2004). Reactive oxygen gene network of plants. Trends Plant Sci. 9, 490–498. 10.1016/j.tplants.2004.08.009 15465684

[B282] Mizuno T. Usui K. Horie K. Nosaka S. Mizuno N. Obata H. (2005). Cloning of three ZIP/Nramp transporter genes from a Ni hyperaccumulator plant *Thlaspi japonicum* and their Ni^2+^-transport abilities. Plant Physiol. Biochem. 43, 793–801. 10.1016/j.plaphy.2005.07.006 16198592

[B283] Møller I. M. Jensen P. E. Hansson A. (2007). Oxidative modifications to cellular components in plants. Annu. Rev. Plant Biol. 58, 459–481. 10.1146/annurev.arplant.58.032806.103946 17288534

[B284] Monnet F. Vaillant N. Vernay P. Coudret A. Sallanon H. Hitmi A. (2001). Relationship between PSII activity, CO2 fixation, and Zn, Mn and Mg contents of *Lolium perenne* under zinc stress. J. Plant Physiol. 158, 1137–1144. 10.1078/S0176-1617(04)70140-6

[B285] Montanini B. Blaudez D. Jeandroz S. Sanders D. Chalot M. (2007). Phylogenetic and functional analysis of the Cation Diffusion Facilitator (CDF) family. Improved signature and prediction of substrate specificity. BMC Genomics 8:107. 10.1186/1471-2164-8-107 17448255 PMC1868760

[B286] Morel M. Crouzet J. Gravot A. Auroy P. Leonhardt N. Vavasseur A.. (2009). AtHMA3, a P1B-ATPase allowing Cd/Zn/Co/Pb vacuolar storage in *Arabidopsis*. Plant Physiol. 149, 894–904. 10.1104/pp.108.130294 19036834 PMC2633814

[B287] Morita S. Tasaka M. Fujisawa H. Ushimaru T. Tsuji H. (1994). A cDNA clone encoding a rice catalase isozyme. Plant Physiol. 105, 1015–1016. 10.1104/pp.105.3.1015 8058828 PMC160753

[B288] Mukhopadhyay A. Vij S. Tyagi A. K. (2004). Overexpression of a zinc-finger protein gene from rice confers tolerance to cold, dehydration, and salt stress in transgenic tobacco. Proc. Natl. Acad. Sci. U.S.A. 101, 6309–6314. 10.1073/pnas.0401572101 15079051 PMC395965

[B289] Munne-Bosch S. (2005). The role of a9-tocopherol in plant stress tolerance. J. Plant Physiol. 162, 743–748. 10.1016/j.jplph.2005.04.022 16008098

[B290] Murphy A. Taiz L. (1995). Comparison of metallothionein gene expression and nonprotein thiols in ten *Arabidopsis* ecotypes. Plant Physiol. 109, 945–954. 10.1104/pp.109.3.945 8552721 PMC161396

[B291] Muthuchelian K. Bertamini M. Nedunchezhian N. (2001). Triacontanol can protect Erythrina variegata from cadmium toxicity. J. Plant Physiol. 158, 1487–1490. 10.1078/0176-1617-00627

[B292] Nada E. Ferjani B. A. Ali R. Imed B. R. B. M. Makki B. (2007). Cadmium-induced growth inhibition and alteration of biochemical parameters in almond seedlings grown in solution culture. Acta Physil. Plant. 29, 57–62. 10.1007/s11738-006-0009-y

[B293] Nagajyoti P. C. Lee K. D. Sreekanth T. V. M. (2010). Heavy metal, occurrence and toxicity for plants: a review. Environ. Chem. Lett. 8, 199–216. 10.1007/s10311-010-0297-8

[B294] Naika M. Shameer K. Mathew O. K. Gowda R. Sowdhamini R. (2013). STIFDB2: an updated version of plant stress-responsive transcription factor database with additional stress signals, stress-responsive transcription factor binding sites and stress-responsive genes in *Arabidopsis* and rice. Plant Cell Physiol. 54:e8. 10.1093/pcp/pcs185 23314754 PMC3583027

[B295] Nakano T. Suzuki K. Fujimura T. Shinshi H. (2006). Genomewide analysis of the ERF gene family in Arabidopsis and rice. Plant Physiol. 140, 411–432. 10.1104/pp.105.073783 16407444 PMC1361313

[B296] Nakashima K. Yamaguchi-Shinozaki K. (2006). Regulons involved in osmotic stress-responsive and cold stress-responsive gene expression in plants. Physiol. Plant. 126, 62–71. 10.1111/j.1399-3054.2005.00592.x

[B297] Nakashima K. Ito Y. Yamaguchi-Shinozaki K. (2009). Transcriptional regulatory networks in response to abiotic stresses in *Arabidopsis* and grasses. Plant Physiol. 149, 88–95. 10.1104/pp.108.129791 19126699 PMC2613698

[B298] Nan Z. Li J. Zhang J. Cheng G. (2002). Cadmium and zinc interactions and their transfer in soil–crop system under actual field conditions. Sci. Total Environ. 285, 187–195. 10.1016/S0048-9697(01)00919-6 11874041

[B299] NandaKumar P. B. A. Dushenkov V. Motto H. Raskin I. (1995). Phytoextraction: the use of plants to remove heavy metal from soils. Environ. Sci. Technol. 29, 1232–1238. 10.1021/es00005a014 22192016

[B300] Neill S. Desikan R. Hancock J. (2002). Hydrogen peroxide signaling. Curr. Opin. Plant Biol. 5, 388–395. 10.1016/S1369-5266(02)00282-0 12183176

[B301] Nies D. H. Silver S. (1995). Ion efflux systems involved in bacterial metal resistances. J. Ind. Microbiol. 14, 186–199. 10.1007/BF01569902 7766211

[B302] Opdenakker K. Remans T. Keunen E. Vangronsveld J. Cuypers A. (2012). Exposure of *Arabidopsis thaliana* to Cd or Cu excess leads to oxidative stress mediated alterations in MAPKinase transcript levels. Environ. Exp. Bot. 83, 53–61. 10.1016/j.envexpbot.2012.04.003

[B303] Ortiz D. F. Kreppel L. Speiser D. M. Scheel G. McDonald G. Ow D. W. (1992). Heavy metal tolerance in the fission yeast requires an ATP-binding cassette-type vacuolar membrane transporter. EMBO J. 11, 3491–3499. 1396551 10.1002/j.1460-2075.1992.tb05431.x PMC556806

[B304] Ortiz D. F. Ruscitti T. McCue K. F. Ow D. W. (1995). Transport of metal-binding peptides by HMT1, a fission Yeast ABC-type vacuolar membrane protein. J. Biol. Chem. 270, 4721–4728. 10.1074/jbc.270.9.4721 7876244

[B305] Pal'ove-Balang P. Kisová A. Pavlovkin J. Mistrík I. (2006). Effect of manganese on cadmium toxicity in maize seedlings. Plant Soil Environ. 52, 143–149. Available online at: http://www.agriculturejournals.cz/publicFiles/50533.pdf

[B306] Panda S. K. Baluska F. Matsumoto H. (2009). Aluminum stress signaling in plants. Plant Signal. Behav. 4, 592–597. 10.4161/psb.4.7.8903 19820334 PMC2710549

[B307] Pankovic D. Plesnicar M. Arsenijeevic-Maksimovic I. Petrovic N. Sakac Z. Kastori R. (2000). Effects of nitrogen nutrition on photosynthesis in Cd-treated sunflower plants. Ann. Bot. 86, 841–847. 10.1006/anbo.2000.1250

[B308] Papoyan A. Kochian L. V. (2004). Identification of *Thlaspi caerulescens* genes that may be involved in heavy metal hyperaccumulationand tolerance. Characterization of a novel heavy metal transporting ATPase. Plant Physiol. 136, 3814–3823. 10.1104/pp.104.044503 15516513 PMC527178

[B309] Pawlak-Sprada S. Arasimowicz-Jelonek M. Podgórska M. Deckert J. (2011). Activation of phenylopropanoid pathway in legume plants exposed to heavy metals. Part I. Effects of cadmium and lead on phenylalanine ammonia-lyase gene expression, enzyme activity and lignin content. Acta. Biochim. Pol. 58, 211–216. 21503278

[B310] Peiter E. Montanini B. Gobert A. Pedas P. Husted S. Maathuis F. J. M.. (2007). A secretory pathway-localized cation diffussion facilitator confers plant manganese tolerance. Proc. Natl. Acad. Sci. U.S.A. 104, 8532–8537. 10.1073/pnas.0609507104 17494768 PMC1895984

[B311] Peleg Z. Blumwald E. (2011). Hormone balance and abiotic stress tolerance in crop plants. Curr. Opin. Plant Biol. 14, 290–295. 10.1016/j.pbi.2011.02.001 21377404

[B312] Pena L. B. Barcia R. A. Azpilicueta C. E. Méndez A. A. Gallego S. M. (2012). Oxidative post translational modifications of proteins related to cell cycle are involved in cadmium toxicity in wheat seedlings. Plant Sci. 196, 1–7. 10.1016/j.plantsci.2012.07.008 23017894

[B313] Pence N. S. Larsen P. B. Ebbs S. D. Lasat M. M. Letham D. L. D. Garvin D. F.. (2000). The molecular basis for heavy metal hyperaccumulation in *Thlaspi caerulescens*. Proc. Natl. Acad. Sci. U.S.A. 97, 4956–4960. 10.1073/pnas.97.9.4956 10781104 PMC18339

[B314] Peng H. Y. Yang X. E. Jiang L. Y. (2005). Copper phytoavailability and uptake by *Elsholtzi asplendens* from contaminated soil as affected by soil amendments. J. Environ. Sci. Health 40, 839–856. 10.1081/ESE-200048283 15792303

[B315] Petö A. Lehotai N. Lozano-Juste J. León J. Tari I. Erdei L.. (2011). Involvement of nitric oxide and auxin in signal transduction of copper-induced morphological responses in *Arabidopsis* seedlings. Ann. Bot. 108, 449–457. 10.1093/aob/mcr176 21856638 PMC3158692

[B316] Pilon-Smits E. A. H. Hwang S. Lytle C. M. Zhu Y. Tai J. C. Bravo R. C.. (1999). Overexpression of ATP sulfurylase in indian mustard leads to increased selenate uptake, reduction, and tolerance. Plant Physiol. 119, 1123–1132. 10.1104/pp.119.1.123 9880353 PMC32211

[B317] Pilon-Smits E. A. H. Zhu Y. L. Sears T. Terry N. (2000). Overexpression of glutathione reductase in *Brassica juncea*: effects on cadmium accumulation and tolerance. Physiol. Plant. 110, 455–460. 10.1111/j.1399-3054.2000.1100405.x

[B318] Podar D. Scherer J. Noordally Z. Herzyk P. Nies D. Sanders D. (2012). Metal selectivity determinants in a family of transition metal transporters. J. Biol. Chem. 287, 3185–3196. 10.1074/jbc.m111.305649 22139846 PMC3270973

[B319] Pomponi M. Censi V. Di Girolamo V. De Paolis A. di Toppi L. S. Aromolo R.. (2006). Overexpression of *Arabidopsis* phytochelatin synthase in tobacco plants enhances Cd(2+) tolerance and accumulation but not translocation to the shoot. Planta 223, 180–190. 10.1007/s00425-005-0073-3 16133212

[B320] Popova L. P. Maslenkova L. T. Yordanova R. Y. Ivanova A. P. Krantev A. P. Szalai G.. (2009). Exogenous treatment with salicylic acid attenuates cadmium toxicity in pea seedlings. Plant Physiol. Biochem. 47, 224–231. 10.1016/j.plaphy.2008.11.007 19091585

[B321] Prasad A. S. (2012). Discovery of human zinc deficiency: 50 years later. J. Trace Elem. Med. Biol. 26, 66–69. 10.1016/j.jtemb.2012.04.004 22664333

[B322] Prasad M. N. V. Freitas H. Fraenzle S. Wuenschmann S. Markert B. (2010). Knowledge explosion in phytotechnologies for environmental solutions. Environ. Pollut. 158, 18–23. 10.1016/j.envpol.2009.07.038 19683373

[B323] Qiu Z. Z. Guan Z. Y. Long C. Y. (2005). Effect of zinc on cadmium uptake by spring wheat (*Triticum aestivum* L.): long time hydroponic study and short time 109Cd tracing study. J. Zhejiang Univ. Sci. 6, 643–648. 10.1007/BF02856167

[B324] Qureshi M. I. D'Amici G. M. Fagioni M. Rinalducci S. Zolla L. (2010). Iron stabilizes thylakoid protein-pigment complexes in indian mustard during Cd-phytoremediation as revealed by BN-SDS-PAGE and ESI-MS/MS. J. Plant Physiol. 167, 761–770. 10.1016/j.jplph.2010.01.017 20199821

[B325] Rai V. K. (2002). Role of amino acids in plant responses to stress. Biol. Plant. 45, 481–487. 10.1023/A:1022308229759

[B326] Ramos J. Clemente M. R. Naya L. Loscos J. Perez-Rontome C. Sato S. (2007). Phytochelatin synthases of the model legume *Lotus japonicus*. A small multigene family with different responses to cadmium and alternatively spiced variants. Plant Physiol. 143, 110–118. 10.1104/pp.106.090894 PMC1820930 17208961

[B327] Rascio N. (1997). Metal accumulation by some plants growing on zinc-mine deposits. Oikos 29, 250–253. 10.2307/3543610

[B328] Rauser W. E. (1995). Phytochelatins and related peptides. Structure, biosynthesis, and function. Plant Physiol. 109, 1141–1149. 10.1104/pp.109.4.1141 8539285 PMC157644

[B329] Rauser W. E. (1999). Structure and function of metal chelators produced by plants. The case for organic acids, amino acids, phytin and metallothioneins. Cell Biochem. Biophys. 31, 19–48. 10.1007/BF02738153 10505666

[B330] Ravet K. Touraine B. Boucherez J. Briat J. F. Gaymard F. Cellier F. (2009). Ferritins control interaction between iron homeostasis and oxidative stress in Arabidopsis. Plant J. 57, 400–412. 10.1111/j.1365-313X.2008.03698.x 18826427

[B331] Reeves R. D. (2006). Hyperaccumulation of trace elements by plants, in Phytoremediation of Metal-Contaminated Soils, NATO Science Series: IV: Earth and Environmental Sciences, eds Morel J. L. Echevarria G. Goncharova N. (New York, NY: Springer), 25–52.

[B332] Ricachenevsky F. K. Menguer P. K. Sperotto R. A. Williams L. E. Fett J. P. (2013). Roles of plant metal tolerance proteins (MTP) in metal storage and potential use in biofortification strategies. Front. Plant Sci 4:144. 10.3389/fpls.2013.00144 23717323 PMC3653063

[B333] Ritter A. Ubertini M. Romac S. Gaillard F. Delage L. Mann A.. (2010). Copper stress proteomics highlights local adaptation of two strains of the model brown alga *Ectocarpus siliculosus*. Proteomics 10, 2074–2088. 10.1002/pmic.200900004 20373519

[B334] Rivas-San Vicente M. Plasencia J. (2011). Salicylic acid beyond defence: its role in plant growth and development. J. Exp. Bot. 62, 3321–3338. 10.1093/jxb/err031 21357767

[B335] Rodríguez-Serrano M. Romero-Puertas M. C. Pazmiňo D. M. Testillano P. S. Risueňo M. C. Del Río L. A.. (2009). Cellular response of pea plants to cadmium toxicity: cross talk between reactive oxygen species, nitric oxide, and calcium. Plant Physiol. 150, 229–243. 10.1104/pp.108.131524 19279198 PMC2675729

[B336] Roth U. von Roepenack-Lahaye E. Clemens S. (2006). Proteome changes in *Arabidopsis thaliana* roots upon exposure to Cd^2+^. J. Exp. Bot. 57, 4003–4013. 10.1093/jxb/erl170 17075075

[B337] Ruiz O. N. Alvarez D. Torres C. Roman L. Daniell H. (2011). Metallothionein expression in chloroplasts enhances mercury accumulation and phytoremediation capability. Plant Biotechnol. J. 9, 609–617. 10.1111/j.1467-7652.2011.00616.x 21518240 PMC4522697

[B338] Safarzadeh S. Ronaghi A. Karimian N. (2013). Effect of cadmium toxicity on micronutrient concentration, uptake and partitioning in seven rice cultivars. Arch. Agron. Soil Sci. 59, 231–245. 10.1080/03650340.2011.622752

[B339] Saito A. Saito M. Ichikawa Y. Yoshiba M. Tadano T. Miwa E.. (2010). Difference in the distribution and speciation of cellular nickel between nickel-tolerant and non-tolerant *Nicotiana tabacum* L. cv. BY-2 cells. Plant Cell Environ. 33, 174–187. 10.1111/j.1365-3040.2009.02068.x 19906154

[B340] Salt D. E. Rauser W. E. (1995). MgATP-dependent transport of phytochelatins across the tonoplast of oat roots. Plant Physiol. 107, 1293–1301. 12228436 10.1104/pp.107.4.1293 PMC157264

[B341] Salt D. E. Baxter I. Lahner B. (2008). Ionomics and the study of the plant ionome. Annu. Rev. Plant Biol. 59, 709–733. 10.1146/annurev.arplant.59.032607.092942 18251712

[B342] Salt D. E. Prince R. C. Baker A. J. M. Raskin I. Pickering I. J. (1999). Zinc ligands in the metal accumulator *Thlaspi caerulescensas* determined using X-ray absorption spectroscopy. Environ. Sci. Technol. 33, 713–717. 10.1021/es980825x

[B343] Sánchez-Bermejo E. Castrillo G. del Llano B. Navarro C. Zarco-Fernández S. Martinez- Herrera D. J.. (2014). Natural variation in arsenate tolerance identifies an arsenate reductase in *Arabidopsis thaliana*. Nat. Commun. 5:4617. 10.1038/ncomms5617 25099865

[B344] Sánchez-Pardo B. Fernández-Pascual M. Zornoza P. (2012). Copper microlocalisation, ultrastructural alterations and antioxidant responses in the nodules of white lupin and soybeanplants grown under conditions of copper excess. Environ. Exp. Bot. 84, 52–60. 10.1016/j.envexpbot.2012.04.017

[B345] Sanita di Toppi L. Gabbrielli R. (1999). Response to cadmium in higher plants. Environ. Exp. Bot. 41, 105–130. 10.1016/S0098-8472(98)00058-6

[B346] Sano T. Yoshihara T. Handa K. Sato M. H. Nagata T. Hasezawa S. (2012). Metal ion homeostasis mediated by NRAMP transporters in plant cells-focused on increased resistance to iron and cadmium ion, in Crosstalk and Integration of Membrane Trafficking Pathways, ed Weigert R. (InTech), 213–228. Available online at: http://www.intechopen.com/books/crosstalk-and-integration-of-membrane-trafficking-pathways/metal-ion-homeostasis-mediated-by-nramp-transporters-in-plant-cells-focused-on-increased-resistance-

[B347] Sarret G. Saumitou-Laprade P. Bert V. Proux O. Hazemann J. L. Traverse A.. (2002). Forms of zinc accumulated in the hyperaccumulator *Arabidopsis halleri*. Plant Physiol. 130, 1815–1826. 10.1104/pp.007799 12481065 PMC166693

[B348] Sarwar N. Saifullah Malhi S. S. Zia M. H. Naeem A. Bibia S. Farida G. (2010). Role of mineral nutrition in minimizing cadmium accumulation by plants. J. Sci. Food Agric. 90, 925–937. 10.1002/jsfa.3916 20355131

[B349] Sasaki A. Yamaji N. Yokosho K. Ma J. F. (2012). Nramp5 is a major transporter responsible for manganese and cadmium uptake in rice. Plant Cell. 24, 2155–2167. 10.1105/tpc.112.096925 22589467 PMC3442593

[B350] Schafer C. Simper H. Hofmann B. (1992). Glucose feeding results in coordinated changes of chlorophyll content, ribulose-1,5-biphosphate carboxylase-oxygenase activity and photosynthetic potential photoautotrophic suspension cultured cells of *Chenopodium ruburum*. Plant Cell Environ. 15, 343–350.

[B351] Schaaf G. Honsbein A. Meda A. R. Kirchner S. Wipf D. von Wirén N. (2006). AtIREG2 encodes a tonoplast transport protein involved in iron-dependent nickel detoxification in *Arabidopsis thaliana* roots. J. Biol. Chem. 281, 25532–25540. 10.1074/jbc.M601062200 16790430

[B352] Scholz G. Schlesier G. Seifert K. (1985). Effect of nicotianamine on iron uptake by the tomato mutant ‘chloronerva’. Physiol. Plant. 63, 99–104. 10.1111/j.1399-3054.1985.tb02825.x

[B353] Schützendübel A. Polle A. (2002). Plant responses to abiotic stresses: heavy metal-induced oxidative stress and protection by mycorrhization. J. Exp. Bot. 53, 1351–1365. 10.1093/jexbot/53.372.1351 11997381

[B354] Schwarz K. Foltz C. M. (1957). Selenium as an integral part of factor 3 against dietary necrotic liver degeneration. J. Am. Chem. Soc. 70, 3292–3293. 10.1021/ja01569a087 10408880

[B355] Semane B. Dupae J. Cuypers A. Noben J. P. Tuomainen M. Tervahauta A.. (2010). Leaf proteome responses of *Arabidopsis thaliana* exposed to mild cadmium stress. J. Plant Physiol. 167, 247–254. 10.1016/j.jplph.2009.09.015 20005002

[B356] Shahid M. Pinelli E. Dumat C. (2012). Review of Pb availability and toxicity to plants in relation with metal speciation; role of synthetic and natural organic ligands. J. Hazard. Mater. 219–220, 1–12. 10.1016/j.jhazmat.2012.01.060 22502897

[B357] Shahzad Z. Gosti F. Frerot H. Lacombe E. Roosens N. Saumitou- Laprade P.. (2010). The five AhMTP1 zinc transporters undergo different evolutionary fates towards adaptive evolution to zinc tolerance in *Arabidopsis halleri*. PLoS Genet. 6:e1000911. 10.1371/journal.pgen.1000911 20419142 PMC2855318

[B358] Shameer K. Ambika S. Varghese S. M. Karaba N. Udayakumar M. Sowdhamini R. (2009). STIFDB–*Arabidopsis* stress-responsive transcription factor DataBase. Int. J. Plant Genomics 2009:583429. 10.1155/2009/583429 19841686 PMC2763139

[B359] Shanker K. Mishra S. Srivastava S. Srivastava R. Daas S. Prakash S. (1996). Effect of selenite and selenate on plant uptake and translocation of mercury by tomato (*Lycopersicum esculentum*). Plant Soil 183, 233–238. 10.1007/BF00011438

[B360] Shanmugam V. Lo J. C. Wu C. L. Wang S. L. Lai C. C. Connolly E. L.. (2011). Differential expression and regulation of iron regulated metal transporters in *Arabidopsis halleri* and *Arabidopsis thaliana*—the role in zinc tolerance. New Phytol. 190, 125–137. 10.1111/j.1469-8137.2010.03606.x 21219335

[B361] Sharaf A. E. M. M. Farghal I. I. Sofy M. R. (2009). Role of gibberellic acid in abolishing the detrimental effects of Cd and Pb on broad bean and Lupin plants. Res. J. Agric. Biol. Sci. 5, 668–673. Available online at: https://www.researchgate.net/publication/242631571

[B362] Sharma I. Pati P. K. Bhardwaj R. (2011). Effect of 28-homobrassinolide on antioxidant defence system in *Raphanus sativus* L. under chromium toxicity. Ecotoxicology 20, 862–874. 10.1007/s10646-011-0650-0 21448625

[B363] Sharma P. Bhardwaj R. (2007). Effects of 24-epibrassinolide on growth and metal uptake *Brassica juncea* L. under copper metal stress. Acta Physiol. Plant. 29, 259–263. 10.1007/s11738-007-0032-7

[B364] Sharma P. Bhardwaj R. Arora N. Arora H. K. Kumar A. (2008). Effects of 28-homobrassinolide on nickel uptake, protein content and antioxidative defence system in *Brassica juncea*. Biol. Plant 52, 767–770. 10.1007/s10535-008-0149-6

[B365] Sharma S. S. Dietz K. J. (2009). The relationship between metal toxicity and cellular redox imbalance. Trends Plant Sci. 14, 43–50. 10.1016/j.tplants.2008.10.007 19070530

[B366] Sharma S. S. Dietz K. J. (2006). The significance of amino acids and amino acid-derived molecules in plant responses and adaptation to heavy metal stress. J. Exp. Bot. 57, 711–726. 10.1093/jxb/erj073 16473893

[B367] Sharmin S. A. Alam I. Kim K. H. Kim Y. G. Kim P. J. Bahk J. D.. (2012). Chromium-induced physiological and proteomic alterations in roots of *Miscanthus sinensis*. Plant Sci. 187, 113–126. 10.1016/j.plantsci.2012.02.002 22404839

[B368] Shen W. Nada K. Tachibana S. (2000). Involvement of polyamines in the chilling tolerance of cucumber cultivars. Plant Physiol. 124, 431–439. 10.1104/pp.124.1.431 10982456 PMC59156

[B369] Shi Q. Zhu Z. (2008). Effects of exogenous salicylic acid on manganese toxicity, element contents and antioxidative system in cucumber. Environ. Exp. Bot. 63, 317–326. 10.1016/j.envexpbot.2007.11.003

[B370] Shi X. H. Zhang C. H. Wang H. Zhang F. S. (2005). Effect of Si on the distribution of Cd in rice seedlings. Plant Soil 272, 53 10.1007/s11104-004-3920-2

[B371] Shim D. Hwang J. U. Lee J. Lee S. Choi Y. An G.. (2009). Orthologs of the class A4 heat shock transcription factor HsfA4a confer cadmium tolerance in wheat and rice. Plant Cell 21, 4031–4043. 10.1105/tpc.109.066902 20028842 PMC2814514

[B372] Shin S. Y. Kim I. S. Kim Y. H. Park H. M. Lee J. Y. Kang H. G.. (2008). Scavenging reactive oxygen species by rice dehydroascorbate reductase alleviates oxidative stresses in *Escherichia coli*. Mol. Cells 26, 616–620. 19011360

[B373] Shiu S. H. Shih M. C. Li W. H. (2005). Transcription factor families have much higher expansion rates in plants than in animals. Plant Physiol. 139, 18–26. 10.1104/pp.105.065110 16166257 PMC1203354

[B374] Siborova M. (1988). Cd2^+^ ions affect the quaternary structure of ribulose-1,5-bisphosphate carboxylase from barley leaves. Biochem. Physiol. Pflanzen 183, 371–378. 10.1016/S0015-3796(88)80045-3

[B375] Siddiqui M. H. Al-Whaibi M. H. Basalah M. O. (2011). Interactive effect of calcium and gibberellin on nickel tolerance in relation to antioxidant systems in *Triticum aestivum* L. Protoplasma 248, 503–511. 10.1007/s00709-010-0197-6 20730631

[B376] Silva S. (2012). Aluminium toxicity targets in plants. J. Bot. 2012:219462 10.1155/2012/219462

[B377] Simões C. C. Melo J. O. Magalhaes J. V. Guimarães C. T. (2012). Genetic and molecular mechanisms of aluminum tolerance in plants. Genet. Mol. Res. 11, 1949–1957. 10.4238/2012.July.19.14 22869550

[B378] Singh K. Foley R. C. Oñate-Sánchez L. (2002). Transcription factors in plant defense and stress responses. Curr. Opin. Plant Biol. 5, 430–436. 10.1016/S1369-5266(02)00289-3 12183182

[B379] Singh N. Ma L. Q. Vu J. C. Raj A. (2009). Effects of arsenic on nitrate metabolism in arsenic hyperaccumulating and non-hyperaccumulating ferns. Environ. Pollut. 157, 2300–2305. 10.1016/j.envpol.2009.03.036 19406540

[B380] Singh R. K. Anandhan S. Singh S. Patade V. Y. Ahmed Z. Pande V. (2011a). Metallothionein-like gene from *Cicer microphyllum*is regulated by multiple abiotic stresses. Protoplasma 248, 839–847. 10.1007/s00709-010-0249-y 21161305

[B381] Singh V. P. Singh S. Kumar J. Prasad S. M. (2015). Investigating the roles of ascorbate-glutathione cycle and thiol metabolism in arsenate tolerance in ridged Luffa seedlings. Protoplasma 252, 1217–1229. 10.1007/s00709-014-0753-6 25586108

[B382] Singh V. P. Srivastava P. K. Prasad S. M. (2012). Differential effect of UV-B radiation on growth, oxidative stress and ascorbate-glutathione cycle in two cyanobacteria under copper toxicity. Plant Physiol. Biochem. 61, 61–70. 10.1016/j.plaphy.2012.09.005 23063802

[B383] Singh V. P. Srivatava P. K. Prasad S. M. (2013). Nitric oxide alleviates arsenic-induced toxic effects in ridged Luffa seedlings. Plant. Physiol. Biochem. 71, 155–163. 10.1016/j.plaphy.2013.07.003 23917073

[B384] Singh V. P. Tripathi D. K. Kumar D. Chauhan D. K. (2011b). Influence of exogenous silicon addition on aluminium tolerance in rice seedlings. Biol. Trace Elem. Res. 144, 1260–1274. 10.1007/s12011-011-9118-6 21681465

[B385] Siripornadulsil S. Traina S. Verma D. P. S. Sayre R. T. (2002). Molecular mechanisms of proline-mediated tolerance to toxic heavy metal in transgenic microalgae. Plant Cell 14, 2837–2847. 10.1105/tpc.004853 12417705 PMC152731

[B386] Smith P. Gregory P. J. van Vuuren D. Obersteiner M. Havlík P. Rounsevell M.. (2010). Competition for land. Philos. Trans R. Soc. B Biol. Sci. 365, 2941–2957. 10.1098/rstb.2010.0127 20713395 PMC2935113

[B387] Sobkowiak R. Deckert J. (2006). Proteins induced by cadmium in soybean cells. J. Plant Physiol. 163, 1203–1206. 10.1016/j.jplph.2005.08.017 17032622

[B388] Song A. Li Z. Zhang J. Xue G. Fan F. Liang Y. (2009). Silicon-enhanced resistance to cadmium toxicity in *Brassica chinensis* L. is attributed to Si-suppressed cadmium uptake and transport and Si-enhanced antioxidant defense capacity. J. Hazard. Mater. 172, 74–83. 10.1016/j.jhazmat.2009.06.143 19616891

[B389] Song W.-Y. Yamaki T. Yamaji N. Ko D. Jung K. H. Fujii-Kashino M.. (2014). A rice ABC transporter, OsABCC1, reduces arsenic accumulation in the grain. PNAS 111, 15699–15704. 10.1073/pnas.1414968111 25331872 PMC4226097

[B390] Sooksa-Nguan T. Yakubov B. Kozlovskyy V. I. Barkume C. M. Howe K. J. Thannhauser T. W.. (2009). Drosophila ABC transporter, DmHMT-1, confers tolerance to cadmium. DmHMT-1 and its yeast homolog, SpHMT-1, are not essential for vacuolar phytochelatin sequestration. J. Biol. Chem. 284, 354–362. 10.1074/jbc.M806501200 19001374

[B391] Srivastava G. Kumar S. Dubey G. Mishra V. Prasad S. M. (2012). Nickel and ultraviolet-B stresses induce differential growth and photosynthetic responses in *Pisum sativum* L. seedlings. Biol. Trace Elem. Res. 149, 86–96. 10.1007/s12011-012-9406-9 22528776

[B392] Srivastava S. Srivastava A. K. Suprasanna P. D'souza S. F. (2013). Identification and profiling of arsenic stress-induced microRNAs in *Brassica juncea*. J. Exp. Bot. 64, 303–315. 10.1093/jxb/ers333 23162117

[B393] Stephan U. W. Scholz G. (1993). Nicotianamine: mediator of transport of iron and heavy metals in the phloem? Physiol. Plant. 88, 522–529.

[B394] Street R. A. Kulkarni M. G. Stirk W. A. Southway C. Van Staden J. (2010). Effect of cadmium on growth and micronutrient distribution in wild garlic (*Tulbaghia violacea*). South Afr. J. Bot. 76, 332–336. 10.1016/j.sajb.2009.12.006

[B395] Strobel N. E. Kuc A. (1995). Chemical and biological inducers ofsystemic acquired resistance to pathogens protect cucumber andtobacco from damage caused by paraquat and cupric chloride. Phytopathol 85, 1306–1310. 10.1094/Phyto-85-1306

[B396] Sundaramoorthy P. Chidambaram A. Ganesh K. S. Unnikannan P. Baskaran L. (2010). Chromium stress in paddy: (i) nutrient status of paddy under chromium stress; (ii) phytoremediation of chromium by aquatic and terrestrial weeds. C. R. Biol. 333, 597–607. 10.1016/j.crvi.2010.03.002 20688280

[B397] Suzuki N. (2005). Alleviation by calcium of cadmium induced root growth inhibition in *Arabidopsis* seedlings. Plant Biotechnol. 22, 19–25. 10.5511/plantbiotechnology.22.19

[B398] Takahashi H. Kawakatsu T. Wakasa Y. Hayashi S. Takaiwa F. (2012). A rice transmembrane bZIP transcription factor, OsbZIP39, regulates the endoplasmic reticulum stress response. Plant Cell Physiol. 53, 144–153. 10.1093/pcp/pcr157 22084314

[B399] Talanova V. V. Titov A. F. Boeva N. P. (2000). Effect of increasing concentration of lead and cadmium on cucumber seedlings. Biol. Plant. 43, 441–444. 10.1023/A:1026735603890

[B400] Talke I. N. Hanikenne M. Krämer U. (2006). Zinc-dependent global transcriptional control, transcriptional deregulation, and higher gene copy number for genes in metal homeostasis of the hyperaccumulator *Arabidopsis halleri*. Plant Physiol. 142, 148–167. 10.1104/pp.105.076232 16844841 PMC1557598

[B401] Tamás L. Mistrík I. Huttová J. Halusková L. Valentovicová K. Zelinová V. (2010). Role of reactive oxygen species-generating enzymes and hydrogen peroxide during cadmium, mercury and osmotic stresses in barley root tip. Planta 231, 221–231. 10.1007/s00425-009-1042-z 19898864

[B402] Tang W. Charles T. M. Newton R. J. (2005). Overexpression of the pepper transcription factor CaPF1 in transgenic *Virginia pine* (*Pinus Virginiana* Mill.) confers multiple stress tolerance and enhances organ growth. Plant Mol. Biol. 59, 603–617. 10.1007/s11103-005-0451-z 16244910

[B403] Thomine S. Lelièvre F. Debarbieux E. Schroeder J. I. Barbier-Brygoo H. (2003). AtNRAMP3, a multispecificvacuolar metal transporter involved in plant responses to iron deficiency. Plant J. 34, 685–695. 10.1046/j.1365-313X.2003.01760.x 12787249

[B404] Thomine S. Wang R. Ward J. M. Crawford N. M. Schroeder J. I. (2000). Cadmium and iron transport by members of a plant transporter gene family in Arabidopsis with homology to NRAMP genes. Proc. Nat. Acad. Sci. U.S.A. 97, 4991–4996. 10.1073/pnas.97.9.4991 PMC18345 10781110

[B405] Thounaojam T. C. Panda P. Mazumdar P. Kumar D. Sharma G. D. Sahoo L.. (2012). Excess copper induced oxidative stress and response of antioxidants in rice. Plant Physiol. Biochem. 53, 33–39. 10.1016/j.plaphy.2012.01.006 22306354

[B406] Tiwari M. Sharma D. Dwivedi S. Singh M. Tripathi R. D. Trivedi P. K. (2014). Expression in *Arabidopsis* and cellular localization reveal involvement of rice NRAMP, OsNRAMP1, in arsenic transport and tolerance. Plant Cell Environ. 37, 140–152. 10.1111/pce.12138 23700971

[B407] Tkalec M. Štefanić P. P. Cvjetko P. Šikić S. Pavlica M. Balen B. (2014). The effects of cadmium-zinc interactions on biochemical responses in tobacco seedlings and adult plants. PLoS ONE 9:e87582. 10.1371/journal.pone.0087582 24475312 PMC3903775

[B408] Tommey A. M. Shi J. Lindsay W. P. Urwin P. E. Robinson N. J. (1991). Expression of the pea gene PsMTA in E. coli. Metal binding properties of the expressed protein. FEBS Lett. 292, 48–52. 10.1016/0014-5793(91)80831-M 1959626

[B409] Tran L. S. P. Nishiyama R. Yamaguchi-Shinozaki K. Shinozaki K. (2010). Potential utilization of NAC transcription factors to enhance abiotic stress tolerance in plants by biotechnological approach. GM Crops 1, 32–39. 10.4161/gmcr.1.1.10569 21912210

[B410] Treeby M. Marschner H. Römheld V. (1989). Mobilization of iron and other micronutrient cations from a calcareous soil by plant-borne, microbial, and synthetic metal chelators. Plant Soil 114, 217–226. 10.1007/BF02220801

[B411] Tripathi D. K. Singh V. P. Kumar D. Chauhan D. K. (2012). Impact of exogenous silicon addition on chromium uptake, growth, mineral elements, oxidative stress, antioxidant capacity, and leaf and root structures in rice seedlings exposed to hexavalent chromium. Acta Physiol. Plant. 34, 279–289. 10.1007/s11738-011-0826-5

[B412] Tseng M. J. Liu C. W. Yiu J. C. (2007). Enhanced tolerance to sulfur dioxide and salt stress of transgenic Chinese cabbage plants expressing both superoxide dismutase and catalase in chloroplasts. Plant Physiol. Biochem. 45, 822–833. 10.1016/j.plaphy.2007.07.011 17851086

[B413] Tsukagoshi H. Busch W. Benfey P. N. (2010). Transcriptional regulation of ROS controls transition from proliferation to differentiation in the root. Cell 143, 606–616. 10.1016/j.cell.2010.10.020 21074051

[B414] Tu C. Ma L. Q. (2005). Effects of arsenic on concentration and distribution of nutrients in the fronds of the arsenic hyperaccumulator *Pteris vittata* L. Environ. Pollut. 135, 333–340. 10.1016/j.envpol.2004.03.026 15734593

[B415] Tuomainen M. H. Nunan N. Lehesranta S. J. Tervahauta A. I. Hassinen V. H. Schat H.. (2006). Multivariate analysis of protein profiles of metal hyperaccumulator *Thlaspi caerulescens* accessions. Proteomics 6, 3696–3706. 10.1002/pmic.200501357 16691554

[B416] Umezawa T. Fujita M. Fujita Y. Yamaguchi-Shinozaki K. Shinozaki K. (2006). Engineering drought tolerance in plants: discovering and tailoring genes to unlock the future. Curr. Opin. Biotechnol. 17, 113–122. 10.1016/j.copbio.2006.02.002 16495045

[B417] Valliyodan B. Nguyen H. T. (2006). Understanding regulatory networks and engineering for enhanced drought tolerance in plants. Curr. Opin. Plant Biol. 9, 189–195. 10.1016/j.pbi.2006.01.019 16483835

[B418] van de Mortel J. E. Villanueva L. A. Schat H. Kwekkeboom J. Coughlan S. Moerland P. D.. (2006). Large expression differences in genes for iron and zinc homeostasis, stress response, and lignin biosynthesis distinguish roots of *Arabidopsis thaliana* and the related metal hyperaccumulator *Thlaspi caerulescens*. Plant Physiol. 142, 1127–1134. 10.1104/pp.106.082073 16998091 PMC1630723

[B419] van der Zaal B. J. Neuteboom L. W. Pinas J. E. Chardonnens A. N. Schat H. Verkleij J. A.. (1999). Overexpression of a novel *Arabidopsis* gene related to putative zinc-transporter genes from animals can lead to enhanced zinc resistance and accumulation. Plant Physiol. 119, 1047–1055. 10.1104/pp.119.3.1047 10069843 PMC32086

[B420] Varotto C. Maiwald D. Pesaresi P. Jahns P. Salamini F. Leister D. (2002). The metal ion transporter IRT1 is necessary for iron homeostasis and efficient photosynthesis in *Arabidopsis thaliana*. Plant J. 31, 589–599. 10.1046/j.1365-313X.2002.01381.x 12207649

[B421] Vassilev A. Yordanov I. (1997). Reductive analysis of factors limiting growth of cadmium-treated plants: a review. Bulg. J. Plant Physiol. 23, 114–133.

[B422] Vatamaniuk O. K. Bucher E. A. Sundaram M. V. Rea P. A. (2005). CeHMT-1, a putative phytochelatin transporter, is required for cadmium tolerance in *Caenorhabditis elegans*. J. Biol. Chem. 280, 23684–23690. 10.1074/jbc.M503362200 15840570

[B423] Verbruggen N. Hermans C. Schat H. (2009). Molecular mechanisms of metal hyperaccumulation in plants. New Phytol. 181, 759–776. 10.1111/j.1469-8137.2008.02748.x 19192189

[B424] Vert G. Barberon M. Zelazny E. Séguéla M. Briat J. F. Curie C. (2009). Arabidopsis IRT2 cooperates with the high-affinity iron uptake system to maintain iron homeostasis in root epidermal cells. Planta 229, 1171–1179. 10.1007/s00425-009-0904-8 19252923

[B425] Vert G. Grotz N. Dédaldéchamp F. Gaymard F. Guerinot M. L. Briat J. F.. (2002). IRT1, an *Arabidopsis* transporter essential for iron uptake from the soil and for plant growth. Plant Cell 14, 1223–1233. 10.1105/tpc.001388 12084823 PMC150776

[B426] Villiers F. Jourdain A. Bastien O. Leonhardt N. Fujioka S. Tichtincky G.. (2012). Evidence for functional interaction between brassinosteroids and cadmium response in *Arabidopsis thaliana*. J. Exp. Bot. 63, 1185–1200. 10.1093/jxb/err335 22131160

[B427] Vinocur B. Altman A. (2005). Recent advances in engineering plant tolerance to abiotic stress: achievements and limitations. Curr. Opin. Biotechnol. 16, 123–132. 10.1016/j.copbio.2005.02.001 15831376

[B428] Vögeli-Lange R. Wagner G. J. (1990). Subcellular localization of cadmium and cadmium-binding peptides in tobacco leaves. Implication of a transport function for cadmium-binding peptides. Plant Physiol. 92, 1086–1093. 10.1104/pp.92.4.1086 16667375 PMC1062420

[B429] Vriet C. Russinova E. Reuzeau C. (2012). Boosting crop yields with plant steroids. Plant Cell 24, 842–857. 10.1105/tpc.111.094912 22438020 PMC3336137

[B430] Walliwalagedara C. van Keulen H. Willard B. Wei R. (2012). Differential proteome analysis of *Chlamydomonas reinhardtii* response to arsenic exposure. Am. J. Plant Sci. 3, 764–772. 10.4236/ajps.2012.36092

[B431] Wang H. Y. Klatte M. Jakoby M. Bäumlein H. Weisshaar B. Bauer P. (2007). Iron deficiency-mediated stress regulation of four subgroup Ib BHLH genes in *Arabidopsis thaliana*. Planta 226, 897–908. 10.1007/s00425-007-0535-x 17516080

[B432] Wang H. Zhao S. C. Liu R. L. Zhou W. Jin J. Y. (2009). Changes of photosynthetic activities of maize (*Zea mays* L.) seedlings in response to cadmium stress. Photosynthetica 47, 277–283. 10.1007/s11099-009-0043-2

[B433] Wang R. Gao F. Guo B. G. Huang J. C. Wang L. Zhou Y. J. (2013). Short-term chromium-stress-induced alterations in the maize leaf proteome. Int. J. Mol. Sci. 14, 11125–11144. 10.3390/ijms140611125 23712354 PMC3709723

[B434] Wang S. H. Yang Z. M. Yang H. Lu B. Li S. Q. Lu Y. P. (2004). Copper induced stress and antioxidative responses in roots of *Brassica juncea* L. Bot. Bull. Acad. Sin. 45, 203–212. Available online at: http://ejournal.sinica.edu.tw/bbas/content/2004/3/Bot453-04.pdf

[B435] Wang Y. Hu H. Zhu L. Y. Li X. X. (2012). Response to nickel in the proteome of the metal accumulator plant *Brassica juncea*. J. Plant Interact. 7, 230–237. 10.1080/17429145.2011.603060

[B436] Wangeline A. L. Burkhead J. L. Hale K. L. Lindblom S. D. Terry N. Pilon M.. (2004). Over- expression of ATP sulfurylase in indian mustard: effects on tolerance and accumulation of twelve metals. J. Environ. Qual. 33, 54–60. 10.2134/jeq2004.5400 14964358

[B437] Wani P. A. Khan M. S. Zaidi A. (2012). Toxic effects of heavy metal on germination and physiological processes of plants, in Toxicity of Heavy Metal to Legumes and Bioremediation, eds Zaidi A. Wani P. A. Khan M. S. (Springer-Verlag Wien), 45–66.

[B438] Warne M. S. Heemsbergen D. Stevens D. McLaughlin M. Cozens G. Whatmuff M.. (2008). Modeling the toxicity of copper and zinc salts to wheat in 14 soils. Environ. Toxicol. Chem. 27, 786–792. 10.1897/07-294.1 18333681

[B439] Weber M. Trampczynska A. Clemens S. (2006). Comparative transcriptome analysis of toxic metal responses in *Arabidopsis thaliana* and the Cd^2+^-hypertolerant facultative metallophyte *Arabidopsis halleri*. Plant Cell Environ. 29, 950–963. 10.1111/j.1365-3040.2005.01479.x 17087478

[B440] Whiting S. N. Leake J. R. McGrath S. P. Baker A. J. M. (2000). Positive responses to zinc and cadmium by roots of the hyperaccumulator *Thlaspi caerulescens*. New Phytol. 145, 199–210. 10.1046/j.1469-8137.2000.00570.x

[B441] Wilkinson S. Kudoyarova G. R. Veselov D. S. Arkhipova T. N. Davies W. J. (2012). Plant hormone interactions: innovative targets for crop breeding an and management. J. Exp. Bot. 63, 3499–3509. 10.1093/jxb/ers148 22641615

[B442] Williams L. E. Pittman J. K. (2010). Dissecting pathways involved in manganese homeostasis and stress in higher plants, in Cell Biology of Metals and Nutrients, Plant Cell Monographs, Vol. 17, eds Hell R. Mendal R. R. (Berlin, Heidelberg: Springer-Verlag), 95–117.

[B443] Williams L. E. Pittman J. K. Hall J. L. (2000). Emerging mechanisms for heavy metal transport in plants. Biochim. Biophys. Acta 1465, 104–126. 10.1016/S0005-2736(00)00133-4 10748249

[B444] Winkel-Shirley B. (2002). Biosynthesis of flavonoids and effects of stress. Curr. Opin. Plant Biol. 5, 218. 10.1016/S1369-5266(02)00256-X 11960739

[B445] Wong C. K. E. Cobbett C. S. (2009). HMA P-Type ATPases are the major mechanism for root-to-shoot Cd translocation in *Arabidopsis thaliana*. New Phytol. 181, 71–78. 10.1111/j.1469-8137.2008.02638.x 19076718

[B446] Wray G. A. Hahn M. W. Abouheif E. Balhoff J. P. Pizer M. Rockman M. V.. (2003). The evolution of transcriptional regulation in eukaryotes. Mol. Biol. Evol. 20, 1377–1419. 10.1093/molbev/msg140 12777501

[B447] Wu F. B. Zhang G. P. (2002). Genotypic variation in kernel heavy metal concentrations in barley and as affected by soil factors. J. Plant Nutr. 25, 1163–1173. 10.1081/PLN-120004380

[B448] Wu H. Chen C. Du J. Liu H. Cui Y. Zhang Y.. (2012). Co-overexpression FIT with AtbHLH38 or AtbHLH39 in *Arabidopsis*-enhanced cadmium tolerance via increased cadmium sequestration in roots and improved iron homeostasis of shoots. Plant Physiol. 158, 790–800. 10.1104/pp.111.190983 22184655 PMC3271767

[B449] Xia X. J. Wang Y. J. Zhou Y. H. Tao Y. Mao W. H. Shi K.. (2009). Reactive oxygen species are involved in brassinosteroid-induced stress tolerance in cucumber. Plant Physiol. 150, 801–814. 10.1104/pp.109.138230 19386805 PMC2689980

[B450] Xia X. J. Zhou Y. H. Ding J. Shi K. Asami T. Chen Z.. (2011). Induction of systemic stress tolerance by brassinosteroid in *Cucumis sativus*. New Phytol. 191, 706–720. 10.1111/j.1469-8137.2011.03745.x 21564100

[B451] Xia Z. Sun K. Wang M. Wu K. Zhang H. Wu J. (2012). Overexpression of a maize sulfite oxidase gene in tobacco enhances tolerance to sulfite stress via sulfite oxidation and CAT-mediated H_2_O_2_ scavenging. PLoS ONE 7:e37383. 10.1371/journal.pone.0037383 22693572 PMC3365070

[B452] Xiang C. Oliver D. J. (1998). Glutathione metabolic genes coordinately respond to heavy metal and jasmonic acid in *Arabidopsis*. Plant Cell 10, 1539–1550. 10.1105/tpc.10.9.1539 9724699 PMC144077

[B453] Xu J. Tian Y. S. Peng R. H. Xiong A. S. Zhu B. Jin X. F.. (2009). Yeast copper-dependent transcription factor ACE1 enhanced copper stress tolerance in *Arabidopsis*. BMB Rep. 42, 752–757. 10.5483/BMBRep.2009.42.11.752 19944018

[B454] Xu L. L. Fan Z. Y. Dong Y. J. Kong J. Bai X. Y. (2014). Effects of exogenous salicylic acid and nitric oxide on physiological characteristics of two peanut cultivars under cadmium stress. Biol. Plant. 59, 171–182. 10.1007/s10535-014-0475-9

[B455] Yadav S. K. (2010). Heavy metal toxicity in plants: an overview on the role of glutathione and phytochelatins in heavy metal stress tolerance of plants. South Afr. J. Bot. 76, 167–179. 10.1016/j.sajb.2009.10.007

[B456] Yamaji N. Huang C. F. Nagao S. Yano M. Sato Y. Nagamura Y.. (2009). A zinc finger transcription factor ART1 regulates multiple genes implicated in aluminum tolerance in rice. Plant Cell 21, 3339–3349. 10.1105/tpc.109.070771 19880795 PMC2782276

[B457] Yang M. J. Lin X. Y. Yang X. E. (1998). Impact of Cd on growth and nutrient accumulation of different plant species. Chin. J. Appl. Ecol. 9, 89–94.

[B458] Yang X. E. Long X. X. Ni W. Z. (2002). Physiological and molecular mechanisms of heavy metal uptake by hyperaccumulating plant species. J. Plant Nutr. Fert. 8, 8–15. Available online at: http://www.ipublishing.co.in/ijesarticles/thirteen/articles/volthree/EIJES31135.pdf

[B459] Yang X. Feng Y. He Z. Stoffell P. J. (2005). Molecular mechanisms of heavy metal hyperaccumulation and phytoremediation. J. Trace Elem. Med. Biol. 18, 339–353. 10.1016/j.jtemb.2005.02.007 16028496

[B460] Yang Y. Qi M. Mei C. (2004). Endogenous salicylic acid protects rice plants from oxidative damage caused by aging as well as biotic and abiotic stress. Plant J. 40, 909–919. 10.1111/j.1365-313X.2004.02267.x 15584956

[B461] Yang Z. M. Wang J. Wang S. H. Xu L. L. (2003). Salicylic acid-induced aluminum tolerance by modulation of citrate efflux from roots of *Cassia tora* L. Planta 217, 168–174. 10.1007/s00425-003-0980-0 12721861

[B462] Yin L. Wang S. Eltayeb A. E. Uddin M. I. Yamamoto Y. Tsuji W.. (2010). Overexpression of dehydroascorbate reductase, but not monodehydroascorbate reductase, confers tolerance to aluminum stress in transgenic tobacco. Planta 231, 609–621. 10.1007/s00425-009-1075-3 19960204

[B463] Yuan H. M. Xu H. H. Liu W. C. Lu Y. T. (2013). Copper regulates primary root elongation through PIN1-mediated auxin redistribution. Plant Cell Physiol. 54, 766–778. 10.1093/pcp/pct030 23396597

[B464] Yuan L. Yuan Y. Du J. Sun J. Guo S. (2012). Effects of 24-epibrassinolide on nitrogen metabolism in cucumber seedlings under Ca(NO_3_)_2_ stress. Plant Physiol. Biochem. 61, 29–35. 10.1016/j.plaphy.2012.09.004 23031845

[B465] Yuan Y. X. Zhang J. Wang D. W. Ling H.-Q. (2005). AtbHLH29 of *Arabidopsis thaliana* is a functional ortholog of tomato FER involved in controlling iron acquisition in strategy I plants. Cell Res. 15, 613–621. 10.1038/sj.cr.7290331 16117851

[B466] Yuan Y. Wu H. Wang N. Li J. Zhao W. Du J.. (2008). FIT interacts with AtbHLH38 and AtbHLH39 in regulating iron uptake gene expression for iron homeostasis in Arabidopsis. Cell Res. 18, 385–397. 10.1038/cr.2008.26 18268542

[B467] Yusuf M. A. Kumar D. Rajwanshi R. Strasser R. J. Tsimilli-Michael M. Govindjee. (2010). Overexpression of c-tocopherol methyl transferase gene in transgenic *Brassica juncea* plants alleviates abiotic stress: physiological and chlorophyll a fluorescence measurements. Biochim. Biophys. Acta 1797, 1428–1438. 10.1016/j.bbabio.2010.02.002l 20144585

[B468] Yusuf M. Fariduddin Q. Ahmad A. (2012a). 24-Epibrassinolide modulates growth, nodulation, antioxidant system, and osmolyte in tolerant and sensitive varieties of *Vigna radiata* under different levels of nickel: a shotgun approach. Plant Physiol. Biochem. 57, 143–153. 10.1016/j.plaphy.2012.05.004 22705589

[B469] Yusuf M. Fariduddin Q. Varshney P. Ahmad A. (2012b). Salicylic acid minimizes nickel and/or salinity-induced toxicity in Indian mustard (*Brassica juncea*) through an improved antioxidant system. Environ. Sci. Pollut. Res. 19, 8–18. 10.1007/s11356-011-0531-3 21637971

[B470] Zaccheo P. Laura C. Valeria D. M. P. (2006). Ammonium nutrition as a strategy for cadmium metabolisation in the rhizosphere of sunflower. Plant Soil 283, 43–56. 10.1007/s11104-005-4791-x

[B471] Zhang A. Zhang J. Zhang J. Ye N. Zhang H. Tan M.. (2011). Nitric oxide mediates brassinosteroid-induced ABA biosynthesis involved in oxidative stress tolerance in maize leaves. Plant Cell Physiol. 52, 181–192. 10.1093/pcp/pcq187 21134899

[B472] Zhang G. Chen M. Li L. Xu Z. Chen X. Guo J.. (2009a). Overexpression of the soybean *GmERF3* gene, an AP2/ERF type transcription factor for increased tolerances to salt, drought, and diseases in transgenic tobacco. J. Exp. Bot. 60, 3781–3796. 10.1093/jxb/erp214 19602544 PMC2736888

[B473] Zhang H. Lian C. Shen Z. (2009b). Proteomic identification of small, copper-responsive proteins in germinating embryos of *Oryza sativa*. Ann. Bot. 103, 923–930. 10.1093/aob/mcp012 19201764 PMC2707895

[B474] Zhang Y. Li J. Yu F. Cong L. Wang L. Burkard G.. (2006). Cloning and expression analysis of SKn-type dehydrin gene from bean in response to heavy metal. Mol. Biotechnol. 32, 205–217. 10.1385/MB:32:3:205 16632887

[B475] Zhang Y. Yu Z. Fu X. Liang C. (2002). Noc3p, a bHLH protein, plays an integral role in the initiation of DNA replication in budding yeast. Cell 109, 849–860. 10.1016/S0092-8674(02)00805-X 12110182

[B476] Zhang Z. C. Chen B. X. Qiu B. S. (2010). Phytochelatin synthesis plays a similar role in shoots of the cadmium hyperaccumulator *Sedum alfredii* as in non-resistant plants. Plant Cell Environ. 33, 1248–1255. 10.1111/j.1365-3040.2010.02144.x 20233337

[B477] Zhao L. Sun Y. L. Cui S. X. Chen M. Yang H. M. Liu H. M.. (2011). Cd-induced changes in leaf proteome of the hyperaccumulator plant *Phytolacca americana*. Chemosphere 85, 56–66. 10.1016/j.chemosphere.2011.06.029 21723586

[B478] Zhao Z. Q. Zhu Y. G. Li H. Y. Smith S. E. Smith F. A. (2004). Effects of forms and rates of potassium fertilizers on cadmium uptake by two cultivars of spring wheat (*Triticum aestivum* L.). Environ. Int. 29, 973–978. 10.1016/S0160-4120(03)00081-3 14592574

[B479] Zheng X. W. Qiu R. L. Ying R. R. Tang Y. T. Tang L. Fang X. H. (2011). The differentially-expressed proteome in Zn/Cd hyperaccumulator *Arabis paniculata* Franch. in response to Zn and Cd. Chemosphere 82, 321–328. 10.1016/j.chemosphere.2010.10.030 21074242

[B480] Zhenyan H. E. Jiangchuan L. I. Zhang H. Ma M. I. (2005). Different effects of calcium and lanthanum on the expression of phytochelatin synthase gene and cadmium absorption in *Lactuca sativa*. Plant Sci. 168, 309–318. 10.1016/j.plantsci.2004.07.001

[B481] Zhigang A. Cuijie L. Yuangang Z. Yejie D. Wachter A. Gromes R. (2006). Expression of BjMT2, a metallothionein 2 from *Brassica juncea*, increases copper and cadmium tolerance in *Escherichia coli* and *Arabidopsis thaliana*, but inhibits root elongation in *Arabidopsis thaliana* seedlings. J. Exp. Bot. 57, 3575–3582. 10.1093/jxb/erl102 16957018

[B482] Zhou J. Goldsbrough P. B. (1994). Functional homologs of fungal metallothionein genes from Arabidopsis. Plant Cell 6, 875–884. 10.1105/tpc.6.6.875 8061521 PMC160485

[B483] Zhou Z. S. Guo K. Elbaz A. A. Yang Z. M. (2009). Salicylic acid alleviates mercury toxicity by preventing oxidative stress in roots of *Medicago sativa*. Environ. Exp. Bot. 65, 27–34. 10.1016/j.envexpbot.2008.06.001

[B484] Zhu E. Liu D. Li J. G. Li T. Q. Yang X. E. He Z. L. (2011). Effect of nitrogen fertilizer on growth and cadmium accumulation in *Sedum alfredii* Hance. J. Plant Nutr. 34, 115–126. 10.1080/01904167.2011.531363

[B485] Zhu X. F. Jiang T. Wang Z. W. Lei G. J. Shi Y. Z. Li G. X. (2012). Gibberellic acid alleviates cadmium toxicity by reducing nitric oxide accumulation and expression of IRT1 in *Arabidopsis thaliana*. J. Hazard. Mater. 239–240, 302–307. 10.1016/j.jhazmat.2012.08.077 23021314

[B486] Zimeri A. M. Dhankher O. P. McCaig B. Meagher R. B. (2005). The plant MT1 metallothioneins are stabilized by binding cadmium and are required for cadmium tolerance and accumulation. Plant Mol. Biol. 58, 839–855. 10.1007/s11103-005-8268-3 16240177

[B487] Zobel R. W. Kinraide T. B. Baligar V. C. (2007). Fine root diameters can change in response to changes in nutrient concentrations. Plant Soil 297, 243–254. 10.1007/s11104-007-9341-2

[B488] Zornoza P. Sánchez-Pardo B. Carpena R. R. O. (2010). Interaction and accumulation of manganese and cadmium in the manganese accumulator *Lupinus albus*. J. Plant Physiol. 167, 1027–1032. 10.1016/j.jplph.2010.02.011 20399531

